# Heat Therapy: Targeting Health, Disease, and Disability

**DOI:** 10.1002/cph4.70089

**Published:** 2026-01-20

**Authors:** Rauchelle E. Richey, Robert D. Hyldahl, Brendan W. Kaiser, Paige C. Geiger, John R. Halliwill, Christopher T. Minson

**Affiliations:** ^1^ Bowerman Sports Science Center, Department of Human Physiology University of Oregon Eugene Oregon USA; ^2^ Department of Exercise Sciences Brigham Young University Provo Utah USA; ^3^ Department of Cell Biology and Physiology University of Kansas Medical Center Kansas City Kansas USA

## Abstract

Heat therapy is a historic modality that has been used as a source of lifestyle intervention and community for many different cultures. Over the last ~40 years, heat therapy has gained increasing popularity among scientists and clinicians as a potential therapeutic tool for aging and disease. Recently, several systematic reviews and meta‐analyses have sought to encompass specific aspects investigated in the scientific literature surrounding this ancient therapeutic modality, with each review having a primary focus on one beneficial aspect of heat therapy. This review aimed to provide a more comprehensive review of the scientific literature on heat therapy. To accomplish this, we have included studies that demonstrate clear beneficial adaptations (and those that show no effect of heat therapy) on specific organs, crosstalk between different organs and tissues, and integrated physiological systems and pathways. Furthermore, we also discuss what forms of heat therapy confer beneficial adaptations and for which populations these benefits occur. Where possible, we identify specific signaling mechanisms through which heating a tissue or raising internal body temperature results in a multitude of beneficial adaptations. Lastly, this review also emphasizes those investigations that have shown little or no benefit of heat therapy. The overarching aim of this review was to provide scientists, clinicians, and the lay public with a current consensus on the benefits and limitations of heat therapy as a healthy lifestyle intervention for a variety of persons and health conditions.

## Graphical abstract

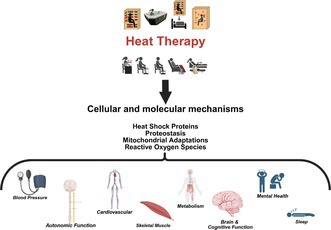

## 1 Introduction

Heat therapy is a historic modality that has been used for hundreds, if not thousands of years, as a source of lifestyle intervention and community for many different cultures (Laukkanen et al. 2015). Over the last ~40 years, it has gained increasing popularity among scientists and clinicians as a potential therapeutic tool for various populations and health conditions. Recently, several systematic reviews and meta‐analyses have sought to encompass specific aspects of the scientific literature surrounding this ancient therapeutic modality, each primarily focused on one beneficial aspect of heat therapy.

This review aimed to provide a more comprehensive evaluation of the current literature on heat therapy, including studies that demonstrate clear beneficial adaptations (and those that show no effect of heat therapy) on specific organs, crosstalk between different organs and tissues, and integrated physiological systems and pathways. We also propose that, based on current evidence, it is essential that we identify what forms of heat therapy confer beneficial adaptations and for which populations these benefits occur. Where possible, we identify specific signaling mechanisms through which heating a tissue or raising internal body temperature results in a multitude of beneficial adaptations. Lastly, this review has sought to emphasize when studies have shown little or no benefit. This review was framed to address the current available research and gaps in the current literature. We focused primarily on studies from human subjects but included studies utilizing animal models when human data were lacking and to better flush out the mechanistic pathways. Although scientific researchers were the main target audience for this review, we have endeavored to make the content accessible and practical for clinicians and public health practitioners as well. We aim to provide scientists, clinicians, and the lay public community with a current consensus on the benefits and limitations of heat therapy as a healthy lifestyle intervention.

## 2 Defining Heat Therapy

Given the various intentions for utilizing repetitive heat exposures (e.g., heat acclimation, acclimatization, heat events, and heat therapy), we propose revising the terminology used to describe various heat exposures for coherence and to define the scope of this review. Understanding and better defining chronic heat exposure terminology is also essential to disseminating the scientific results to the scientific community and the public.

We propose that the overarching goal of heat therapy or heat acclimation/acclimatization is to gain a heat‐adapted phenotype. This can be defined as the physiological and cellular adaptations an organism develops through the process of repetitive or chronic heat exposure. We wish to further define heat therapy as chronic, intermittent heat exposure *with the explicit intent to improve health and wellness, prevent disease, and promote healing*. We have elected not to place limits on this description based on the number of exposures or the time frame of heat therapy. However, the *F*requency, *T*emperature, *D*uration, and *M*odality of heat therapy, also known as the FTDM principle, must be considered when evaluating the effectiveness of heat therapy in generating adaptations. We are aware some in the field (Rodrigues et al. 2025) have proposed the use of the FITT (frequency, intensity, time, and type) principle, adopted from exercise programming, to describe a prescriptive method for heat therapy. We assert that the adoption of the FTDM principle phrasing allows for differentiation between exercise and heat therapy, in addition to providing a more specific prescriptive matrix that can ideally be used by clinicians.

Studies on how a single or limited number of heat exposures can impact health biomarkers may be vital to understanding the physiology of acute heat exposure or recovery from heat stress. They also assist in hypothesis generation (e.g., how acute heat exposure may translate into improved organ system or whole‐body health). However, such studies are not the focus of this review. Importantly, there is growing interest in whether an individual's response to a single heat exposure can predict their lasting benefit with chronic exposures (Romero et al. 2022). Although an intriguing concept, as seen in the data we present, minimal literature directly supports this treatise.

In contrast to heat therapy, we define heat acclimation (exposures in a laboratory environment) or heat acclimatization (exposures to a natural environment) as repeated exposures to heat to improve heat tolerance during exercise and work in hot conditions.

**FIGURE 1:**
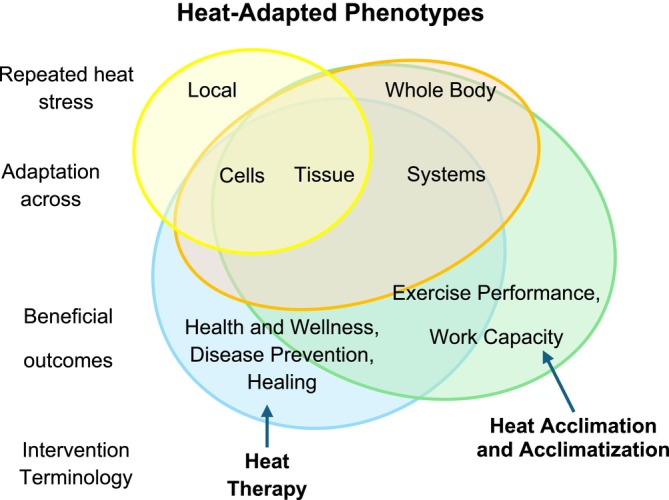
Overlapping concepts of the heat‐adapted phenotype. Repeated exposure to local heat stress elicits cellular adaptation, which can be observed at the tissue level, whereas whole‐body exposure results in adaptations at cellular, tissue, and systemic levels. These permutations of the heat‐adapted phenotype can be leveraged to promote health and wellness, prevent disease, and facilitate healing. Heat therapy involves the specific, repeated application of heat to achieve these beneficial outcomes, at levels determined by the extent of the heat stress (local vs. whole‐body). These permutations, when they encompass systems‐level adaptations from whole‐body exposure, can promote exercise performance and work capacity. Heat acclimation/acclimatization involves the specific, repeated application of whole‐body heat exposure to achieve these beneficial outcomes.

Both heat therapy and heat acclimation/acclimatization can be further divided into passive heat exposure (not accompanied by exercise) and active heat exposure (accompanied by exercise). There are relatively few studies that have investigated heat therapy with the addition of exercise. However, we predict (and hope) that there will be more research on this aspect. There are limited yet promising examples where exercise and heat exposure may confer more health or healing benefits to a given population than either modality alone. One example is for people with a spinal cord injury, who may not gain the full benefits of exercise training. Here again, there is a dearth of relevant research. Thus, most of the research discussed in this review, geared toward the target audience of scientific researchers, will be on passive heat therapy modalities. We have summarized each aspect of a heat‐adapted phenotype in Figure 1.

Lastly, it is crucial to recognize that a heat‐adapted phenotype, via heat therapy or heat acclimation/acclimatization, may confer protection from a climatic heat event, thereby preserving health and wellness or work capacity. Much heat therapy literature has focused on cardiovascular, autonomic, skeletal muscle, metabolic, mental, or cognitive health, not protection from climatic heat events. Few studies have explicitly examined how a heat‐adapted phenotype, gained from repetitive heat exposures that are beyond traditional heat acclimation protocols, confers heat protective benefits during a climatic heat event (see Rodrigues et al. 2024 for a thorough review of this topic). We are aware of ongoing studies investigating these topics. It is an important and exciting area of research, but it is beyond the scope of this review.

## 3 Modalities of Heat Therapy

**FIGURE 2:**
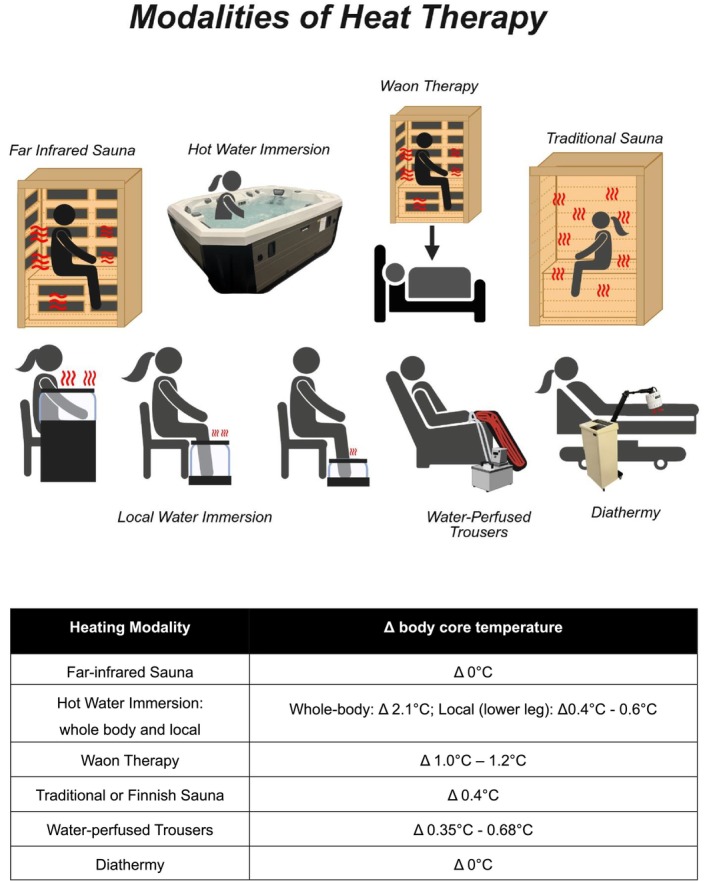
Each of the modalities highlighted in this figure has been discussed in detail in the modalities section of this review. To note, the local water immersion includes arm immersion, lower leg, and ankle/foot immersion. *Created with Biorender*.

The numerous modalities of heat therapy have deep cultural roots, having been implemented for centuries for health improvement and community gathering across many cultures (Laukkanen et al. 2015). In this section, we provide a brief overview of different heat therapy modalities, which can be visualized in Figure 2. Later in this review, we will discuss scientific investigations of the health benefits associated with each.

### 3.1 Finnish Sauna

Finnish sauna bathing is a cultural ritual utilized for thousands of years (Laukkanen and Kunutsor 2024). Finnish sauna is characterized by high temperatures of 80°C–100°C in dry, well‐ventilated environments (Heinonen & Laukkanen, 2018). Humidity is temporarily increased with water application to the hot rocks or heating elements inside the sauna, but on average remains below 20% (Laukkanen, Kunutsor, et al. 2018). Sessions can be interrupted for a cold exposure, such as a cold plunge, cold shower, or rest in a cool room, followed by a return to the heat exposure (Heinonen & Laukkanen, 2018). Frequency of participation in this ritual ranges from one to seven sessions per week, and the time (accumulated duration) of exposure varies from ~10 to 20 min per session (Laukkanen et al. 2015). However, there are many different variations in the protocols that individuals follow. As this is a cultural ritual in Finland, everyone from children to older adults participates, with societal norms reinforcing this lifelong habit and the human interactions generating additional health benefits (Heinonen & Laukkanen, 2018). As this practice has spread to other countries and cultures, there has been less of an emphasis on the cultural and community aspects of Finnish sauna bathing and more on relaxation or individual health benefits. Finnish‐style dry saunas are commonly installed in public and private gyms in the United States. Using the sauna after exercise has become a common practice in that setting.

Much of our knowledge regarding the cardiovascular health benefits of heat therapy comes from the Finnish Kuopio Ischemic Heart Disease Risk Factor Study, a prospective cohort study (Laukkanen et al. 2015). Laukkanen and colleagues prospectively analyzed 2315 middle‐aged men, using a self‐administered questionnaire at baseline (Laukkanen, Kunutsor, et al. 2018), with annual follow‐ups over a median length of nearly 30 years (Laukkanen et al. 2015). This work has led to a robust scientific understanding of the association of Finnish sauna bathing frequency and session time for many health conditions and outcomes (e.g., hypertension, vascular health, mental health) (Laukkanen et al. 2015, 2017; Kunutsor et al. 2018; Laukkanen and Laukkanen 2018; Laukkanen, Laukkanen, and Kunutsor 2018; Lee et al. 2018; Zaccardi et al. 2017). Although the initial cohort only included middle‐aged men, women were added 11 years into the study (Laukkanen, Kunutsor, et al. 2018).

From the initial cohort of men, they found that the cumulative hazard ratio for sudden cardiac death was lowest in individuals who participated in Finnish sauna sessions 4–7 times per week, for more than 19 min per session (Laukkanen et al. 2015). These findings were validated in a second cohort that included women, underscoring the efficacy of lifelong Finnish sauna use for promoting and maintaining cardiovascular health (Laukkanen, Kunutsor, et al. 2018). Subsequent investigations generalized the dose‐dependent responses to additional health outcomes (Laukkanen et al. 2015, 2017; Kunutsor et al. 2018; Laukkanen and Laukkanen 2018; Laukkanen, Laukkanen, and Kunutsor 2018; Lee et al. 2018; Zaccardi et al. 2017). One major limitation of this work is its translatability to other populations, given the commonality of the deep cultural roots in Finland and the human interactions specific to its practice there. Nonetheless, these are powerful and informative studies on heat therapy's health and longevity benefits when part of a lifestyle.

### 3.2 Onsen Bathing and Balneotherapy

In Japan, naturally occurring hot springs were developed into community‐oriented locations for hot water immersion known as *onsen* over 2000 years ago (Serbulea and Payyappallimana 2012). These became popular owing to the purported healing properties of the hot springs (Serbulea and Payyappallimana 2012). In 1948, the Hot Spring Law was passed, which defined an *onsen* as a natural spring that contained a specified amount of at least one of 19 predefined naturally occurring chemicals (later reduced to 9 types in 1979) with a maintained temperature of > 25°C (Serbulea and Payyappallimana 2012). Despite the widespread use of *onsen* bathing in Japan, few population‐based studies on its health benefits exist. One report found that individuals who engaged in *onsen* bathing have reduced arterial stiffness (Kohara et al. 2018). Another found that the risk of hypertension in women, as well as all‐cause cardiovascular disease risk in men, was reduced in individuals who regularly engaged in onsen bathing (Maeda et al. 2018). Serbulea and Payyappallimana (2012) have provided an informative review of Japan's history of *onsen* use.

Balneotherapy is a form of water immersion therapy similar to an *onsen*. Commonly used in health resorts, it has several monikers, depending on the substances in the water and the region (Verhagen et al. 2015). For example, it can be referred to as medical mineral waters when it contains peloids (a mixture of fine‐grained materials such as mud or clay) and natural gases such as carbon dioxide or hydrogen sulfide (Verhagen et al. 2015). Water temperature varies, but sometimes the thresholds < 35°C, 35°C–36°C, and > 36°C are used to specify thermal levels (Verhagen et al. 2015). While the data on health outcomes, detailed in a review of the effects of balneotherapy and rheumatoid arthritis, are largely equivocal (Verhagen et al. 2015), a report by Oyama and colleagues (Oyama et al. 2013) found improvements in clinical symptoms in chronic heart failure patients, following 2 weeks of daily 10‐min balneotherapy bathing in a hot spring with a temperature of 40°C (Oyama et al. 2013). Due to the large variance in frequency, temperature, and duration, not to mention the different types of balneotherapy baths, a consensus on adaptations or health improvements has yet to form.

### 3.3 Hot Tubs and Other Hot Water Immersion Modalities

In the United States, the use of hot tubs grew after the 1940s, inspired by Japan's cultural practices. The development of less expensive fiberglass shell hot tubs in the 1970s led to exponential growth in their availability and use (https://en.wikipedia.org/wiki/Hot_tub, n.d.). Hot water immersion has been a standard modality for heat therapy in clinical interventions in the United States for the past 25 years (Hooper, 1999). Water is an efficient heat conductor, and immersion in hot water effectively increases body core temperature (Brunt and Minson 2021). It has been employed in a variety of ways: from the shoulder down (Brunt, Eymann, et al. 2016; Brunt, Howard, et al. 2016), waist down (Thomas et al. 2017, 2016; Richey et al. 2022), lower leg (Romero et al. 2017; Cheng et al. 2021; Coombs et al. 2019), and ankle down (Cheng et al. 2021, 2024; Dong et al. 2010), sometimes varying conditions within a session. For example, Brunt and colleagues utilized hot water immersion from the shoulders down until the target core temperature was reached, then repositioned to waist‐down immersion to maintain temperature within a 90‐min session (Brunt, Eymann, et al. 2016; Brunt, Howard, et al. 2016). This modality of heat exposure has been studied extensively in several populations, investigating a variety of questions: heat adaptation (Fox et al. 1963a, 1963b), cardiovascular outcomes and molecular adaptations in physically inactive young adults (Brunt, Eymann, et al. 2016; Brunt, Howard, et al. 2016; Brunt et al. 2018), cardiovascular and metabolic outcomes in women with polycystic ovary syndrome (PCOS; Ely, Clayton, McCurdy, et al. 2019), metabolic outcomes in older adults at risk for Alzheimer's disease (AD; Blankenship et al. 2025), and others. The water temperature for these studies has ranged from 37°C to 41°C, with session times ranging between 30 and 90 min (Hooper, 1999; Brunt, Eymann, et al. 2016; Brunt, Howard, et al. 2016; Brunt et al. 2018; Ely, Clayton, McCurdy, et al. 2019; Blankenship et al. 2025; Ely, Clayton, et al. 2019). Most studies using this modality of heat therapy have been performed under laboratory supervision in a controlled laboratory space. We currently lack the rich data from population‐based studies on hot tub use that exists for Finnish sauna bathing.

In an attempt to adapt hot water immersion to a practical, deployable home‐use intervention, circulating water baths have been used to heat the lower legs (Romero et al. 2017; Richey, Hemingway, et al. 2024; Akins et al. 2024) or feet and ankles (Cheng et al. 2021, 2024). This heating modality increases body core temperature moderately compared to increases that occur with other whole‐body immersions (Romero et al. 2017; Cheng et al. 2024).

Water immersion in its many forms (*onsen*, balneotherapy, hot tubs) provides high rates of heat transfer but also imposes higher hydrostatic pressures than sauna or lower limb immersion. These hydrostatic pressures increase venous return (Brunt and Minson 2021), which should be considered when determining which heating modality to employ.

### 3.4 Far‐Infrared Sauna

Far‐infrared saunas have become increasingly popular in recent years, with the commercialization of exercise classes conducted in a room “heated” with far‐infrared and low‐cost home units coming on the market. The full far‐infrared range of the electromagnetic spectrum is between 3 and 1000 μm (Fox et al. 1963a), but the ceramic panels used as emitters in commercially available saunas are within a narrower range of 5–14 μm (Vatansever and Hamblin 2012; Qin et al. 2024). Far‐infrared saunas use a lower temperature setpoint (typically around 60°C–70°C) than traditional saunas, and once the setpoint has been reached, the far‐infrared emitters cycle on and off. Thus, entering the sauna while the unit is heating is recommended. Far‐infrared radiation is mainly absorbed by the molecular bonds of water in tissue, increasing the temperature (Vatansever and Hamblin 2012; Beever 2009). While it is widely suggested that the radiation emitted from the ceramic panels will penetrate 3–4 cm into the peripheral tissues (Heinonen & Laukkanen, 2018; Dong et al. 2010), a recent study investigated the depth of heat penetration within skeletal muscle using a commercially available far‐infrared sauna (Reed et al. 2025). In this study, the rise in muscle temperature was less with increasing depth into the tissue and was negligible beyond 3.8 cm below the skin surface. The thermic effect had lessened by 63% at a depth of 2.4 cm, which can be considered the effective thermal penetration. Notably, muscle temperature increased without changes in core temperature.

Atencio et al. 2025, compared the hemodynamic and thermoregulatory responses between far‐infrared sauna, traditional sauna, and hot water immersion. A 45‐min far‐infrared sauna session did not result in an appreciable increase in core temperature (as measured by an ingestible pill). Still, mean body temperature (calculated as (0.8 • mean core temperature) + (0.2 • mean skin temperature)) increased, and the participants lost some body mass through sweating (Atencio et al. 2025). It may be that far‐infrared radiation can raise the temperature around specific thermoreceptors within the spinal cord and skeletal muscles such that a thermoregulatory response is observable in the absence of changes in body core temperature.

Overall, the temperatures and duration of far‐infrared saunas have not been consistent across studies. Very few chronic exposure studies have utilized far infrared in humans. More studies have been conducted in cell cultures and animals (Kohara et al. 2018; Maeda et al. 2018; Verhagen et al. 2015; Oyama et al. 2013; https://en.wikipedia.org/wiki/Hot_tub, n.d.), but these do not translate well to humans. However, Fuchs et al. (2025) completed an 8‐week far‐infrared intervention in which young healthy participants were “acclimated” by starting heat exposures at 50°C for 35 min and progressing over the 8‐week intervention to 60°C for 45 min. Others have also used different lead‐in periods for heat therapy, which is important to consider depending on the population and the intensity of the modality being used.

### 3.5 Waon Therapy

Waon therapy also utilizes a far‐infrared sauna (Kihara et al. 2009; Kominami et al. 2020; Kuwahata et al. 2011; Miyata et al. 2008; Miyata and Tei 2010; Shinsato et al. 2010; Sobajima et al. 2015; Tei et al. 2007). This modality relies on 15 min in an infrared sauna at 60°C, immediately followed by a participant being covered in blankets for an additional 30 min of supine rest to maintain an elevated core temperature (Miyata and Tei 2010). While this is often referred to as a far‐infrared sauna modality in the literature, it should not be considered an isolated far‐infrared exposure, as the second portion of the therapy, which is not based on far‐infrared radiation, is believed to be critical to realizing the full benefit. The anticipated rise in core temperature is between 1.0°C and 2.0°C (Miyata and Tei 2010), whereas isolated far‐infrared may not increase core temperature.

Waon therapy was developed in 1989 as a therapeutic alternative to traditional sauna bathing for individuals with heart failure. The efficacy of this intervention for individuals with heart failure is evident (see the cardiovascular responses below). Indeed, during a retrospective study, individuals with heart failure were followed for 5 years (Kihara et al. 2009). Those who completed Waon therapy presented with a significantly lower incidence of rehospitalization due to heart failure or cardiac death compared to those who did not partake in Waon therapy (31.3% vs. 68.7%, respectively) (Kihara et al. 2009). Furthermore, Waon therapy is very successful in reducing cardiovascular comorbidities in several other patient populations (Kihara et al. 2009; Kominami et al. 2020; Kuwahata et al. 2011; Miyata and Tei 2010; Sobajima et al. 2015; Tei et al. 2007; Fujita et al. 2011), as we note later in this review.

### 3.6 Bikram Yoga

Bikram yoga is a style of *hatha* yoga in which practitioners complete 90 min of *hatha* yoga at a temperature of ~40°C with ~40% humidity (Choudhury 2007). When interventions combine physical activity and heat stress, it can be challenging to elucidate which outcomes result from exercise, in this case, *hatha* yoga, or heat exposure. An interaction of the two could also be driving the beneficial adaptations.

A discussion of the specific benefits of Bikram yoga will follow in later sections of this review, but collectively, it appears that regular physical activity performed in hot and humid conditions may improve health (Hunter, Dhindsa, Cunningham, Tarumi, et al. 2013; Hunter, Dhindsa, Cunningham, Hunter, et al. 2013; Hunter et al. 2017, 2016, 2023, 2018). Some cross‐sectional evidence suggests regular Bikram yoga practitioners have lower blood pressure than their age‐matched counterparts (Abel et al. 2012), and Bikram yoga appears to improve health when performed in individuals with disease (Hunter, Dhindsa, Cunningham, Tarumi, et al. 2013; Hewett et al. 2015). This modality has been shown to improve metabolic health (Hunter, Dhindsa, Cunningham, Tarumi, et al. 2013), and a number of investigations have focused on the effects of Bikram yoga on vascular health across the lifespan (Hunter, Dhindsa, Cunningham, Hunter, et al. 2013; Hunter et al. 2017, 2016, 2018).

### 3.7 Exercise and Heat Therapy

Combinations of exercise and heat exposure have been employed in heat acclimation protocols for those seeking to improve athletic performance or work capacity, but less frequently as heat therapy to promote health improvements. Hence, there are limited data on the combined effects of exercise and heat therapy, and more targeted investigations on exercise and heat therapy interventions in populations with poor health or disease are warranted.

Acutely, combined aerobic exercise and sauna use have been reported to decrease blood pressure immediately following and 24 h after the exposure (Rissanen et al. 2020). A combination of exercise and traditional sauna has been shown to reduce systolic blood pressure, total cholesterol, and increase cardiorespiratory fitness compared to either exercise or sauna alone (Lee et al. 2022). Akerman et al. (2019) investigated whether heat therapy and calisthenics combined could improve health in patients with peripheral artery disease and reported that there were improvements in both blood pressure and walking distance that were comparable to their exercise‐alone intervention. Gayda, Bosquet, et al. (2012) examined the effect of exercise followed by sauna in adults with untreated hypertension compared to sauna alone and found that it also improved systolic blood pressure.

### 3.8 Pulsed Short‐Wave Diathermy

Pulsed short‐wave diathermy delivers high‐frequency electromagnetic waves to heat tissue and has been widely used in tissue and joint recovery (Goats 1989a, 1989b; Draper et al. 2013, 1999) by physical therapists and athletic trainers. Recently, researchers have used it to study localized heat therapy (Kaluhiokalani et al. 2025; Marchant et al. 2022; Hafen et al. 2019, 2018; Hyldahl et al. 2021). Unlike infrared sauna, short‐wave diathermy penetrates and heats tissue deeper than that of far infrared (Goats 1989a, 1989b). Another advantage of short‐wave diathermy systems is their ability to deliver either a continuous or a pulsed output of heat stimulus (Goats 1989a). A shorter pulsed output allows the tissue time for “recovery” from the stimulus between each pulse, but with higher pulse frequencies and durations, the recovery time is shortened, making the stimulus constant (Goats 1989b). Draper and colleagues (Draper et al. 2013) reported that when they used a pulsed short‐wave diathermy (800 pulses per second and a pulse width of 400 μs) the temperature in the *triceps surae* muscle increased ~4°C (Draper et al. 2013). This increase in muscle temperature is indicative of the adjustment in the tissue to the heat stimulus (Draper et al. 2013). This large increase in tissue temperature defines the diathermy as a “vigorous” heating implement capable of deep thermotherapy, reaching a depth of 3–4 cm (Draper et al. 1999). Despite the substantial increase in muscle temperature and the increase in skin temperature, this modality does not increase body core temperature (Draper et al. 1999) making it the ideal piece of equipment to isolate specific tissue regions and heat over longer treatment durations.

### 3.9 Water‐Perfused Pants

Water‐perfused suits were initially designed for whole‐body cooling of astronauts while wearing the Apollo space suit (Brengelmann et al. 1977). Researchers have since adapted water‐perfused suits for research on thermoregulation and environmental physiology, as close spacing of the tubing that lines the inside of the suit provides for effective heat exchange with the participant (Brengelmann et al. 1977).

Thus, the suits have been used as a method of whole‐body temperature manipulation, but recently, partial suits (primarily the pants) have been deployed as a means of heat therapy for aged adults (Ruiz‐Pick et al. 2025) and individuals with peripheral artery disease (Monroe et al. 2020, 2022). Ruiz‐Pick et al. 2025 employed this model for aged adults to determine whether leg vascular function would improve following 8 weeks of home‐based heating. Participants were randomized into two groups: a control group (31°C circulating water) and a heat therapy group (51°C circulating water) (Ruiz‐Pick et al. 2025). They were asked to perform the control or heat therapy for 4 days per week for 60 min each session (Ruiz‐Pick et al. 2025). Monroe and colleagues also used this method of intervention in their participants with peripheral artery disease (Monroe et al. 2020, 2022). For their home‐based intervention, they had their heat group use the pants 7 days per week, 90 min per session at a circulating water temperature of 43°C (Monroe et al. 2022). Prior to this intervention, they also used this heating method in the laboratory, where participants were asked to come in for 6 weeks and perform either heating or sham 3 days per week for a total of 18 sessions (Monroe et al. 2020). The water temperature for the in‐laboratory visits was 48°C and 33°C for the heat and sham, respectively (Monroe et al. 2020). Interestingly, body core temperature only marginally increased, with elevations of Δ 0.3–0.4 between the two studies (Monroe et al. 2020, 2022).

### 3.10 Potential Risks of Passive Heat Therapy

There are several potential health and safety risks associated with any form of heat therapy. These include the risk of overheating (including heat exhaustion or heat stroke), dehydration, hypotension, and/or orthostatic intolerance, as well as the risk of burns on hot surfaces. One study cited that the main causes for injuries during sauna bathing were falls and reports of syncope (Kaiser et al. 2023). Slips and falls also accounted for the greatest number of injuries in hot tubs or spas in the United States between 1990 and 2007 (Alhajj et al. 2009). Skin burns are also a potential risk, particularly for sauna use, in which direct contact with hot surfaces is possible. There is also a possibility that exceptionally hot water may also cause burns, particularly at natural hot springs where the water temperature is not controlled. Some clinical conditions, such as diabetes, peripheral neuropathies, and those with spinal cord injuries, may have the additional risk of burns on hot surfaces as they may have decreased temperature or pain sensitivity, or the integrity of the skin and the physiological responses to dissipate direct heat may be compromised.

Most of the heat‐related risks are mitigated by following standard practices for each modality and by limiting the exposure temperature and duration. Particularly for new or naïve practitioners, it is imperative to monitor thermal perception closely. Although most people do not measure body temperature while undergoing heat therapy, limiting exposure to a 2°C core temperature rise will minimize the heat‐related risks. A thermal perception of feeling “hot” would be acceptable, but increasing thermal perception to feeling “very hot” or “uncomfortably hot” would increase heat‐related risks. As with any activity, there are additional risks for people with underlying disease conditions (such as cardiovascular disease, diabetes, multiple sclerosis, and kidney disease), those with reduced sweat production, such as the very young and the elderly, and for those with spinal cord injury. Some clinical conditions, such as diabetes, peripheral neuropathies, and those with spinal cord injuries, may have the additional risk of skin burns on hot surfaces as they may have decreased temperature or pain sensitivity, or the integrity of the skin and the physiological responses to dissipate direct heat may be compromised.

In hot tubs and for those who service them, there have been reports of “hot tub lung,” a form of hypersensitivity pneumonitis where aerosolized water that is contaminated with *mycobacterium avium* complex is inhaled. Reports of these conditions are quite rare (and are mostly associated with poor maintenance of the facility, Yasin et al. 2017).

There are very few studies on miscarriage in women undergoing planned heat exposure, and the incidence is extremely low (Li et al. 2003). Early in pregnancy, there is a concern that exposure to higher temperatures could increase the risk of neural tube defects (Chambers 2006). Again, the increased risk with planned heat exposure is extremely low when general guidelines for safe use are practiced. The risk of neural tube defects for a typical pregnancy is about 1 in 1000, with some studies suggesting the risk doubles to 2 in 1000 with exposure to higher temperatures, such as hot tub water (Milunsky et al. 1992). That said, additional care should be taken to limit the frequency, temperature, and duration. For men, there have been reports of decreased sperm motility following heat exposure (hot tub) which may temporarily decrease the chance of conception (Shefi et al. 2007).

Alcohol use greatly increases the risk of minor injuries as well as more serious injuries or death, although there are no comprehensive data on this. In Finland, it is reported that alcohol use is a contributing factor to the reported 20–25 sauna‐related deaths per year (Ylikahri et al. 1988). Simply put, there are benefits to heat therapy, but intelligent decision‐making and caution should be used to avoid the detrimental risks associated with the various modalities.

### 3.11 Modality Comparisons

As we have described, there are several modalities that can be used in different ways for heat therapy. In the following sections, we will discuss specific adaptations that have been reported in various physiological processes. Comparing the effectiveness of heat therapy modalities is subjective, as to our knowledge no group has performed a heat therapy study comparing multiple modalities. Atencio et al. (2025) compared the acute differences between hot water immersion, traditional sauna, and far‐infrared sauna. Based on their results, hot water immersion elicits the greatest acute cardiovascular and immune responses, but we fully acknowledge the limits of extrapolating from acute studies to chronic implementation as a therapy. Another consideration when comparing and choosing a heat therapy modality should be the overarching goal of the heat therapy. If localized improvements are the goal, then pulsed short‐wave diathermy would be the best option. Tables 1, 2, 3 provide a comprehensive assessment of various modalities and their associated beneficial or null outcomes.

**TABLE 1:** Heat therapy studies in humans.

Frequency	Duration	Temperature	Length of treatment	Participant population	Benefits: cardiovascular, metabolic, performance, mental health, etc.	References
*Whole‐body heat therapy*						
**Hot water immersion**						
6 days/week	30 min	37.8°C–41.0°C	3 weeks	Adults (43–68 years) Type 2 diabetes	Metabolic	Hooper (1999)
4–5 days/week	90 min	40.5°C	8 weeks	Adults (18–30 years) Physically inactive	Cardiovascular	Brunt, Eymann, et al. (2016) and Brunt, Howard, et al. (2016)
3–4 days/week	60 min	40.5°C	8–10 weeks	Adult women with PCOS (23–31 years)	Metabolic and Cardiovascular	Ely, Clayton, et al. (2019) and Elay, Francisco, et al. (2019)
3 days/week	30 min	42°C	8 weeks	Adult women (25 ± 5 years)	Cardiovascular	Bailey et al. (2016)^a^
8–10 sessions	60 min	40°C	2 weeks	Adults with T2D (65 ± 8 years)	Cardiovascular	James et al. (2023)
3–4 days/week	45 min	40°C	8–10 weeks	Adults with stage 1 Hypertension (48 [45, 51] years)	None	Kaiser et al. (2025)^a^
10 sessions/14 days	45–60 min	38.5°C–39.0°C	2 weeks	Overweight men (33 ± 10 years; BMI = 31 ± 4 kg/m^2^)	Metabolic	Hoekstra et al. (2018)
3 days/week	45 min	Body core temperature Δ 1°C	4 weeks	Adults with metabolic risk (Age > 65 years)	No change in metabolic	Blankenship et al. (2025)
3–4 days/week	60 min	40.5°C	8–10 weeks	Adults (57–76 years)	Reduced blood pressure	Brunt et al. (2019)
3 days/week	45 min	40.5°C	4 weeks	Adults (45 ± 8 years)	Improved fibromyalgia symptoms, decreased HSP90 and increased HSP40 and HSP72	Chadwick et al. (2025)
1–4+ days/week	16 ± 14 min	Not reported	Ongoing	Adults with T2D (70 ± 14 years)	Metabolic	Katsuyama et al. (2022)
7 days/week	20 min	39°C	4 weeks	Adults with T2D and lower extremity stenosis (mild, 61 ± 9 years; moderate, 63 ± 9 years; severe, 65 ± 9 years)	No change in metabolic	Qiu et al. (2014)
5 days/week	30 min	39°C–41°C	4 weeks	Adults (59 ± 2 years)	Δ in operating point	Cui et al. (2002a, 2002b)
6 ± 2 sessions/week	12 ± 10 min	25°C–45°C	Ongoing	Adults (65 ± 9 years)	Cardiovascular	Kohara et al. (2018)
**Balneotherapy/Onsen**						
5 days/week	30 min	38°C	12 weeks	Adults with T2D (35–75 years)	?	Sebők et al. (2022)
7 days/week	30 min	38°C	3 weeks	Adults with obesity (BMI > 25 kg/m^2^)	Metabolic	Oláh et al. (2011)
5 days/week	20 min	41°C	3 weeks	Women with obesity with and without T2D (58 ± 10 years)	Metabolic	Koçak et al. (2020)
< 1×/month to ≥ 4–5×/week	< 10 min to ≥ 40 min	25°C–45°C	Ongoing	Adults (65 to ≥ 85 years)	Cardiovascular	Maeda et al. (2018)
**Waon therapy**						
5 days/week	45 min	60°C (FIR)	10 weeks	Adults with PAD (74 ± 7 years)	Cardiovascular functional capacity	Tei et al. (2007)
5 days/week	45 min	60°C (FIR)	2 weeks	Adults with CHF (26–94 years)	Cardiovascular	Miyata et al. (2008)
7 days/week	45 min	60°C (FIR)	2 weeks	Adults with (38 ± 7 years) and without (35 ± 8 years) coronary risk factors	Cardiovascular and blood pressure	Imamura et al. (2001)
7 days/week	45 min	60°C (FIR)	2 weeks	Adults (43 ± 17 years) with coronary risk factors	ROS and blood pressure No change in metabolic	Masuda et al. (2004)
5 days/week	45 min	60°C (FIR)	2 weeks	Adults (62 ± 15 years) with CHF	Blood pressure	Kihara et al. (2002)
5 days/week	45 min	60°C (FIR)	4 weeks	Adults (63 ± 15 years) with CHF	Autonomic function and blood pressure	Kuwahata et al. (2011)
5 days/week	45 min	60°C (FIR)	4 weeks	Adults (28 ± 15 years) with chronic fatigue	Chronic fatigue and anxiety and depression	Soejima et al. (2015)
5 days/week	45 min	60°C (FIR)	4 weeks	Adults (38 ± 15 years)	Somatic and mental complaints	Masuda et al. (2005)
7 days/week	45 min	60°C (FIR)	2 weeks	Adults with obesity	Metabolic	Biro et al. (2003)
5 days/week	45 min	60°C (FIR)	3 weeks	Adults with CHF (68 ± 14 years)	Exercise tolerance and endothelial function	Ohori et al. (2012)
**Finnish sauna**						
4 days/week	20–30 min	79°C, 13% humidity	8 weeks	Adults with stable CAD (56–68 years)	None	Debray et al. (2023)
1–7 days/week	Varied	80°C–100°C	Ongoing	Adult men (42–60 years)	Blood pressure	Zaccardi et al. (2017)
1–7 days/week	Varied	80°C–100°C	Ongoing	Adult men (42–61 years)	Psychotic disorders	Laukkanen et al. (2018), Laukkanen et al. (2018)
1–7 days/week	Varied	80°C–100°C	Ongoing	Adult men (53 ± 5 years)	Cardiovascular all‐cause mortality	Laukkanen et al. (2017)
3 days/week	30 min total	80°C–90°C	6 weeks	Adults (26 ± 3 years)	Autonomic function (HRV)	Kunbootsri et al. (2013)
**Far‐infrared sauna**						
4–8 sessions	110–140 min	57.2°C	1 week	Adults (42 ± 13 years)	Depression symptoms	Mason et al. (2024)
3 days/week	35–45 min	50°C–60°C	8 weeks	Adults (65–85 years)	Muscle capillarization	Fuchs et al. (2025)
3 days/week	40–50 min	40°C; 40% relative humidity	6 weeks	Healthy adults (21 ± 1 years)	Metabolic	Hesketh et al. (2019)^a^
**Environmental chamber**						
4–5 days/week	60 min	48°C–50°C; 50% relative humidity	2 weeks	Adult men (34 ± 3 years)	Reduced atrophy, maintained muscle strength	Labidi et al. (2024)
7 days/week	60 min	48°C–50°C; 50% relative humidity	11 days	Adult men (33 ± 8 years)	Improved skeletal muscle contractility	Racinais et al. (2017)

Abbreviations: Δ, change; CAD, coronary artery disease; CHF, chronic heart failure; HRV, heart rate variability; HSP, heat shock protein; PAD, peripheral artery disease; ROS, reactive oxygen species; T2D, type 2 diabetes.

^a^ Compared exercise intervention.

**TABLE 2:** Local heat therapy studies in humans.

Frequency	Duration	Temperature	Length of treatment	Participant population	Benefits: cardiovascular, metabolic, performance, mental health, etc.	References
*Local heat therapy*						
**Diathermy**						
2 h/day	2 h	—	6 days	Adults (20–22 years)	Skeletal muscle mitochondrial function	Hafen et al. (2018)^a^
2 h/day	2 h	—	10 days	Adults (18–39 years)	Mitigation of immobilization effects	Hydahl et al. (2021)^a^, ^b^
3 days/week	2 h	Muscle temperature reached 39.5°C	6 weeks	Adults (20–25 years)	Skeletal muscle metabolic and mitochondrial function	Marchant et al. (2022)^a^
3 days/week	2 h	Muscle Temperature increase Δ3.2 ± 0.3°C	6 weeks	Adults (18–36 years)	Resistance artery function	Kaluhiokalani et al. (2025)^a^
**Alternative heating modalities**						
4 days/week	8 h	Muscle Temperature reached 38.3°C ± 0.1°C	10 weeks	Adult men (45 ± 2 years)	Increase in transcript level of genes, specifically those associated with ATP synthesis	Goto et al. (2011)
3 days/week	30 min	~37°C–40°C	3 weeks	Adults (26 ± 3 years)	Effective for treatment of acute sport skeletal muscle injuries	Giombini et al. (2001)
45 min/day	45 min	42°C	10 days	Adults (18–29 years)	None	Francisco et al. (2017)
3 days/week	30 min	42°C	8 weeks	Adult men (22 ± 2 years)	Cutaneous vascular function	Green et al. (2010)
3 days/week	30 min	Study one: lower body 40°C, arms 30°C; Study two: lower body 40°C, arms not immersed	8 weeks	Adult men (24 ± 3, study one; 26 ± 3, study 2 years)	Cutaneous vasodilation	Carter et al. (2014)
3 days/week	45 min	42.8°C	8 weeks	Young adults (18–35 years)	Arterial stiffness, cardiorespiratory fitness, and performance	Cheng et al. (2024)
4 days/week	45 min	42°C	8 weeks	Postmenopausal women with hypertension (69 ± 5 years)	None	Richey et al. (2025)
**Water‐perfused trousers**						
3 days/week	90 min	48°C	6 weeks	Adults with symptomatic PAD (61–77 years)	Perceived physical function	Monroe et al. (2020)
7 days/week	90 min	43°C	8 weeks	Adults with PAD (40–80 years)	Functional capacity and walking performance	Monroe et al. (2022)
5 days/week	90 min	52°C	8 weeks	Adults (24 ± 5 years)	Heat shock proteins, proangiogenic environment	Kim, Monroe, et al. (2020), Kim, Reid, et al. (2020)
4 days/week	60 min	51°C	8 weeks	Adults (67 ± 7 years)	Blood pressure and cardiovascular	Ruiz‐Pick et al. (2025)
3 days/week	30 min	40°C	8 weeks	Adult males (26 ± 3 years)	Vascular	Carter et al. (2014)

Abbreviations: Δ, change; nPAD, peripheral artery disease.

^a^ Compared exercise intervention.

^b^ 400 and 800 pulses for diathermy.

**TABLE 3:** Heat therapy and exercise studies in humans.

Frequency	Duration	Temperature	Length of treatment (weeks)	Participant population	Benefits: Cardiovascular, Metabolic, performance, mental health, etc.	References
*Heat therapy +* e*xercise*						
3 days/week	90 min	35°C–41°C; 60% Relative Humidity	8	Young adults (29 ± 6 years)	Flexibility and body composition	Tracy et al. (2013)
3 days/week	90 min	40.5°C; 40%–60% Relative humidity	8	Young (18–39 years) Middle‐aged to older adults (40–70 years)	No Δ in young adults, improved endothelial dysfunction in middle aged and older adults	Hunter et al. (2017)
5 days/week	45 min	52°C	4	Black women (31 ± 8 years)	Vascular and blood pressure	Hunter et al. (2023)
3 days/week	45 min of Exercise +45 min of Lower leg Heat	Exercise: normothermic heat therapy: 42.8°C	8	Young adults (18–35 years)	Arterial stiffness, cardiorespiratory fitness, and performance	Cheng et al. (2024)
3–5 days/week	20–30 min (heating) + 30 min Calisthenics	39.5°C	12	Older adults with PAD (76 ± 8 years)	Blood pressure and functional capacity	Akerman et al. (2019)^a^
3 days/week	20–30 min heating +15 min resistance exercise	~40°C	12	Older adults with osteoarthritis (66 ± 7 years)	Lowered blood pressure	Roxburgh et al. (2023)^a^

Abbreviations: Δ, change; PAD, peripheral artery disease.

^a^ Compared exercise intervention.

When we consider the “safest” heat therapy modality, we must first consider the accessibility of heat therapy modalities. For example, the general population does not usually have access to water perfused pants, but they do have greater access to a sauna or hot tub. Therefore, the incidence of injury is going to have a selection bias for those modalities that are more accessible. The authors of this manuscript advise all heat therapy users to always follow safety recommendations for any modality they employ.

## 4 Does Heat Therapy Cause Long‐Term Adaptations in the Human Body?

**FIGURE 3:**
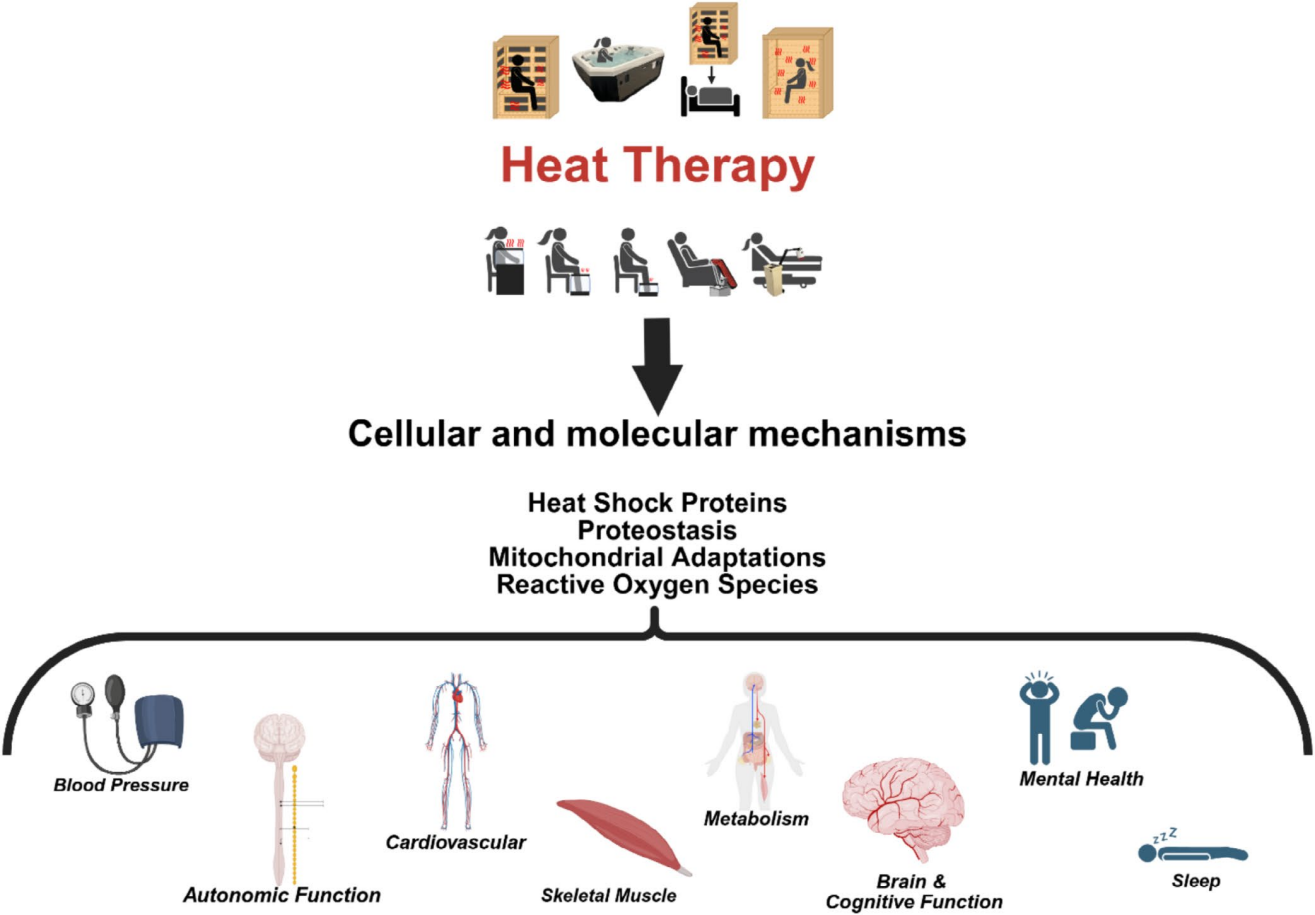
Summary figure of the discussion points within this review. The predominance of evidence suggests heat therapy has an impact on all aspects of health and performance, but the drivers of that improvement, the extent to which they are improved, and the specific populations in which improvements manifest are far from completely understood. We will use this review to describe the known and unknown effects of heat therapy and the interorgan crosstalk between various organs and systems. *Created with Biorender*.

Our goal with this section is to describe whether heat therapy elicits long‐term adaptations in humans. We will discuss the potential cellular and molecular mechanisms that may drive responses in blood pressure, autonomic function, cardiovascular health and function, skeletal muscle health and function, metabolism, brain and cognitive function, sleep, and mental health as summarized in Figure 3. We will review and discuss the research published in each area and identify gaps in the research. By clearly demonstrating the adaptations, or lack thereof, we will be able to delineate which modalities elicit beneficial adaptations and what populations and conditions benefit the most or not at all. However, as noted above, not every modality of heat therapy will increase temperatures in all regions or tissues. This is a key aspect of the interpretation for each study we will discuss.

## 5 Cellular and Molecular Mechanisms: Potential Drivers of Adaptations Following Heat Therapy

Adaptations to heat therapy occur in many organ systems. These adaptations include several common cellular and molecular pathways that drive these changes. There is also substantial interorgan communication by which adaptations in numerous tissues are simultaneously affected by heat therapy. This is, in many ways, similar to exercise, in which every cell in the body can undergo a hormetic stress when whole‐body or core temperature is elevated. Multiple inflammatory, hormonal, and blood‐borne signals that are often released from specific tissues, such as skeletal muscle, may work to allow communication between the organ systems. In the section below, we review numerous key substances and pathways that are activated by heat exposure. Much of the data is from studies following acute heating in both animal and human models, but we have worked to place these studies in the context of humans and chronic heat therapy. While this list is not exhaustive, it does highlight the most common mechanisms that are investigated in heat therapy research and identifies areas where questions remain.

### 5.1 Heat Shock Proteins

**FIGURE 4:**
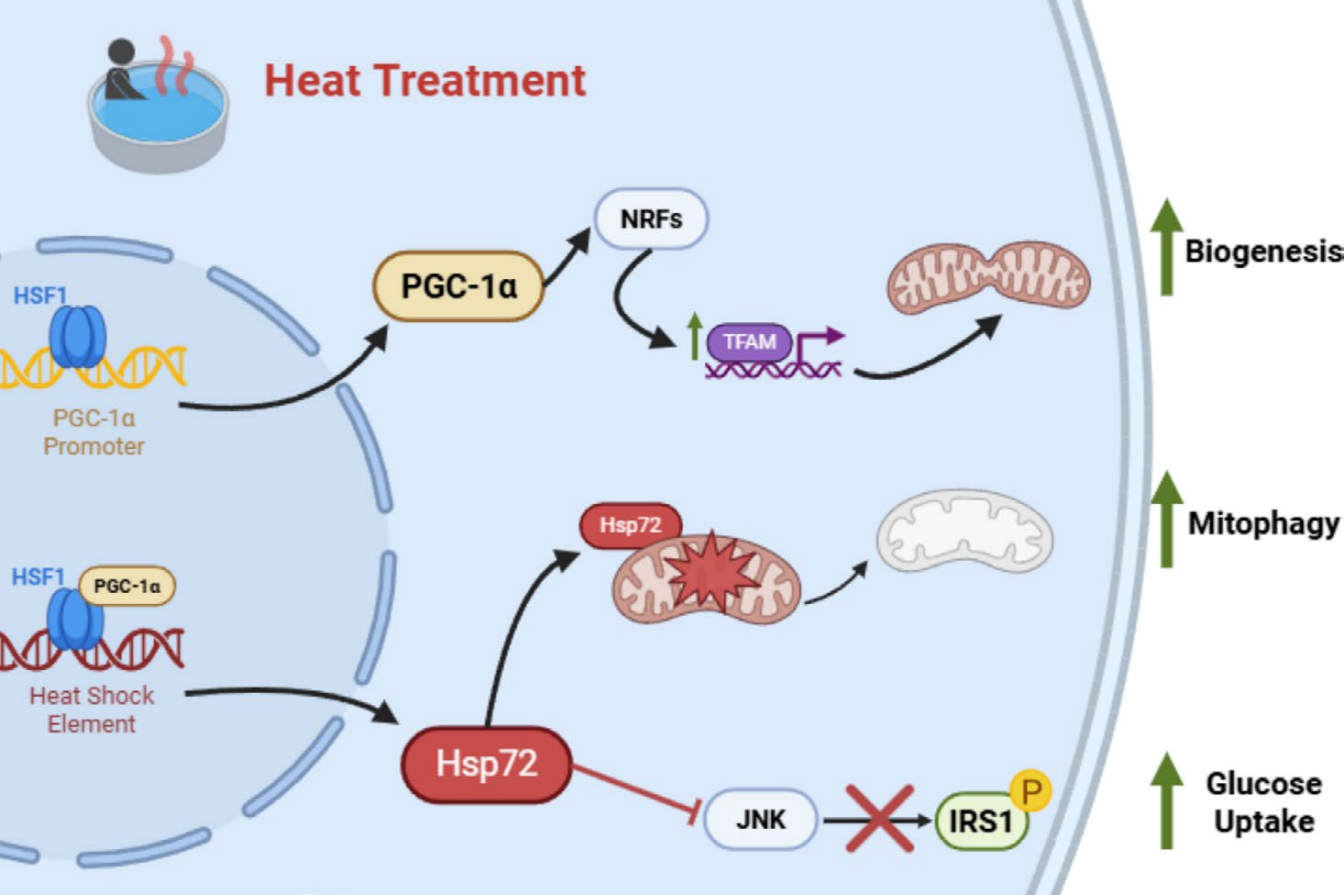
Molecular mechanisms of heat shock proteins (HSP) to improve metabolic health. This schematic represents both acute and chronic effects of heat treatment on cellular metabolic pathways. Heat activation results in translocation of the major heat shock transcription factor, HSF1, to the nucleus where it trimerizes, binds to DNA and is phosphorylated resulting in transcription of a family of heat shock proteins as well as of PGC‐1a. HSF1 binds to the promoter of PGC‐1a and regulates its gene expression. PGC‐1a acts as a master regulator by activating transcription factors like NRFs and TFAM, which ultimately lead to increased mitochondrial biogenesis. Increased expression of HSPs as a result of HSF1 activation can have numerous effects. Activation of HSP72 in liver and skeletal muscle has been shown to result in a degradation of damaged mitochondria through the process of mitophagy. In addition, HSP72 has been shown to bind directly to the stress kinase JNK and inhibit JNK‐mediated IRS1 serine phosphorylation. This JNK inhibition can result in improved insulin signaling and increased glucose uptake. The combined effect of these metabolic adaptations is an improvement in cellular metabolism and whole‐body glucose homeostasis following chronic heat treatment. HSF1, heat shock factor 1; PGC‐1a, peroxisome proliferator‐activated receptor‐g coactivator 1a; NRFs, nuclear regulatory factors; TFAM, mitochondrial transcription factor A; HSP72, heat shock protein 72; JNK, c‐Jun N‐terminal kinase; IRS1, insulin receptor substrate 1; P, phosphorylation. *Created with Biorender.*

Heat shock proteins (HSPs) were originally discovered in response to heat stress in Drosophila cells (Ritossa 1962; Tissiéres et al. 1974) and are currently thought to be primary mediators of the cellular metabolic response to heat therapy. This family of stress proteins and chaperones is expressed by all cells throughout the body (Kregel 2002) and functions to maintain normal protein folding and protect against cellular stress (Feder and Hofmann 1999). Each HSP is classified according to its molecular weight in kilodaltons, with the most studied families being HSP60, HSP70, and HSP90 (Hu et al. 2022). While some HSPs are expressed constitutively, HSP70 (also known as HSPA1 or HSP72) is induced by changes in pH, shear stress, and metabolic stress resulting from exercise and heat. The induction of HSPs in response to those stimuli is known as the heat shock response and is under the control of the highly conserved transcription factor, heat shock factor 1 (HSF‐1). Upon the initiation of the stress stimuli, HSF‐1 trimerizes and translocates to the nucleus, where it binds to promoters containing heat shock elements and subsequently induces stress‐responsive transcriptional targets, including HSPs (Kline and Morimoto 1997). HSPs are thought to have a variety of roles in metabolic tissues, including decreasing inflammation, improving mitochondrial function/oxidative capacity, and maintaining proteostasis, as discussed in greater detail in this review. Figure 4 summarizes the impact of heat treatment on HSPs and the subsequent downstream effects on metabolic health as described in this section.

#### 5.1.1 The Role of Stress‐Inducible HSP70/72 in Health and Disease

Perhaps because of its inducible nature, HSP70/72 has been extensively studied in preclinical and cellular models of ischemia–reperfusion injury. Early studies investigated the cytoprotection and quantification of HSP70 in an animal model of whole‐body acute heat exposure (Currie et al. 1988). They reported that whole‐body heat exposure‐induced HSP70 within cardiomyocytes, which in turn improved muscle function and reduced markers of muscle damage following ischemia–reperfusion injury (Currie et al. 1988). This finding was supported by other work that reported acute heat exposure increased HSP70 mRNA (Currie and Tanguay 1991) and improved cardiomyocyte viability following ischemia–reperfusion insult (Yellon et al. 1992; Donnelly et al. 1992; Gowda et al. 1998). Conversely, others have reported that prior acute heat exposure does not confer protection against ischemia–reperfusion injury (Saganek et al. 1997; Xi et al. 1998; Lille et al. 1999). One explanation is that the protective benefits of acute heat exposure are due to redundant response mechanisms. One such causal pathway is the upregulation of catalase or manganese superoxide dismutase, both potent antioxidative proteins, that induces HSP70 to provide cytoprotection in response to ischemia–reperfusion insult (Currie and Tanguay 1991; Karmazyn et al. 1990; Yamashita et al. 1997). HSP70 has also been shown to exhibit similar protective mechanisms against vascular smooth muscle cell hypertrophy induced by angiotensin II (Zheng et al. 2006).

HSP70 expression, induction, and function have been widely studied in skeletal muscle, which is the largest “organ” in the body and is likely a source of molecular signals involved in interorgan communication aligned with heat therapy. Skeletal muscle is the primary tissue responsible for insulin‐stimulated glucose uptake (Katz et al. 1983) and changes in skeletal muscle HSP expression have been examined in obese, insulin‐resistant, and Type 2 diabetic individuals. Skeletal muscle HSP72 expression is inversely related to body fat percentage and blood glucose in healthy participants (Henstridge et al. 2010; Kavanagh et al. 2016). Similarly, HSP72 levels are reduced in the skeletal muscle of individuals with Type 2 diabetes (Bruce et al. 2003; Kurucz et al. 2002; Rodrigues‐Krause et al. 2012), obesity, and insulin resistance (Chung et al. 2008; de Matos et al. 2014). As a result, HSP72 expression levels seem closely correlated with adiposity, and reduced expression correlates with the progression from obesity to metabolic disease. As discussed below, conditions or tissues in which HSP expression is diminished, as in the case of obesity or diabetes, may set the stage for significant physiological and clinical improvements through heat therapy.

A number of human studies have examined extracellular HSP expression, primarily in either serum or plasma, in response to passive heating. Faulkner and colleagues demonstrated that 1 h of hot water immersion (water temperature at 40°C) increased plasma concentration of HSP72 in healthy men (Faulkner et al. 2017). Similarly, Iguchi and colleagues reported increased plasma HSP72 concentration in healthy men and women in response to acute heat exposure for 30 min in a 73°C room (Iguchi et al. 2012). Several studies have also reported an increase in cell‐specific expression of HSP72. Brunt et al. showed that 1 h of hot water immersion (water temperature of 40.5°C) increases peripheral blood mononuclear cell HSP72 levels in healthy inactive men and women (Brunt et al. 2018). During a longer duration heat stimulus of 2 h of hot water immersion (water temperature of 39.5°C), HSP72 levels in monocytes were increased in healthy men and women (Oehler et al. 2001).

While studies suggest the source of extracellular HSP72 is likely skeletal muscle, few human studies have measured expression of this protein in skeletal muscle following heat treatment. Given its well‐established role in skeletal muscle function and metabolic signaling, this is an area that needs more research. The lack of extensive research in this area could be due to the difficulty of obtaining human biopsy samples and/or the challenge of sample timing postheat stress. Morton and colleagues reported no change in skeletal muscle HSP72 protein content 48 h following 1 h unilateral leg water immersion protocol (water temperature of 45°C) despite achieving substantial increases in intramuscular temperature (~3.6°C) (Morton et al. 2007). Single‐leg heating was also employed by Hafen and colleagues using pulsed short‐wave diathermy (Hafen et al. 2018). Their results were similar to those of Morton et al., with no reported increases in skeletal muscle HSP72 protein content immediately following 2 h of diathermy (Hafen et al. 2018). It is possible that the narrow biopsy timing in these studies (immediately post vs. 48 h post) did not align with the transient peak in inducible HSP72 expression, which has a relatively short half‐life and may return toward baseline within hours after the thermal stimulus. Additionally, it is possible that the single‐leg heating models do not provide a sufficient heating stimulus to induce an HSP response; we do not know the minimum temperature or duration of exposure that is required to observe an increase in HSP72 in skeletal muscle.

The HSP72 response to chronic heat exposure varies depending on study parameters, participant characteristics, and the type of HSP measured. James et al. (2023) reported no effect of 8–10 hot water immersion sessions over 14 days on extracellular HSP70 expression in individuals with type 2 diabetes. This agreed with another study that examined 8 weeks of hot water immersion in healthy men and women (Brunt et al. 2018). In contrast, 6 days of diathermy for 2 h per day, a modality that significantly increases muscle temperature, increased skeletal muscle HSP72 (Hafen et al. 2018). Interestingly, Hoekstra and colleagues found a reduction in extracellular HSP72 following 2 weeks of hot water immersion (Hoekstra et al. 2018), while Brunt and colleagues found an increase in HSP72 in peripheral blood mononuclear cells following 8 weeks of hot water immersion (Brunt et al. 2018). Cheng et al. (2021) examined the minimum effective dose of lower limb heating needed to elicit acute changes in upper limb micro‐ and macrovascular health, as well as circulating levels of HSP72.

Maloyan et al. (1999) reported that heat acclimation resulted in a greater expression and concentration of HSP72 and more readily inducible HSP72 gene transcription. These adaptations condition the organism to subsequent heat exposure. Moreover, the resilience to subsequent heat exposure is correlated with the length of heat acclimation. For example, the authors compared the HSP response after 1, 2, and 30 days of consecutive heat exposure (Maloyan et al. 1999). Notably, following the longest subsequent heat exposure, a greater exposure length was required to induce a comparable or greater HSP72 response (Maloyan et al. 1999). While increases in extracellular HSP72 are not necessarily representative of intracellular concentrations of HSP72, this finding demonstrates support for the efficacy of passive heating for increasing concentrations of HSPs, which are well‐demonstrated cellular chaperones with the potential to confer physiological benefits.

#### 5.1.2 HSP90

HSP90 is an essential cofactor in the production of nitric oxide via endothelial nitric oxide synthase, exerting direct effects on the endothelium and, by extension, the vasculature (García‐Cardeña et al. 1998; Pritchard et al. 2001; Brouet et al. 2001). HSP90 facilitates the dissociation of endothelial nitric oxide synthase from caveolin, initiating a cascade resulting in nitric oxide production and release (Pritchard et al. 2001; Gratton et al. 2000). HSP90 also has a critical role in the regulation of other nitric oxide synthase isoforms, such as neuronal nitric oxide synthase, that are expressed in nonendothelial cells (Song et al. 2001). The important interdependence of HSP90 and nitric oxide has been demonstrated across multiple vascular beds, including the cerebral (Khurana et al. 2000), mesenteric (Shah et al. 1999), and pulmonary (Su and Block 2000) circulations.

HSP90 is a molecular chaperone that is essential for the proper folding of immature endothelial nitric oxide synthase proteins (Billecke et al. 2002). Furthermore, the binding of endothelial nitric oxide synthase to HSP90 prevents the degradation of both proteins (Averna et al. 2008). Indeed, conformational changes to HSP90 reduce the production of nitric oxide and thereby result in greater superoxide production (Pritchard et al. 2001).

Brunt and colleagues reported that although HSP90 protein abundance was increased in peripheral blood mononuclear cells following a single exposure to heat stress, this increase in HSP90 was not maintained following 8 weeks of hot water immersion (Brunt, Weidenfeld‐Needham, et al. 2019).

#### 5.1.3 HSPs and Mitochondrial Adaptations

Mitochondrial dysfunction is characterized by reduced mitochondrial deoxyribonucleic acid (DNA), decreased respiratory activity, and impaired fatty acid oxidation. Mitochondrial dysfunction also contributes to the pathophysiology of type 2 diabetes (Morino et al. 2006; Patti et al. 2003; Petersen et al. 2004). In 1967, Dr. John Holloszy discovered that one of the primary beneficial impacts of exercise on metabolism is the modification of mitochondrial activity (Holloszy 1967). In the same manner that exercise can improve muscle mitochondrial function, direct limb heating via diathermy has also been shown to increase mitochondrial respiration (Hafen et al. 2019; see detailed discussion in skeletal muscle section). A greater understanding of the effects of HSP72 on mitochondrial function has come from preclinical rodent models and cell systems. In C2C12 myocytes, heat exposure results in an increase in HSP72 protein expression and mitochondrial biogenesis after 1 h of 40°C heat (Liu and Brooks 2012). Importantly, whole‐body maximum oxygen consumption improves when HSP72 is overexpressed in rodent skeletal muscle (Henstridge et al. 2014). This overexpression is a result of corresponding increases in muscle mitochondrial content and respiration (Henstridge et al. 2014). Conversely, mice lacking HSP72 have a disrupted mitochondrial morphology, fatty acid oxidation, and insulin sensitivity (Drew et al. 2014). Pharmacologically increasing HSP72 expression with matrine, a drug typically used to treat human liver tumors, results in improved hepatic palmitate oxidation, resting oxygen consumption, and lipid utilization (Zeng et al. 2015). In the absence of HSP72 in primary murine hepatocytes, mitochondrial structure is altered along with a reduction in mitochondrial fatty acid oxidation (Archer et al. 2018), suggesting that reductions in HSP72 may contribute to hepatic mitochondrial dysfunction.

One possible mechanism of HSP72 regulation of mitochondrial function may be enhancing mitochondrial quality control via mitophagy, or the targeted degradation of mitochondria through autophagy. For example, in mice lacking skeletal muscle HSP72, there is a decrease in mitochondrial degradation via mitophagy. This is a result of the enlarged and dysmorphic mitochondria with reduced respiratory capacity in the HSP72 mice (Rahman et al. 2007).

Heat shock cognate 70 (Hsc70) is involved in chaperone‐mediated autophagy (CMA), a form of autophagy that selectively degrades proteins containing motifs that chemically resemble the pentapeptide sequence, KFERQ (Kaushik and Cuervo 2018). Hsc70 recognizes proteins containing specific motifs, including mitochondrial proteins and enzymes involved in triglyceride synthesis and lipid transport, and targets them to the lysosome for degradation (Kirchner et al. 2019; Schneider et al. 2014). Schneider et al. (2014) determined that CMA is critical for normal hepatic mitochondrial function by showing that mice lacking CMA activity have reduced maximal hepatic mitochondrial respiration and increased lipid accumulation. In response to a lipid challenge, hepatic mitochondria from these mice do not increase mitochondrial respiration as normally expected, and they also display impairments in fatty acid oxidation when compared to control hepatocytes. Overall, these data implicate CMA as a critical regulator of mitochondrial and lipid metabolism, particularly in the liver.

Von Schulze et al. (2021) showed that CMA is activated with heat treatment and may directly benefit hepatic mitochondrial function. Acute heat exposure (42°C for 20 min) increased Hsc70 content in hepatic mitochondrial fractions and the binding of both Hsc70 and ubiquitin to complexes II and IV of the electron transport chain. These data suggest that heat exposure drives the degradation of specific mitochondrial proteins through CMA activation. In this study, heat therapy (9 sessions at 42°C every 72 h for 4 weeks) improved mitochondrial respiration. Together, these data suggest that heat treatment (i.e., heat therapy) may selectively induce the degradation of poorly functioning mitochondrial proteins through mitophagy/autophagy to improve mitochondrial function as a compensatory adaptation to recurrent heat stress.

Additional studies are needed to refine the effects of heat therapy on HSPs' expression. These investigations need to consider different modalities of heat, diverse participant populations, and cellular and tissue origins. While the complex and integrative nature of the HSPs' response makes cell‐ and tissue‐specific detection challenging, accumulating data in the literature points to the multifaceted actions of HSPs in multiple tissues.

### 5.2 Reductions in Oxidative Stress

There is compelling evidence of a strong interplay between inducible HSPs and increased concentration of manganese superoxide dismutase, offering a link between heat therapy and increased resilience to superoxide and hydrogen peroxide. Suzuki et al. (2002) report that the cellular chaperone HSP72 mediates an increase in superoxide dismutase activity, which serves to scavenge free radicals and reactive oxygen species following ischemia–reperfusion injury. Additionally, in an animal model, both acute and chronic heat exposure upregulate HSP70 and superoxide dismutase concentration, while both HSP27 and HSP70 have been shown to attenuate heat exposure‐induced increases in reactive oxygen species (Sreedhar et al. 2002; Belhadj Slimen et al. 2014).

The evidence for the beneficial impact of both acute and chronic heat exposure is well‐documented in cell and animal models. Unfortunately, this does not extend to investigations conducted in humans. There is still a dearth of evidence on heat therapy's beneficial impact in reducing reactive oxygen species. Brunt and colleagues provide insight into the mechanisms that mediate these improvements in their isolated cell work (Brunt et al. 2018). Both the isolated heat treatment of cells (warming to 39°C) as well as exposure of cells to serum from humans who had completed 8 weeks of passive heat therapy reduced basal oxidative stress relative to thermoneutral control cells (37°C) and culture with serum from individuals who did not participate in heat therapy (Brunt et al. 2018). Furthermore, heat treatment and cell culture with serum from those who had completed heat therapy elicited lower concentrations of superoxide anions in response to a hypoxia–reoxygenation insult (Brunt et al. 2018).

Heme oxygenase‐1, otherwise known as HSP32, plays a primary role in the regulation of vascular inflammation, protecting the vasculature from oxidative stress and inflammation (Araujo et al. 2012). Brunt and colleagues reported that cell culture with serum from individuals who have completed 8 weeks of heat therapy prevents the suppression of HSP32 induction following a hypoxia–reoxygenation insult (Brunt et al. 2018). These beneficial acute and chronic responses to heat stress and heat therapy demonstrate strong benefits for improved vascular function and cellular resilience to stress with passive heating.

### 5.3 Proteostatic Mechanism of HSPs

Proteostasis is a concept that encompasses the maintenance of protein homeostasis via the regulation of concentration, conformation (i.e., folding), transport, and turnover. HSPs, which are upregulated in response to nearly all forms of acute heat exposure, play an integral role in helping to maintain proteostasis in numerous tissues and cells. Their many proteostatic functions include the folding of new proteins, refolding of damaged proteins, degradation of nonfunctional proteins, and the import/export of proteins into and out of the mitochondria (Willmund et al. 2013; Frydman 2001; Parsell and Lindquist 1993; Drew et al. 2014; Hartl et al. 2011). As a result, changes in HSP expression and localization are linked to numerous disease states. Neuronal cells, for example, may be particularly susceptible to proteotoxic insult as a result of age‐related declines in HSP, leading to an increased risk of AD (Hetz and Saxena 2017; Kaushik & Cuervo, 2015). The link between Type 2 diabetes and AD risk may also be related to the loss of proteostatic processes in the brain (leading to the development and accumulation of plaques and lesions) as well as in peripheral tissues like skeletal muscle.

### 5.4 Protein Synthesis

Early work focused on elucidating a role for heat therapy in skeletal muscle growth and protein synthesis and was pioneered by Goto et al. One of their initial papers showed that incubation of L6 myoblasts at 41°C for 60 min increased total protein content, leading to the hypothesis that passive heat exposure may acutely increase muscle protein synthesis and/or decrease protein breakdown rates (Goto et al. 2003). Subsequent animal and cell culture models have generally supported this hypothesis. For example, studies in both C_2_C_12_ and L6 myoblasts showed that acute heating (41°C for 20–30 min) increased activation of protein synthesis pathways, including the mammalian target of rapamycin (mTOR) and Phosphoinositide 3‐kinase/Akt signaling pathways (Obi et al. 2019; Moon et al. 2003). In rodents, hot water immersion of the hind limb for 30 min also resulted in increased mTOR and AKT pathway activation (Yoshihara et al. 2013). When hot water immersion is used concomitantly to resistance exercise in humans, multiple lines of evidence point to activation of the AKT/mTOR/FOXO signaling axis (Ihsan et al. 2020; Kakigi et al. 2011). Interestingly, Fuchs et al. showed that hot water immersion did not alter post‐resistance training myofibrillar protein synthesis rates despite increased mTOR pathway component activation. This study is a good example of a case where activation of molecular signaling events associated with downstream outcomes may not always generate those outcomes. Though the methodology to measure protein synthesis and breakdown rates is available in the form of stable isotope tracer studies, to our knowledge, no research to date has directly measured the effect of acute muscle heating on protein synthesis rates in the absence of exercise. Thus, the extent to which passive heat therapy can induce an independent protein synthesis response is still unknown.

### 5.5 Markers of Inflammation and Metabolism

Hoekstra and colleagues examined the acute and chronic effects of hot water immersion on markers of inflammation and metabolism in overweight sedentary men (Hoekstra et al. 2018). Their data demonstrated that acute hot water immersion is capable of increasing plasma interleukin (IL)‐6 concentrations (Hoekstra et al. 2018). There was no significant change in plasma IL‐6 after chronic hot water immersion (Hoekstra et al. 2018). Although Hoekstra et al. reported no change in plasma IL‐6 following heat therapy, there is still the potential that the acute change in IL‐6 acts along a similar pathway as that shown to stimulate anti‐inflammatory pathways following exercise (Steensberg et al. 2003). Namely, the increase in concentration of IL‐6 has been shown to increase concentrations of IL‐10 and IL‐1Ra, which both act in an anti‐inflammatory manner (Steensberg et al. 2003).

Improvements in metabolic and inflammatory profiles following heat therapy via hot water immersion have been demonstrated across a diverse participant population. Ely and colleagues examined the ameliorative effects of heat therapy on both metabolic and inflammatory profiles in women with PCOS (Ely, Clayton, et al. 2019). They report that serum IL‐6 and tumor necrosis factor (TNF) are reduced following heat therapy (Ely, Clayton, et al. 2019). Furthermore, IL‐1 β and IL‐8, assessed in stromal vascular fraction measured via adipose biopsy, were reduced following heat therapy (Ely, Clayton, et al. 2019).

Type 2 diabetes is one condition that has been associated with chronic low‐grade inflammation, characterized by increased circulating levels of proinflammatory proteins (Bastard et al. 2006), and has been the target of investigations on the impact of heat therapy. For example, elevation of C‐Jun‐N‐Terminal Kinase (JNK) is known to impair insulin sensitivity and inhibit insulin signaling (Hotamisligil 2006). Proinflammatory cytokines like JNK and nuclear factor kappa B (NF‐kB) can prevent tyrosine phosphorylation of insulin receptor substrate‐1 (IRS‐1), leading to impaired insulin signaling and decreased glucose uptake (Hotamisligil 2006). The role of the cytokine IL‐6 in inflammation and Type 2 diabetes is less clear. Some studies report a positive association between IL‐6 concentration, insulin resistance, atherosclerosis, Type 2 diabetes, and cardiovascular disease (Dorsey et al. 1996; Hooper et al. 2014; Pradhan et al. 2001; Vozarova et al. 2001). However, transient increases in plasma IL‐6 can enhance insulin action and glucose uptake (Carey et al. 2006), while chronic increases may inhibit insulin signaling (Dandona et al. 2004).

Prior studies in humans indicate that hot water immersion results in an increase in circulating IL‐6 (Krause et al. 2015; Welc et al. 2012). A dose‐dependent effect of heat on IL‐6 may exist, as a 2‐h hot water immersion produced a greater increase in IL‐6 than a 1‐h hot water immersion. Interestingly, several studies have measured changes in TNF‐α in response to acute heat exposure in humans, but significant differences in circulating levels were not observed (Hashizaki et al. 2018; Leicht et al. 2015). Heat therapy of 4 weeks of sauna bathing did not alter IL‐6 or IL‐10 levels in healthy men (Zychowska et al. 2017). However, Behzadi et al. (2022) observed an acute inflammatory response following acute heat exposure with traditional sauna. They reported that 30 min of sauna (in 10‐min increments at 80°C) resulted in an increase in IL‐6 at 60 min following the heat exposure.

HSPs also have a role in mediating inflammatory responses. HSP72 expression in the liver corresponds with increasing disease progression of metabolic dysfunction‐associated steatotic liver disease (MAFLD) in human Kupffer cells (Di Naso et al. 2015). As HSP72 is induced with heat exposure in liver‐specific macrophage Kupffer cells, TNF‐α is suppressed (Liang et al. 2009; Yonezawa et al. 2001). The ability of extracellular HSP72 to inhibit inflammatory cytokines in metabolic tissue could be one way in which these chaperones decrease local inflammation and insulin resistance.

C‐reactive protein is released from the liver in response to inflammatory cytokines. C‐reactive protein is a global marker of inflammation and has been identified as a reliable biomarker for stratifying risk for cardiovascular events, with particular utility in the initial stages of disease manifestation and progression (Pearson et al. 2003; Wilson et al. 2008). Ely and colleagues report that heat therapy significantly reduced C‐reactive protein, indicating reduced global inflammation in women with PCOS (Ely, Clayton, McCurdy, et al. 2019).

Reductions in inflammation may mediate some of the reported benefits of lifelong sauna use on cardiovascular and all‐cause mortality, as described in a series of papers from the Kuopio Ischemic Heart Disease Risk Factor Study (Kunutsor et al. 2018; Laukkanen and Laukkanen 2018). Among the men included in the prospective cohort study, there was a significant inverse relationship between frequency of sauna bathing and measured C‐reactive protein. This relationship persisted after a multivariate analysis accounting for age, body mass index, systolic blood pressure, smoking status, Type 2 diabetes, history of myocardial infarction, and serum low‐density lipoprotein (Laukkanen and Laukkanen 2018).

## 6 Adaptations in Organs and Systems Following Heat Therapy

This section describes the effects of heat therapy on acute and long‐term adaptations at the organ (e.g., skeletal muscle, brain) and system level (e.g., cardiovascular, autonomic) in young and older humans. We have outlined the applicable chronic heat therapy studies in the text and Tables 1, 2, 3 separated by study and modality.

### 6.1 Skeletal Muscle Health, Function, and Metabolism

**FIGURE 5:**
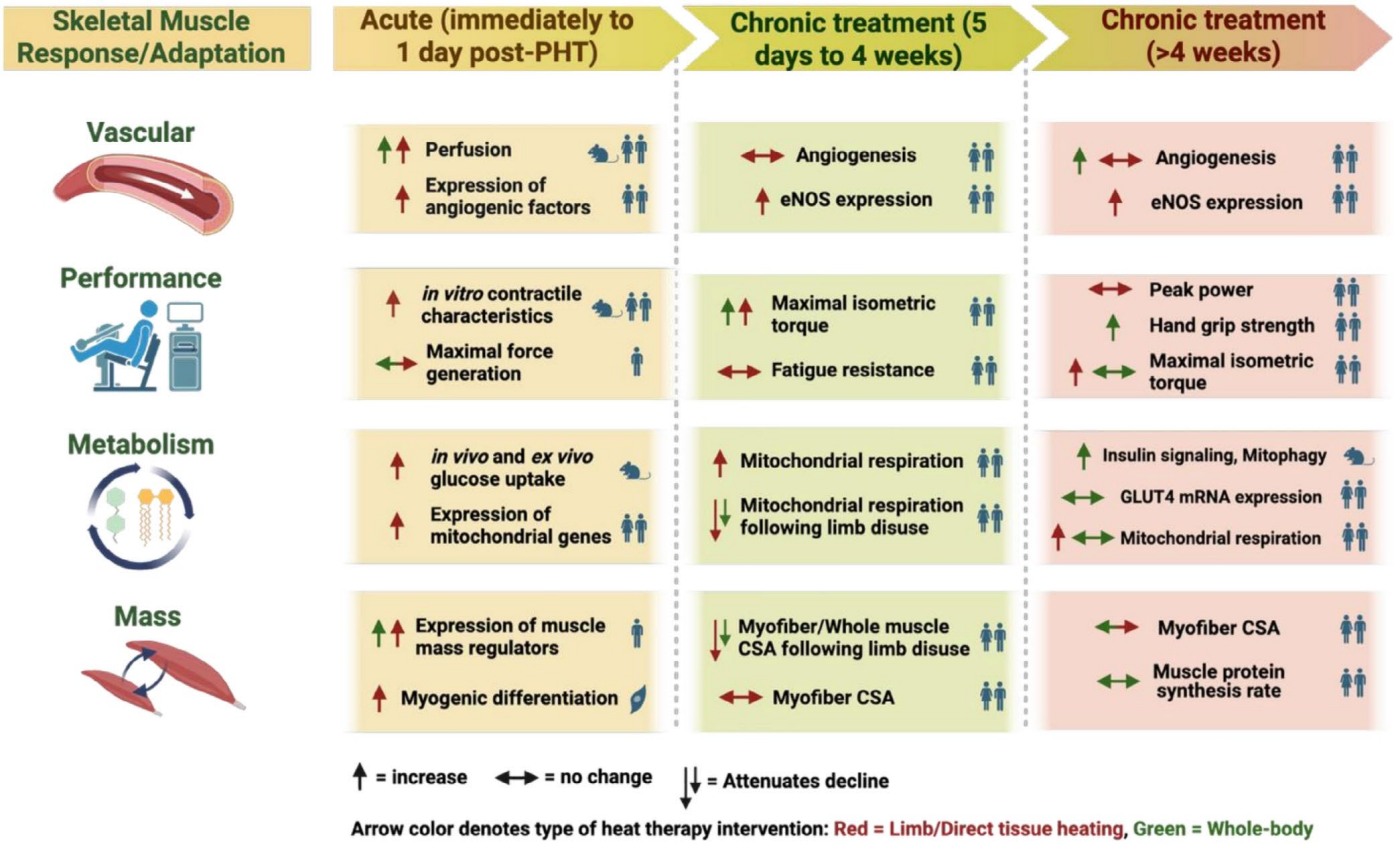
Overview of acute and chronic skeletal muscle responses to heat therapy. This schematic summarizes findings from various heat therapy protocols across four commonly studied outcome domains in skeletal muscle: Vascular function, performance, metabolism, and mass. Arrows indicate both the direction of reported effects and the type of heat intervention. Upward arrows: Consistent increases across studies. Bidirectional arrows: No overall change. Double downward arrows of different sizes: Attenuation of a decline. Arrow color reflects the heating method: Red for limb or direct tissue heating, and green for whole‐body heating. Where multiple and differing colored arrows are present, the variable has been tested under both direct and whole‐body interventions. The experimental model in which the response has been observed‐ human (men/women), animal (mouse), or cell culture (cell)—is also noted. *Created with Biorender.*

The skeletal muscle is of vital importance for glucose metabolism, insulin‐mediated glucose uptake, and blood pressure regulation (Merz and Thurmond 2020; Richter and Hargreaves 2013; Joyner and Casey 2015), in addition to the obvious role in movement. Others have written exceptional reviews detailing the role of skeletal muscle in these physiological processes; therefore, we will not go into detail regarding them. Our purpose is to summarize the acute effects of heat exposure on skeletal muscle health and function, and focus our discussion primarily on the impact of chronic heat exposure on this organ system and its functions (Merz and Thurmond 2020; Richter and Hargreaves 2013; Joyner and Casey 2015). We have provided a summary figure (Figure 5) that provides an overview of the responses discussed in this section.

#### 6.1.1 Effect of Acute Heat Exposure on Skeletal Muscle Health and Function

To understand heat therapy's potential benefits on skeletal muscle, it is important to first understand the acute responses to heat exposure. Resting muscle temperature is typically measured to be ~34.5°C–36.5°C, with depth of both the muscle and in‐dwelling thermocouple significantly influencing the measurement. To date, muscle temperature data are generally limited to the muscles of the knee extensors (Saltin et al. 1968), plantar flexors (Selkow et al. 2012), elbow flexors, and elbow extensors (Draper et al. 1998), with the most extensive characterization being focused on the *vastus lateralis* muscle. Short‐wave diathermy has been repeatedly shown to increase intramuscular temperature by 3.5°C–4.0°C (Hafen et al. 2018; Mangum et al. 2022; Richey, Ruiz, et al. 2024). Smaller changes in *vastus lateralis* intramuscular temperature are achieved using lower body hot water immersion (40°C–42°C), with studies reporting increases of 2.4°C (Rodrigues et al. 2023, 2021) and 2.3°C (Fuchs et al. 2020). However, in a uniquely extreme study, (Morton et al. 2007) managed to increase intramuscular temperature 3.6°C by immersing a single limb in 45°C water for 1 h. Water‐perfused garments have also increased muscle temperature, with the perfusion of 46°C water for 30 min increasing gastrocnemius intramuscular temperature by 3.7°C (Keller et al. 2010; Kuhlenhoelter et al. 2016).

Lastly, exposure to traditional sauna appears to have a more modest effect on intramuscular temperature, with 40 min of acute traditional sauna exposure (2 × 20‐min sauna at 80°C separated by 10‐min recovery at 23°C) increasing skeletal muscle temperature by only 2°C (Leach et al. 2024). It is widely claimed that far‐infrared sauna may increase intramuscular temperature to a greater degree than dry sauna due to the deeper penetration of infrared waves. Reed and colleagues reported that the increase in muscle temperature with a far‐infrared sauna is muscle depth dependent. While superficial muscle temperature increased by 3.0°C, the middle depth increased by 1.9°C, and the deep muscle increased by 1.1°C (Reed et al. 2025). Notably, changes in muscle temperature appear negligible beyond 3.8 cm below the skin surface. More practically, the thermic effect had lessened by 63% at a depth of 2.4 cm, which can be considered effective thermal penetration. It is currently unknown whether acute dry or infrared sauna exposure elicits a skeletal muscle heat shock response.

#### 6.1.2 Blood Flow

Few studies have comprehensively assessed how acute heat exposure affects the extent and distribution of blood flow in skeletal muscle. Some have reported increases in skeletal muscle blood flow ranging from 1.5–4.0 fold following a wide range of both local and whole‐body heating modalities (Richey, Ruiz, et al. 2024; Keller et al. 2010; Sekins et al. 1984). Changes in skeletal muscle blood flow have been shown to represent a significant portion of the increased systemic perfusion that occurs with passive heat stress (Pearson et al. 2011). Nevertheless, there is some discrepancy in the literature, with some studies showing no effect of passive heat stress on muscle perfusion (Detry et al. 1972; Sekins et al. 1980). These discrepancies are most likely due to different heating techniques and the degree to which the modality achieves increases in muscle tissue temperature. The mechanisms underlying the skeletal muscle hyperemic response are still poorly understood (Hyldahl et al. 2021; Richey, Ruiz, et al. 2024; Pearson et al. 2011). Heating muscle appears to upregulate the angiogenic factors VEGF and ANG2 (Kuhlenhoelter et al. 2016). While this wouldn’t contribute to acute hyperemia, it could potentiate an increase in skeletal muscle vascularity and muscle hyperemia with chronic heat therapy. In fact, as discussed in the following section on chronic heat exposure, there is some evidence for repeated passive heating to induce vascular remodeling in both human and in vitro models.

#### 6.1.3 Metabolism

Acute heat stress has also been reported to improve insulin sensitivity, skeletal muscle glucose transport, and markers of mitochondrial function in several experimental preclinical models. While glucose kinetic studies have not been performed on isolated muscle in humans, there is evidence that acute bouts of heat may impact whole‐body glucose kinetics (Leicht et al. 2019). Human trials have also shown that whole‐body and localized heating modalities acutely induce the expression or activation of proteins associated with energy sensing and mitochondrial biogenesis in the muscle, including AMPK, ERK1/2, NRF1, NRF2, and COX2 (Hafen et al. 2018; Ihsan et al. 2020). On the other hand, Kwon et al. (2022) showed no evidence of increased mRNA expression of genes associated with mitochondrial biogenesis following a 4‐h treatment with a heated thermal wrap. As this study did not report intramuscular temperature, the extent to which the thermal wrap altered this variable is unclear. The impact of heat therapy on skeletal muscle glucose uptake, insulin sensitivity, and metabolism remains a relatively untapped area of research that will be important as we consider the rising epidemic of Type 2 diabetes and other metabolic diseases.

#### 6.1.4 Effect of Chronic Heat Exposure on Skeletal Muscle Health and Function

Given the widespread effects of acute heat exposure on skeletal muscle, it is unsurprising that a chronic application of passive heat exposure results in broader changes to muscle function and phenotype, much like how physiological alterations to acute exercise manifest in the chronic adaptations of exercise training. Much of what we know about the effects of heat therapy has been derived from exploring the concept that heat exposure may replicate or enhance the effects of exercise training.

The success of early acclimation studies in promoting cardiovascular and muscular adaptations during exercise likely inspired more recent theories regarding the use of heat therapy. This section will review the evidence for heat therapy to induce skeletal muscle adaptation. As exercise training promotes favorable health‐promoting adaptations, much of the work with heat therapy has assessed similar metabolic, vascular, and hypertrophic outcomes that would be expected with exercise training.

#### 6.1.5 Metabolism

Skeletal muscle accounts for upwards of 80% of glucose uptake in the presence of insulin, and previous data (James et al. 2023; Hooper 1999; Kokura et al. 2007) would suggest that heat therapy likely improves glucose transport across skeletal muscle. Hesketh et al. (2019) attempted to provide insight into this question by measuring GLUT4 mRNA content in human *vastus lateralis* muscle after 6 weeks of passive heat therapy in a heat chamber (40–50 min at 40°C on 3 days per week). Under this protocol, they found no change in skeletal muscle GLUT4 mRNA content (Koshinaka et al. 2013). However, GLUT4 mRNA content does not necessarily reflect the protein content, nor the propensity for insulin‐stimulated GLUT4 translocation. Moreover, their heating protocol did not raise core temperature and was not likely to alter intramuscular temperature significantly.

A growing body of literature suggests that heat therapy improves whole‐body insulin sensitivity in Type 2 diabetics (Hooper, 1999; James et al. 2023; Gupte et al. 2009). However, much more research is necessary to investigate the possible muscle‐centric mechanisms that may underlie this effect.

Few studies have directly measured the impact of heat therapy on insulin‐stimulated glucose uptake in the muscle; a substantial number of studies have reported its impact on muscle mitochondrial biogenesis and function. Chronic heat exposure (60 min at 40°C daily for 5 days) was first demonstrated by Liu and Brooks (Selkow et al. 2012) to increase markers of mitochondrial biogenesis in C_2_C_12_ myotubes. In 2018, Hafen et al. (2018) extended this finding to human skeletal muscle using short‐wave diathermy. In that study, six consecutive days of passive diathermy heating (2 h per day) improved maximal coupled and uncoupled respiratory capacity and increased expression of electron transport protein complexes in untrained men and women. Six weeks of passive *vastus lateralis* diathermy treatments (2 h per day, 3 days per week) were also reported to increase skeletal muscle mitochondrial function to a similar extent as 6 weeks of single‐leg knee extension exercise training (Marchant et al. 2022). Despite this growing consensus, data on human skeletal muscle mitochondrial adaptation are not consistent across all studies. For example, Hesketh and colleagues (Hesketh et al. 2019) reported that 40–50 min of passive heating in a heat chamber (40°C, 3 days per week for 6 weeks) did not significantly alter cytochrome c oxidase expression in human *vastus lateralis* muscle. Likewise, Kim et al. (2020) recently reported that neither 4 nor 8 consecutive weeks of heat therapy using a garment perfused with hot water (52°C) altered citrate synthase activity or the expression of protein complex subunits of the electron transport chain. Nevertheless, comparison of these human studies may be nuanced due to the differing heat modalities (i.e., water‐perfused garment vs. diathermy vs. whole‐body heating), duration of intervention, and varying methodologies for measuring mitochondrial adaptation. Whereas Kim et al. and Hesketh et al. measured surrogates (i.e., citrate synthase activity or cytochrome c oxidase expression) for mitochondrial function/content, Hafen et al. and Marchant et al. assessed mitochondrial respiratory capacity more directly via high‐resolution respirometry. Furthermore, while unlikely, it is possible that the application of electromagnetic radiation in the form of short‐wave diathermy may drive adaptation independently of the generation of heat.

#### 6.1.6 Vascular Remodeling

Given the broad impact of heat therapy on skeletal muscle blood flow, there has also been significant interest in the impact of heat therapy on skeletal muscle vascular remodeling. Studies in cultured endothelial cells have suggested that periods of hyperthermia are capable of stimulating the release of vasoactive and angiogenic factors (Harris et al. 2003). Kuhlenhoelter et al. (2016) showed that an acute 90‐min exposure to heat therapy upregulated mRNA expression of several proangiogenic factors in *vastus lateralis* muscle, suggesting that heat therapy may promote angiogenic adaptation. However, studies using heat therapy in humans have been somewhat conflicting. For example, Kim, Reid, et al. (2020) showed that 8 weeks of thigh heating using a water‐perfused garment attenuated a temporal decline in capillary density around type 2 muscle fibers. Additionally, Hesketh et al. (2019) observed enhanced skeletal muscle capillarization after 6 weeks of whole‐body heating, which produced similar results to those seen with the same duration of moderate intensity exercise training. Fuchs et al. (2025) also reported an increase in Type I and Type II fiber‐associated capillary density, as well as improved muscle microvascular perfusion kinetics in older participants following 8 weeks of 3 days per week infrared sauna therapy.

Others have reported that 6 weeks of knee extensor diathermy treatments (2 h, 3 days per week) did not alter *vastus lateralis* capillary density in young healthy participants (Kaluhiokalani et al. 2025). The discrepancy in these human studies is likely due to varying heating durations and modalities. While local heating modalities produced minor or no change in muscle capillarity, whole‐body interventions appear to generate a more consistent and robust effect. Therefore, it may be reasonable to suggest that whole‐body heat therapy interventions might offer a stronger stimulus for angiogenic remodeling in muscle.

#### 6.1.7 Muscle Performance Following Heat Therapy

Based on the premise that heat therapy improves muscle mitochondrial function (Liu and Brooks 2012), vascularization (Kuhlenhoelter et al. 2016), and proteostasis, it seems likely also to promote improvements in skeletal muscle functional characteristics. As discussed in the prior section, passive heating of the thigh for 8 h per day for 10 weeks was shown by Goto and colleagues to improve knee extensor maximal isometric torque by 5.8% in young participants (Goto et al. 2011). Using a more practical passive heating protocol of 90 min per day (Kim, Monroe, et al. 2020; Kim, Reid, et al. 2020) likewise found improvements in knee extensor isometric force production after 4 and 8 weeks of treatment. Interestingly, the improvement in force‐generating capacity was not accompanied by a change in myofiber cross‐sectional area. Eleven days of whole‐body heat therapy (1 h per day, 48°C–50°C heat chamber) improved maximal voluntary torque production and evoked peak twitch amplitude of the plantar flexor muscles (Racinais et al. 2017). However, it must be noted that this study did not include a control group.

Most recently, Kaluhiokalani et al. (2025) and Marchant et al. (2022) compared performance characteristics following either 6 weeks of knee extensor passive heating (diathermy, 3 days per week, 2 h per session) or 6 weeks of single‐leg knee extension exercise training. Despite observing similar improvements in muscle mitochondrial function and vascular hemodynamics in both groups, unlike exercise training, heat therapy did not improve peak power, critical power, or work prime during a knee extension graded exercise test. This mismatch may stem from persistent constraints on oxygen transport at VO_2_max, implying that passive heating does not substantially alter the central or peripheral determinants that cap maximal aerobic capacity. In support of this idea, 6 weeks of single‐leg knee extension training increased peak blood flow during exercise, whereas passive heating produced no such change during the identical task (Kaluhiokalani et al. 2025). Given that exercise performance depends on a complex interaction of mechanical, neural, metabolic, and cardiovascular demands, the lack of change in peak function suggests that passive heating alone is insufficient to stimulate the full range of adaptations required to raise maximal exercise capacity. In terms of strength performance, Fuchs et al. recently reported that 8 weeks of infrared sauna did not improve 1‐repetition maximum (1RM) leg press or 1RM leg extension performance in older adults, but participants had a modest improvement in handgrip strength (Fuchs et al. 2025). Though data are still limited, heat therapy alone does not seem to alter exercise capacity but may positively affect maximal torque generation of lower extremity muscles.

#### 6.1.8 Muscle Mass

Lastly, there is limited evidence that heat therapy may influence muscle mass. As discussed previously, studies in humans generally agree that acute bouts of heat exposure activate pathways associated with skeletal muscle protein synthesis and hypertrophy. Extensive in vitro, animal, and human research by Goto and colleagues was the first to clarify the impact of heat therapy on muscle fiber size and strength. For instance, when they cultured C_2_C_12_ cells at 39°C for 5 days, they found greater upregulation of myofibrillar genes and myogenic differentiation compared to cells cultured at 37°C (Guo et al. 2017). Using a water‐perfused garment, Kim, Reid, et al. (2020) found that lower limb passive heating (three sessions per week for 4 and 8 weeks) did not significantly change *vastus lateralis* myofiber CSA but did slightly improve peak isokinetic knee extensor torque. Likewise, Fuchs et al. (2025) found that 8 weeks of infrared sauna therapy had no effect on *vastus lateralis* myofiber CSA or muscle protein fractional synthetic rates in older adults (65–85 years). Interestingly, they did report a modest but detectable increase in handgrip strength (Fuchs et al. 2025). Although the data remain limited, hypertrophic and functional adaptations due to passive heating seem minimal at best.

However, while chronic passive heating might not effectively stimulate muscle growth, an increasing number of studies in animals and humans suggest it could help prevent muscle atrophy from limb disuse or denervation.

#### 6.1.9 Mitigation of the Adverse Effects of Muscle Disuse

**FIGURE 6:**
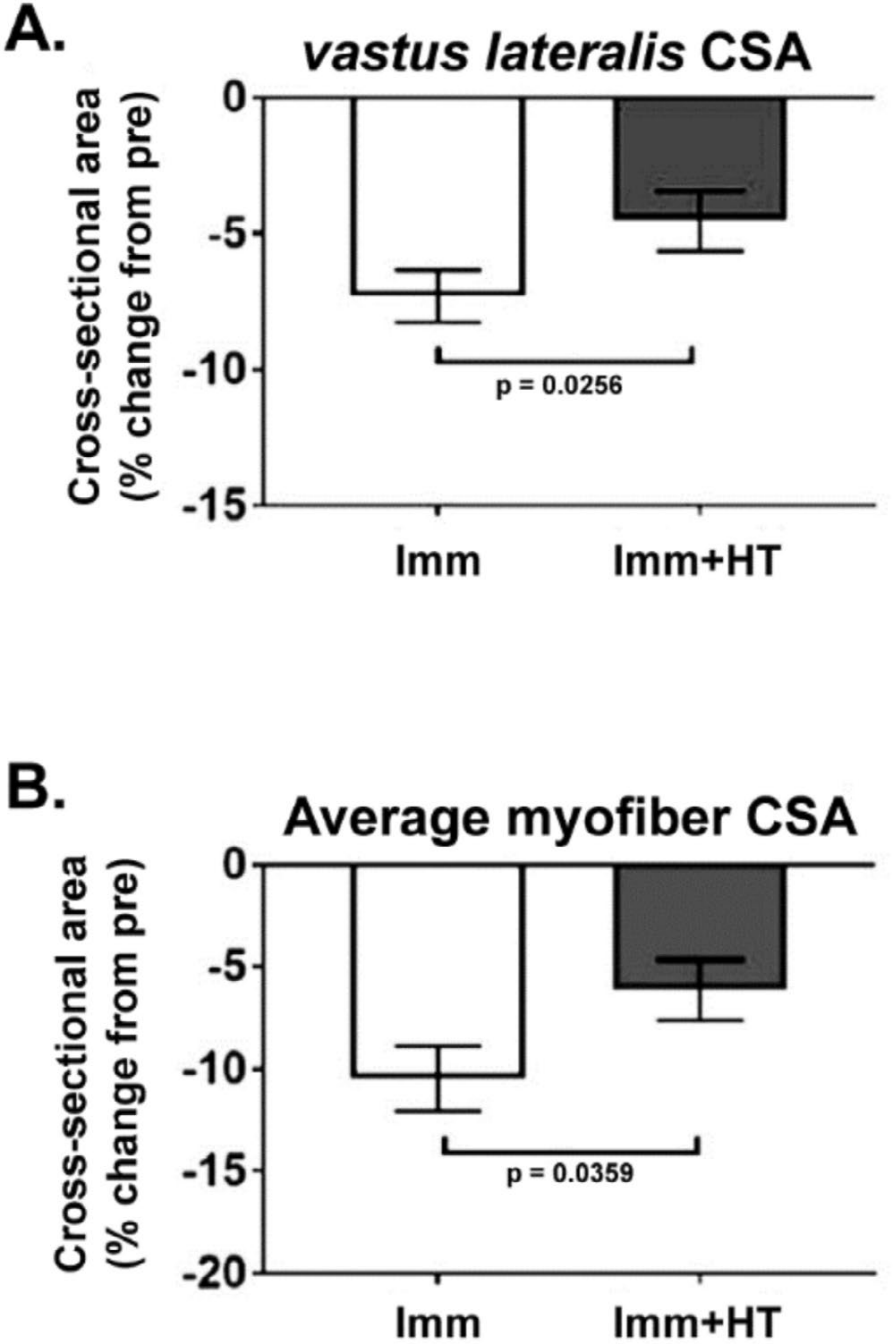
Hafen et al. report the effect of 10 days of lower limb immobilization (Imm) with (Imm + HT) and without (Imm) a daily 2 h diathermy treatment (HT) on *vastus lateralis* whole muscle (A) and myofiber (B) cross‐sectional area (CSA). Data are presented as means ± SE. Imm *n* = 11, Imm + HT *n = 12*. Figure used with permission. Previously published in the *Journal of Applied Physiology* (Figure 5; Hafen et al. 2019).

While there is limited evidence that heat therapy facilitates skeletal muscle hypertrophy, there is compelling data that it may mitigate atrophy during periods of disuse. Hafen et al. (2019) first showed that daily 2‐h heat therapy via short‐wave diathermy during 10 days of lower limb immobilization was sufficient to reduce *vastus lateralis* atrophy at the whole muscle and myofiber level (Figure 6). Additionally, limb immobilization reduced maximal myofiber respiratory capacity by 27% of baseline, yet daily heat exposure almost completely attenuated this reduction. Labidi et al. (2024) also recently showed that 60 min of daily whole‐body heat therapy (40°C–50°C heat chamber) was shown to mitigate plantar flexor atrophy following 2 weeks of ankle immobilization. While the mechanisms underlying this effect are not completely understood, Hyldahl et al. reported that heat therapy during 10 days of leg immobilization attenuated the loss in vascular function, suggesting that improved hemodynamics may underlie some of the muscle mass preservation effects. Additionally, activation of HSPs (Fennel et al. 2022; Acquarone et al. 2024), AMPK (Thomson 2018), and improvement in mitochondrial function (Hood et al. 2019) also likely contribute, as each of these factors is independently associated with the regulation of muscle mass during muscle atrophy. The data suggest that heat therapy might be an effective strategy to mitigate muscle atrophy and deconditioning during disuse. However, more studies are needed to answer fundamental questions on this topic. Consequently, passive heating could serve as a countermeasure for muscle degradation, even if it is insufficient to induce hypertrophy on its own.

#### 6.1.10 Passive Heat Therapy as a Therapeutic Modality to Treat and Prevent Muscle Injury

Muscle injuries represent a significant proportion of all sport‐related musculoskeletal injuries, with most of those occurring in one of four major muscle groups: hamstrings, hip adductors, quadriceps, and gastrocnemius/soleus (Ekstrand et al. 2011; Järvinen et al. 2013). Muscle injuries are classified as strains, contusions, or in situ necrosis events (rhabdomyolysis). In contusion injuries, heavy extrinsic compressive forces disrupt muscle fibers and their connective tissues. Strain injuries are a result of excessive intrinsic tensile force. Both strains and contusions are characterized by myofiber necrosis and disruption of basal lamina and connective tissue components. While skeletal muscle is highly vascular and generally possesses a robust regenerative response, severe strains and contusions can often result in fibrotic tissue accumulation and permanent loss of function.

Skeletal muscle can also display injury‐related response patterns collectively called exercise‐induced muscle damage (EIMD). EIMD generally manifests in response to unaccustomed eccentric muscle actions and results in delayed and prolonged muscle soreness (DOMS) and reductions in muscle force‐producing capacity. Unlike strains and contusions, EIMD is not pathologic, does not generally result in myofiber necrosis, and is adaptive in nature. Because EIMD is nonpathologic yet replicates some of the features of muscle strains and contusions (e.g., a mild inflammatory response and functional decrements), it is often used as an experimental model in humans to test the efficacy of therapeutic interventions. While many studies investigate the effect of heat exposure interventions on EIMD, evidence of its therapeutic value in the treatment of more severe muscle injuries (e.g., strain and contusion) is limited and mostly confined to animal models. Here, we will focus on the evidence for heat therapy as a modality to both rehabilitate and prevent severe muscle injury, with only minimal discussion of its effect on EIMD. For a more comprehensive review on the impact of passive heating on EIMD, the reader is referred to a recent review by Kim, Monroe, et al. (2020).

#### 6.1.11 Injury Rehabilitation

The collective physiological effects of passive heating would strongly suggest that it has therapeutic value in the recovery of skeletal muscle from injury. Indeed, heat exposure has been used historically as a therapeutic modality to mitigate pain and promote tissue regeneration (Lubrano et al. 2023). The primary outcomes used to assess the efficacy of passive heat therapy as a postinjury therapeutic modality have typically included pain, tissue extensibility (i.e., joint range of motion), strength, and blood biomarkers of muscle damage (e.g., creatine kinase, myoglobin). Overall, there is a general consensus that heat exposure, when applied post‐EIMD, reduces DOMS and, to a lesser extent, the force loss that results from EIMD (Kim, Monroe, et al. 2020; Petrofsky et al. 2017; Yoshida et al. 2022; Wang et al. 2022). However, the impact of heat exposure on blood biomarkers of muscle damage following EIMD is less certain, as there appears to be more disagreement between studies (Vaile et al. 2008; Jackman et al. 2023).

Studies on the effect of heat exposure on muscle regeneration and long‐term functional outcomes following more severe muscle injuries are limited and largely confined to rodent studies, although there are a few human studies. The few human clinical trials that have evaluated the efficacy of heat therapy also generally concur that there is a positive benefit. For example, nine treatments of short‐wave diathermy (30 min, 3 days per week for 3 weeks), initiated 72 h following muscle injury in athletes, were found to accelerate muscle hematoma resolution after 2 weeks of treatment compared to an ultrasound treatment group (Giombini et al. 2001). More broadly, a recent meta‐analysis of the impact of local heat application in individuals with musculoskeletal disorders concluded that the application of local heat therapy is more beneficial than no treatment to reduce pain and improve physical function in conditions including nonspecific back pain, fibromyalgia, DOMS, and knee osteoarthritis (Clijsen et al. 2022). Nevertheless, the benefits appear to extend mostly to acute conditions vs. chronic conditions (Clijsen et al. 2022). Overall, despite heat therapy being a mainstay of traumatic muscle injury rehabilitation, there is surprisingly little direct evidence in humans of its efficacy to promote better long‐term healing and regenerative outcomes.

### 6.2 Cardiovascular Health and Function

Cardiovascular disease is one of the leading causes of morbidity and mortality across the world (Merz and Cheng 2016; Tsao et al. 2023). Therefore, understanding how the ill effects of cardiovascular disease can be prevented is important to improving health and well‐being across the lifespan. Adverse symptomology of cardiovascular disease often has its beginnings during young adulthood, with an accelerated timeline throughout aging, dependent upon various lifestyle habits (Rippe 2019). Cardiovascular disease and disease risk are due to a combination of factors, such as increased arterial stiffness (Seals 2014; Chirinos et al. 2019; Fu and Ogoh 2019), elevated blood pressure (Taddei et al. 1995; Lakatta and Levy 2003; Boutouyrie et al. 2021; Yanes and Reckelhoff 2011), and endothelial dysfunction (Seals 2014; Hemingway et al. 2022; Somani et al. 2019; Kenney 2017; Black et al. 2009; Yildiz 2007). Most of these effects can be attenuated with consistent exercise training (Seals 2014; Seals et al. 2018, 2006, 2016; Clayton et al. 2022; Desouza et al. 2000), a well‐known gold standard for improving cardiovascular health. Unfortunately, few people engage in a consistent exercise training program; thus, the beneficial nature of this method only extends to the consistent few.

Pharmacological treatments are used to reduce already apparent disease risk and to attenuate further development of risk factors in cardiovascular disease. Statins are used to reduce cholesterol and improve arteriosclerosis, while reducing the risk of coronary artery disease; while antihypertensives reduce blood pressure. Various other medications are also used to improve cardiac function and terminal outcomes of those with progressing cardiac disease. Although helpful, all pharmacological treatments are accompanied by side effects. These can have both positive and negative impacts on health and well‐being.

For these reasons, there has been an enthusiastic scientific interest in whether heat therapy can act as a nonpharmacological treatment to improve cardiovascular disease or disease risk. This excitement was heightened by the work of Laukkanen et al. (2015), their 30‐year prospective analysis of Finnish sauna bathing, which found that habitual/consistent sauna use reduced fatal cardiovascular and all‐cause mortality events in men. Men who completed more sauna sessions per week (4‐7×) for a longer duration per session (> 19 min) had the greatest reduction in sudden cardiac death over the 30‐year follow‐up (Laukkanen et al. 2015).

These results demonstrated to the scientific community the therapeutic potential of chronic heat exposure for improving cardiovascular health. The purpose of this section is to describe each aspect of the cardiovascular system and the adaptive or nonadaptive responses following heat therapy.

#### 6.2.1 Vascular Adaptations

Large artery stiffness is associated with the risk of developing cardiovascular disease and has become a clinical measure to assess disease risk and indicate potential health concerns (Chirinos et al. 2019). Arterial stiffness can be measured using Ankle‐Brachial Pulse Index, Carotid‐Ankle Vascular Index, central pulse wave velocity (carotid‐femoral), and/or peripheral pulse wave velocity (femoral to dorsalis pedis or brachial to ankle). The changes in arterial stiffness due to heat exposure may directly impact changes in blood pressure and vascular tone (Cheng and MacDonald 2019).

Acute heating responses to arterial stiffness are varied due to various population sizes, differentiation of sex differences, and modality of heating and measurement (Cheng et al. 2021; Hu et al. 2012; Thomas et al. 2017; Lee et al. 2018; Ganio et al. 2011; Moyen et al. 2016; Schlader et al. 2019; Caldwell et al. 2017; Pyke et al. 2004). Similarly, heat therapy responses have also been equivocal. Some investigations have reported that young adults have improved arterial stiffness, wall thickness, and compliance following heat therapy (Brunt, Howard, et al. 2016; Hunter, Dhindsa, Cunningham, Hunter, et al. 2013). For example, Bikram yoga improved carotid artery compliance and reduced ß‐stiffness index (Hunter, Dhindsa, Cunningham, Hunter, et al. 2013). Eight weeks of hot water immersion (whole‐body) elicited improvements in aortic pulse wave velocity, β‐stiffness index, carotid wall thickness, and femoral artery compliance (Brunt, Howard, et al. 2016; Hunter, Dhindsa, Cunningham, Hunter, et al. 2013), while an ankle‐foot bath did not induce improvements in arterial stiffness as measured by pulse wave velocity (Cheng et al. 2024).

Age attenuates the health of the vasculature, and age‐related decrements can be further compounded and exacerbated by cardiovascular disease risk factors, such as high cholesterol and high blood pressure (Seals et al. 2006). To characterize the impact of passive heat therapy on vascular function in healthy older adults, Ruiz‐Pick et al. 2025, measured arterial stiffness via pulse wave velocity before and after 8 weeks of heat therapy. Central and peripheral pulse wave velocity, or arterial stiffness, did not improve following heat therapy in these participants (Ruiz‐Pick et al. 2025). Similarly, Bikram yoga improved carotid artery compliance and beta‐stiffness index in young, but not in middle‐aged or older adults (Hunter, Dhindsa, Cunningham, Hunter, et al. 2013). Longitudinal studies have also reported that Bikram yoga interventions can improve brachial‐ankle pulse wave velocity in overweight and obese adults, irrespective of changes in blood pressure (Hunter et al. 2016).

Although the results are equivocal (Brunt, Howard, et al. 2016; Hunter, Dhindsa, Cunningham, Hunter, et al. 2013), the underlying mechanism of improved arterial stiffness is still largely unknown. One potential mechanism is that the increase in shear stress may induce remodeling of the arterial wall. Joannides et al. (2001) demonstrated in vivo that shear stress is directly related to arterial wall stiffness and compliance. By using blood flow occlusion and hand heating, they determined that changes in shear stress (i.e., increases or decreases) were directly associated with the subsequent increases or decreases in radial artery wall stiffness (Joannides et al. 2001).

Shear stress stimulates the release of vasodilators from the endothelium, such as nitric oxide and endothelium‐derived hyperpolarizing factor. Kinlay et al. reported that nitric oxide plays a central role in large artery stiffness (i.e., compliance and pulse wave velocity) (Kinlay et al. 2001). Further, when Bellien and colleagues concurrently inhibited nitric oxide synthase via N(G)‐monomethyl L‐arginine (L‐NMMA) and endothelium‐derived hyperpolarizing factor via tetraethylammonium (TEA), this counteracted the improvements in wall stiffness and vascular smooth muscle tone that are typically observed during local hand heating (Bellien et al. 2010). These data suggest that shear stress could be the initial step in the cascade of arterial stiffness changes following heat therapy.

#### 6.2.2 Cutaneous Vascular Function

Acute heat exposure's effect on cutaneous thermal hyperemia has been well‐documented in the past, with numerous investigations focused on elucidating “THE” vasodilator of the thermal hyperemic response (Fieger 2011; Wong et al. 2006; McCord et al. 2006; Fujii et al. 2014; Fujii, McGinn, et al. 2015; Fujii, Pastore, et al. 2018; Fujii, Paull, et al. 2015; Brunt et al. 2015; Halili et al. 2016; Fujii, Meade, et al. 2016; Fujii, Halili, et al. 2015, 2018; Fujii, McNeely, Nishiyasu, and Kenny 2017; Fujii, Amano, Halili, et al. 2017; Choi et al. 2014; Fujii, McNeely, and Kenny 2017; Fujii, Meade, Akbari, et al. 2017; Louie et al. 2017, 2016; Fujii, Dervis, et al. 2016). Few studies have used a chronic heat exposure model to elucidate the mechanisms driving heat adaptation in the cutaneous microvasculature. Initial investigations into the adaptation of the cutaneous microvasculature to chronic heat exposure were conducted by Carter et al. (2014) and Green et al. (2010).

**FIGURE 7:**
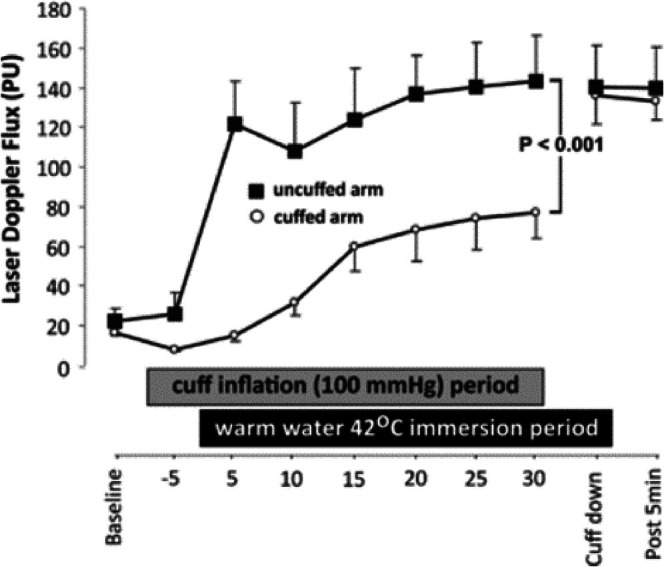
Green et al. investigated the impact of shear stress and repeated skin vasodilation on nitric‐oxide‐dependent dilation. Laser Doppler flowmetry (LDF) as expressed by laser Doppler flux are shown between cuffed (open circles) and uncuffed (closed squares) arms before, during, and after water bath immersion. During the water immersion responses differed between the cuffed and uncuffed arms (*p* < 0.001). Figure used with permission. Previously published in *The Journal of Physiology* (Figure 1; Green et al. 2010).

Green et al. (2010) investigated the impact that shear stress and repeated skin vasodilation would have on nitric oxide‐dependent dilation. To test their hypothesis, they attenuated shear stress in one arm via cuff occlusion while both arms were immersed in hot water (Figure 7). They reported that chronic heat exposure of the arm increased nitric oxide‐dependent dilation and augmented blood flow. Interestingly, these changes were not seen in the arm with cuff occlusion, suggesting that the increase in shear stress was a primary driver of cutaneous microvascular adaptation to heat exposure (Green et al. 2010).

Carter et al. (2014) sought to understand how cutaneous microvascular adaptation occurs with chronic heat exposure. They hypothesized that with chronic heat exposure, or as they state, repeated increases in body core temperature, the cutaneous microvasculature would have an augmented vasodilatory response. In support of their hypothesis, cutaneous vasodilatory responsiveness to thermal hyperemia was increased following 8 weeks of chronic heating (Carter et al. 2014).

Francisco et al. (2017) locally heated one arm for 10 days at 42°C while the other was controlled at 32°C, to investigate whether the cutaneous microvasculature would become heat‐adapted. They also wanted to understand what mechanisms drive that peripheral adaptation. There was no change in cutaneous microvascular function, nor any change in responsiveness to acetylcholine administration (used to interrogate a potential mechanism). These results contrast with those of Brunt, Eymann, et al. (2016), who found increased nitric oxide bioavailability and improved nitric oxide‐mediated dilation following 8 weeks of passive whole‐body heat exposure in physically inactive healthy young adults (Brunt, Eymann, et al. 2016). Taken together, these data illustrate an essential component of heat therapy research, which is considering the method/modality of heat exposure as well as frequency, duration, and time. Brunt, Eymann, et al. (2016) used whole‐body hot tub immersion, whereas Francisco et al. (2017), Green et al. (2010), and Carter et al. (2014) used a localized heating stimulus. It seems likely that differences in heating modalities drive the differences in outcomes.

Work in the future should be directed at understanding what heat stimulus is required for beneficial heat adaptation in the cutaneous microvasculature. Additionally, questions addressed at the mechanistic level could help us to understand not only the drivers of heat adaptation in the skin but also the interactions that various pharmacological substances may have on these mechanisms. This becomes increasingly important as the frequency of heat events increases due to the rising global temperatures.

#### 6.2.3 Endothelial Function

Endothelial function is repeatedly associated with cardiovascular health, and decrements in endothelial function have been correlated to increased cardiovascular disease risk. In particular, the measurement of flow‐mediated dilation, one common assessment of conduit artery endothelial function, has been highly correlated with cardiovascular disease risk (Green et al. 2011).

In young adults, heat therapy has had varied effects on endothelial function. For example, sedentary young adults had improved brachial artery flow‐mediated dilation following 8–10 weeks of hot water immersion (Brunt, Howard, et al. 2016). Another investigation demonstrated that passive leg movement, an alternative method of assessing endothelial function, was improved after 10‐day leg immobilization following heat therapy via diathermy (Hyldahl et al. 2021). They reported that hyperemia was improved for those in the heat therapy group compared to the control group. Furthermore, improvements in microvascular function were not due to an angiogenic response, suggesting that heat therapy improves leg endothelial function despite immobilization (Hyldahl et al. 2021).

Hunter et al. (2017) investigated the impact of Bikram Yoga on cardiovascular health, specifically flow‐mediated dilation in healthy young, middle‐aged, and older adults. Following 8 weeks of Bikram yoga, middle‐aged and older adults demonstrated significantly improved endothelium‐dependent dilation, unlike young adults, who had no improvement (Hunter et al. 2017). However, subsequent investigations, which included a thermoneutral (23°C) control condition involving 90 min of hatha yoga, reported comparable improvements in endothelium‐dependent dilation to the Bikram (40°C) group (Hunter et al. 2018). Therefore, yoga may improve endothelial‐dependent dilation regardless of whether heat is involved.

**FIGURE 8:**
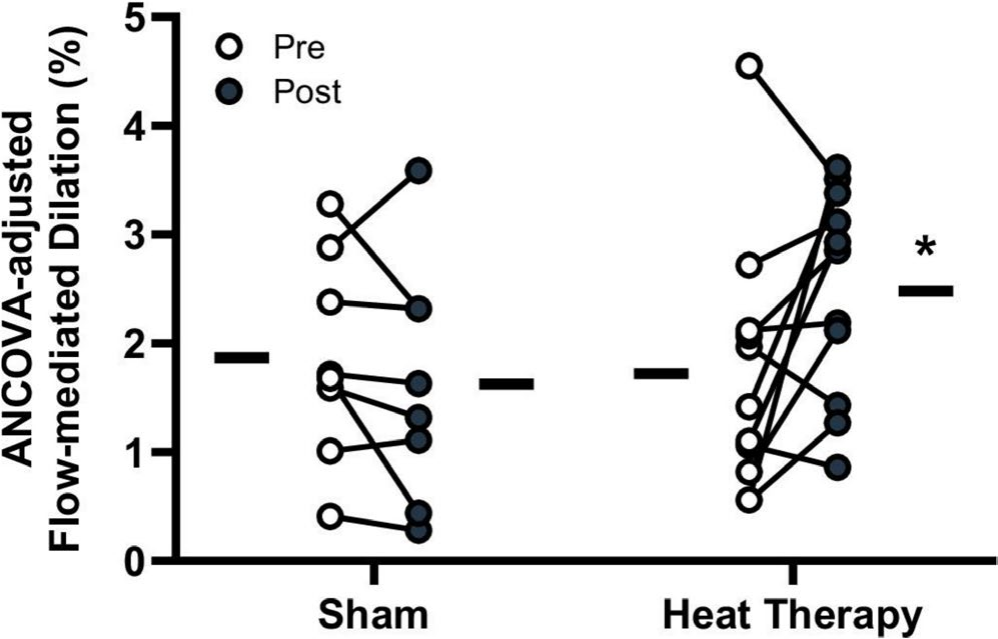
Ruiz‐Pick et al. demonstrated that endothelium‐dependent vasodilation, assessed via flow‐mediated dilation (shown as analysis of covariance (ANCOVA) adjusted flow‐mediated dilation), was improved following 8 weeks of home‐based heat therapy. Data were analyzed using a two‐way (group × time) mixed model analysis of variance with repeated measures (sham, *n* = 8; heat therapy, *n* = 11). Logarithmically transformed baseline diameter and shear rate area under the curve summed through peak diameter were entered as covariates. The horizontal black line denotes the mean at each time point, for each group and is positioned adjacent to the individual data points. *p = 0.02 vs. pre, within group. Figure used with permission. Previously published in the *Journal of Applied Physiology* (Figure 4; Ruiz‐Pick et al. 2025).

Ruiz‐Pick et al. 2025, using a home‐based heat therapy, reported improvements in flow‐mediated dilation in the superficial femoral artery following 8 weeks of heat therapy, but these results were not noted in the sham group, thereby indicating that the improvements were due to heat therapy alone (see Figure 8). Moreover, following nitroglycerin administration, a nitric oxide donor, there were no differences in endothelial‐dependent dilation between groups. These results suggest that the improvements in dilatory function were isolated to the endothelium and not the smooth muscle. Interestingly, there were no changes in reactive hyperemia between either the sham or heat therapy group, suggesting that improvements were in the conduit vessel only and not the downstream microvasculature (Ruiz‐Pick et al. 2025).

### 6.3 Autonomic Health and Function

Several investigations have reported the various effects of acute whole‐body heat exposure on sympathetic activity. Most of these studies have been done as a means of understanding the baroreflex response to heat exposure (Cui et al. 2002a, 2002b, 2022; Crandall et al. 2003; Crandall 2008). As we note, few researchers have investigated the effect of chronic heat exposure on autonomic function. Local acute heat exposure, as used by Engelland et al. (2020), increased muscle sympathetic nerve activity (MSNA) in young adults, but blood pressure and neurovascular transduction were unchanged. Interestingly, endothelin‐1 increased following acute heat exposure, suggesting this vasoactive compound may play a role in the maintenance of blood pressure in this setting (Engelland et al. 2020).

Gayda, Bosquet, et al. (2012) used heart rate variability to assess autonomic function following the combination of acute heat exposure (traditional sauna) and exercise in adults with untreated hypertension. They reported that their participants had significant increases in low‐frequency heart rate variability and reductions in high‐frequency heart rate variability, indicating an increase in sympathetic and a decrease in parasympathetic activity (Gayda, Bosquet, et al. 2012; Houle and Billman 1999; Billman et al. 2015). Conversely, Laukkanen et al. (2019) reported favorable autonomic modulation following an acute traditional sauna session. Notably, there was an increase in high‐frequency variability and a decrease in low‐frequency variability in the 30 min following recovery from sauna bathing. The authors suggest these acute adjustments may underpin long‐term cardiovascular adaptations following chronic sauna use (Laukkanen et al. 2019).

**FIGURE 9:**
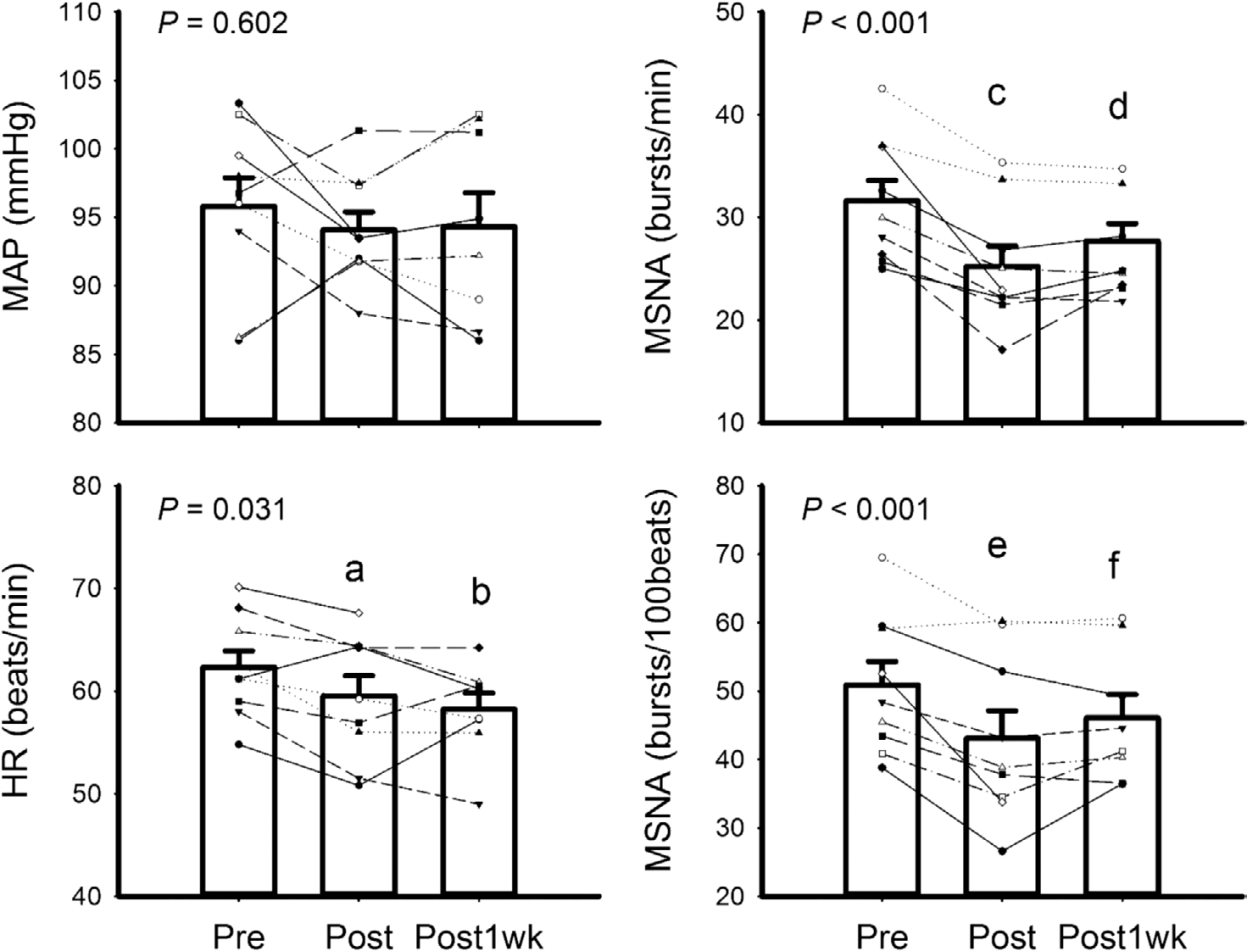
Cui et al. report the reduction in sympathetic activity following 20 warm bath sessions. Mean and individual muscle sympathetic nerve activity (MSNA), mean arterial pressure (MAP), and heart rate (HR) are reported before (Pre), after (Post), and 1 week following the end of the 20 baths (Post1wk). MSNA is reported as burst frequency (bursts/min) and burst incidence (bursts/100 beats). *p* value listed is the result of a one‐way repeated‐measures ANOVA. Post hoc values are reported vs. Pre, ^a^ *p* = 0.040; ^b^ *p* = 0.045; ^c^ *p* < 0.001; ^d^ *p* = 0.001; ^e^ *p* < 0.001; and ^f^ *P* = 0.018. Figure used with permission. Previously published in the *Journal of Applied Physiology* (Figure 1; Cui et al. 2022).

To determine the effects of chronic heat exposure or heat therapy, Cui and colleagues (Cui et al. 2022) used direct nerve recordings and a combination of in‐laboratory and home‐based heat therapy to investigate how heat therapy would change baroreflex sensitivity and the sympathetic responsiveness to stressors (e.g., hand grip, cold pressor test). Participants in their investigation performed 4 weeks of “warm baths” 30 min a day, 5 days per week, at a temperature between 39°C and 41°C (Cui et al. 2022). Neither cardiac nor sympathetic baroreflex sensitivity was changed following heat therapy, nor were the responses to any of the stressors. Interestingly, the operating points of both the cardiac and sympathetic baroreflex were reset to a lower MSNA (see Figure 9) and heart rate (Cui et al. 2022).

Ely, Clayton, McCurdy, et al. (2019) also used direct nerve recordings to investigate the effects of heat therapy in women with PCOS. We will detail this study later in this review, but it is important to mention that MSNA was measured before and after 8–10 weeks of water immersion heat therapy. They reported that burst incidence was reduced by Δ 11 bursts per 100 heartbeats and burst frequency was also reduced by Δ 7 bursts per minute. These data show the powerful impact of heat therapy on sympathetic activity in a population known to have sympathetic overactivity (Ely, Clayton, McCurdy, et al. 2019). Unfortunately, there are limited studies that have investigated the long‐term autonomic adaptations to heat therapy with direct recordings. Thus, more work is needed in this area to better understand the relationship between heat therapy and sympathetic activity.

### 6.4 Blood Pressure

Blood pressure is a highly regulated variable in our bodies. It is this variable that, when chronically elevated, causes an increased risk of several cardiovascular diseases and is the number one risk factor for heart disease. This section will outline the adjustments and adaptations of blood pressure in response to heat therapy in healthy young, middle‐aged, and older adults.

#### 6.4.1 Heat Therapy Adaptations in Blood Pressure in Young and Middle‐Aged Adults

In an investigation performed by Brunt, Howard, et al. (2016), chronic heat exposure via whole‐body hot water immersion reduced mean arterial and diastolic pressure throughout an 8‐week heat exposure. They reported that mean arterial pressure was reduced at 2 weeks and continued to be reduced, along with diastolic pressure, at weeks 4, 6, and 8 following heat therapy (Brunt, Howard, et al. 2016). Conversely, Cheng and colleagues (Cheng et al. 2024) found no reductions in blood pressure in their young adult participants following 8 weeks of local water immersion (ankle and foot) heat therapy. Cheng et al. also had two other intervention groups, one of which was exercise alone and the other was heat therapy plus exercise. Interestingly, there was no effect of either the exercise training alone or the combination of heat therapy and exercise on blood pressure (Cheng et al. 2024).

Acute Bikram yoga sessions have also been shown to elicit reductions in diastolic blood pressure that may potentiate long‐term changes with regular practice (Miranda Hurtado et al. 2019). The translation of adjustments to adaptations for Bikram yoga practitioners has not been as clear‐cut. Tracy et al. (2013) reported that 8 weeks of Bikram yoga did not affect blood pressure, maximal oxygen consumption, or heart rate, but did elicit improvements in body composition and flexibility (Tracy et al. 2013). Similarly, when Tracy and colleagues examined the effects of Bikram yoga on measures of cardiovascular function and disease risk in young, healthy, sedentary individuals, the effects were matched between groups (Tracy and Hart 2013). In contrast, another investigation reported that Bikram yoga did improve blood pressure and flow‐mediated dilation in young black women following sodium loading (Hunter et al. 2023).

Results have also been mixed for Bikram yoga in middle‐aged individuals. Following an 8‐week intervention where a group of middle‐aged experienced and naïve Bikram practitioners completed between 2 and 7 sessions per week, mean arterial pressure was reduced by 4 mmHg (Marger et al. 2016). Reductions in systolic blood pressure were also observed in heat stress‐naïve individuals following the 8‐week intervention (Marger et al. 2016). In a separate 16‐week intervention, middle‐aged participants with a higher adherence rate (3–5 Bikram Yoga sessions per week) had greater reductions in diastolic blood pressure than those who were less adherent (Hewett et al. 2017).

Is the effect of heat therapy on blood pressure equivocal, based on these studies that have had mixed results in young and middle‐aged participants? We would argue that the different heating methods used between studies play a significant role in whether there are beneficial adaptations. The stimulus for adaptation, whole‐body hot water immersion as used by Brunt, Howard, et al. (2016) vs. the ankle and foot bath as used by Cheng et al. (2024), is vastly different. It would also appear that population and training status may be critical modulatory factors when looking at the efficacy of combined heat therapy and yoga exercise. Overall, a knowledge gap still exists regarding the minimal stimulus or heat therapy prescription for reducing blood pressure in healthy young and middle‐aged adults.

#### 6.4.2 Effect of Heat Therapy on Blood Pressure Responses in Healthy Aged Adults

**FIGURE 10:**
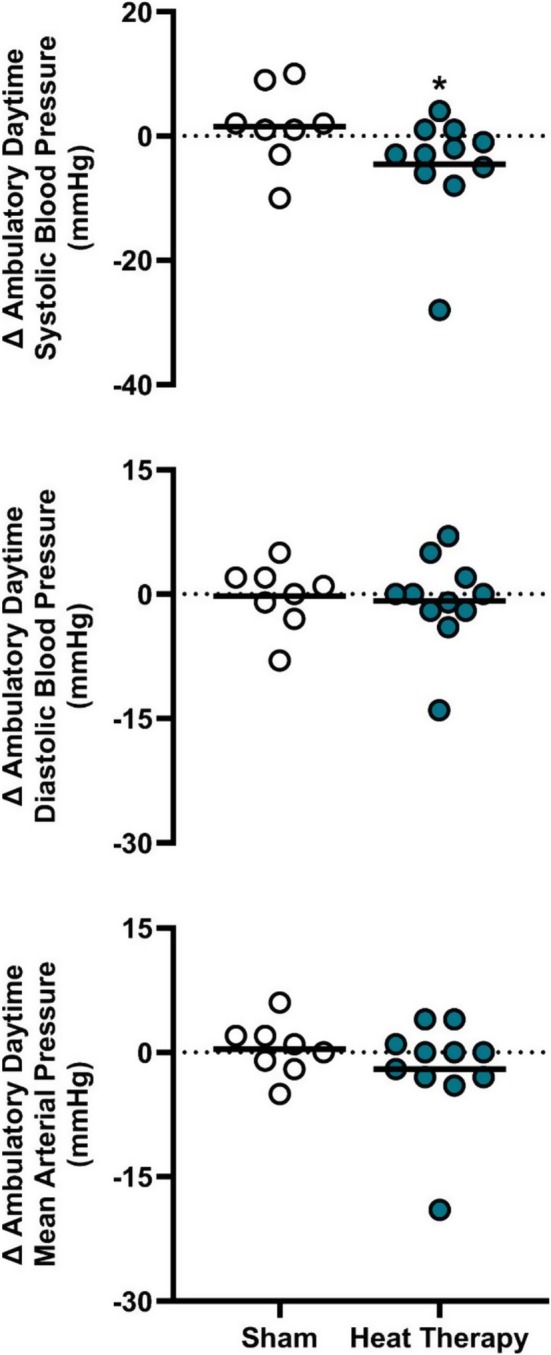
Ruiz‐Pick et al. reported that ambulatory systolic blood pressure (measured via 24‐h blood pressure) was improved following 8 weeks of home‐based heat therapy. The change in ambulatory daytime systolic (top), diastolic (middle), and mean (bottom) arterial blood pressures are shown for each group. The delta pressure was calculated as post‐intervention minus pre‐intervention pressure. Data were analyzed using a one‐tailed Student *t*‐test (sham, *n* = 8; heat therapy, *n* = 11). The horizontal black line denotes the mean. *p = 0.04 vs. sham. Figure used with permission. Previously published in the *Journal of Applied Physiology* (Figure 3; Ruiz‐Pick et al. 2025).

From participants in the Kuopio Ischemic Heart Disease Study, we know that higher frequency sauna use in men aged 42–60 results in a lower risk of developing hypertension (Zaccardi et al. 2017). Brunt, Rosenberg, et al. (2019) also investigated the effect of heat therapy in older adults, using 30 sessions of whole‐body water immersion. Their preliminary results show that systolic blood pressure is reduced by ~10 mmHg. Similarly, Blankenship et al. also saw reductions in mean arterial pressure and diastolic blood pressure in their population of older adults following 4 weeks of whole‐body water immersion heat therapy (Blankenship et al. 2025). An 8‐week home‐based heat therapy program in older adults using water‐perfused pants by Ruiz‐Pick et al. (Figure 10; Ruiz‐Pick et al. 2025) also showed that 24‐h systolic blood pressures were reduced by an average of 5 mmHg in the heat therapy group, and in‐laboratory pressures (systolic, diastolic, and mean) were reduced by 8–9 mmHg (Ruiz‐Pick et al. 2025). In contrast to these strong effects, one report in healthy older adults using far‐infrared sauna by Fuchs et al. (2025) reported no change in blood pressure. Given the limited impact of far‐infrared on core body temperature, as noted previously (Reed et al. 2025), it would appear that, in general, heat therapy is effective at reducing blood pressure and protecting against the development of hypertension in healthy aged adults, so long as appropriate modalities are used.

### 6.5 Benefits of Heat Therapy on the Brain and Neurocognitive Disorders

Alzheimer's disease is the most common neurodegenerative disease, and yet, there are currently no disease‐modifying treatments and no cure. Characterized by worsening memory, reduced social performance, and declines in cognitive function (Nelson et al. 2012; Teri et al. 1989), the etiology of AD is poorly understood. While aerobic exercise has shown some benefits for improving brain health during healthy aging and in dementia models of AD (Adlard et al. 2005; Ahlskog et al. 2011; Intlekofer and Cotman 2013), not all individuals benefit from exercise. Reasons for this include difficulties with compliance or the presence of comorbidities that hinder an individual's ability to complete exercise. As a result, alternative therapies and treatments for the declines in cognitive function that occur with AD and other neurodegenerative diseases are needed. Heat therapy could be a viable alternative.

#### 6.5.1 HSPs as Therapeutic Targets in Brain Health

A loss of cellular protein homeostasis, or proteostasis, is a hallmark of many neurodegenerative diseases (Ciechanover and Kwon 2017; Klaips et al. 2018). AD pathology is characterized by β‐amyloid (Aβ) protein fragments or aggregates and intracellular neurofibrillary tangles of phosphorylated tau (P‐tau) proteins (Sherman and Goldberg 2001; Winklhofer et al. 2008). Aβ in toxic amounts can hinder synaptic communication between neurons and lead to cell death (Winklhofer et al. 2008). Microglia, or immune cells in the brain, are activated to clear Aβ, but failure of this process can lead to chronic and neurodegenerative inflammation (Sherman and Goldberg 2001; Winklhofer et al. 2008; Hands et al. 2008). In addition to toxic protein accumulation, AD pathogenesis is linked to vascular impairments and chronic cerebral hypoperfusion (Akinyemi et al. 2013; de la Torre 2004; Sweeney et al. 2018). The expanding multifactorial etiology of AD includes reduced energy metabolism in the brain, the contribution of an inactive/sedentary lifestyle, reduced aerobic capacity, and cardiovascular and metabolic risk factors (reviewed in Morris et al. 2014). These findings further support the need to pursue alternative novel approaches for the treatment and prevention of AD.

HSPs facilitate many processes known to benefit cellular health, and the upregulation of HSPs as therapeutic targets for the treatment of neurodegenerative disease is an emerging idea (Ciechanover and Kwon 2017; Klaips et al. 2018; Carman et al. 2013; Kalmar et al. 2014; Schapira et al. 2014; Webster et al. 2017). HSPs are integral in the folding of newly synthesized proteins, the refolding of damaged proteins, targeted degradation of nonfunctional proteins and organelles, prevention of oxidative damage, import/export of proteins into/out of the mitochondria, and intracellular signaling (including insulin signaling) (Drew et al. 2014; Willmund et al. 2013; Frydman 2001; Parsell and Lindquist 1993; Hartl et al. 2011). Not surprisingly, changes in the HSP expression profile and cellular localization are linked to numerous disease states. Studies suggest that induction, transcription, and translation of these cytoprotective HSPs decline in chronic metabolic and neurodegenerative diseases (Chung et al. 2008; Di Naso et al. 2015; Labbadia et al. 2011). Amyloid plaques and neurofibrillary tangles are thought to occur as a result of failed protein homeostasis, or proteostasis (Hetz and Saxena 2017; Sherman and Goldberg 2001; Winklhofer et al. 2008; Hands et al. 2008), and age‐dependent declines in HSPs could leave neuronal and non‐neuronal cells open to proteotoxic insults and increase the risk for the development of AD (Hetz and Saxena 2017; Kaushik & Cuervo, 2015).

Activation of the transcription factor heat shock factor 1 (HSF1) initiates the heat shock response in the cytoplasm (Wu 1995). HSF1 is activated in response to elevated temperature, oxidant exposure, metals, and other conditions associated with protein misfolding, perhaps through an internal temperature sensor mechanism that is not yet fully understood (Zhong et al. 1998; Zou et al. 1998). The primary heat shock transcription factor, HSF‐1, is decreased in brains from AD animal models and decreases with increasing severity of neuropathology (Braak staging) in AD brains from human participants (Kim et al. 2017).

There are a number of proteins known to regulate HSF1 that are altered by brain aging. For instance, glycogen synthase kinase 3 (GSK‐3) activity is increased in aging brains and has been known to promote tau pathology (Hooper et al. 2008). Importantly, GSK‐3 is a stress kinase that can also impair insulin signaling and glucose metabolism, and inhibition of GSK‐3 is well known to improve whole‐body insulin resistance and glucose metabolism (Nikoulina et al. 2002) and, as such, is being investigated as a potential AD therapy (Pal et al. 2021). Insulin signaling affects a variety of vital cellular processes within the brain, including amyloid beta trafficking and release, tau phosphorylation, long‐term potentiation, and cell survival (de la Monte 2012; Morris and Burns 2012). Such mechanisms may underscore the increased risk for AD‐related neurodegeneration conferred by insulin resistance. Gupte et al. have shown in preclinical, mechanistic studies that heat therapy directly increases HSP expression and is associated with benefits to fasting glucose and insulin‐stimulated glucose uptake (Gupte et al. 2009, 2011). As mentioned, the benefits of heat therapy likely occur via the induction of molecular chaperones (Calderwood et al. 2009; Hsu et al. 2003), which facilitate the refolding of damaged proteins and the import/export of proteins into and out of the mitochondria, target degradation of nonfunctional proteins/organelles, and prevent oxidative damage (Drew et al. 2014; Parsell and Lindquist 1993; Hartl et al. 2011). Chadwick et al. recently demonstrated that increasing HSPs with repeated bouts of heat therapy in a pilot study of human participants with fibromyalgia and centralized pain (3 sessions/week of 45 min per session for 4 weeks) resulted in consistent trends for decreased expression of multiple inflammatory markers (Chadwick et al. 2025), and a growing body of work suggests inflammation is a potential factor in AD. This finding is supported by preclinical research where pharmacologically induced expression of HSP70 in an AD mouse model decreased inflammatory cytokine levels (Sun et al. 2017). Importantly, cognitive function was also improved, as was clearance of Aβ40 and Aβ42, as a result of geranylgeranylacetone (GGA) induction of HSP70. Administration of recombinant HSP70 has been shown to alter the inflammatory environment in rat brains (Shevtsov et al. 2014) and ameliorate Aβ accumulation and memory deficits in aged mice (Bobkova et al. 2014). In addition, prior research shows that the HSP multichaperone complex formed by HSP70/HSP90 can regulate pathologically modified Tau (Moll et al. 2022). Further studies are needed to determine if posttranslational modifications of Tau, such as phosphorylation and acetylation, could occur in response to heat therapy, thereby linking the modification of Tau by HSPs to this intervention. Together, these findings in humans and in animal models provide strong evidence that targeting the Heat Shock response using heat therapy could be an effective approach for preventing neurological disease.

#### 6.5.2 Evidence for Targeting Peripheral Metabolism to Modulate AD Risk

Elevated fasting glucose is linked with increased clinical progression of cognitive impairment in its early stages (Morris et al. 2014). Glycemic regulation is starting to be recognized as an effector of AD neuropathology in cognitively healthy older adults at risk for AD (elevated cerebral amyloid). This suggests a potential link between glycemic regulation and proteostasis. For instance, prior data show cross‐sectionally that both prediabetic fasting glucose levels and a history of dietary sugar intake track positively with increased regional cerebral amyloid in cognitively healthy older adults (Taylor et al. 2017). The relationship between blood glucose levels and amyloid neuropathology in the earliest stages of AD‐related pathology is further supported by mechanistic work. Increased blood glucose is associated with increased brain interstitial amyloid (Macauley et al. 2015), while in tissues, elevated glucose is associated with inhibited amyloid beta catabolism and increased amyloid beta levels (Akhtar et al. 2016). Finally, secondary analysis of the Alzheimer's Prevention through Exercise (APEX) trial (*n* = 106) in cognitively healthy older adults at risk for AD shows that longitudinal increases in fasting glucose over 1 year are associated with regional increases in brain amyloid regardless of exercise treatment group (Honea et al. 2022). This suggests that glucose regulation may be an important therapeutic target in cognitively healthy individuals with AD risk factors.

In this same secondary analysis of the APEX trial, the initial signs of cerebral AD‐related brain changes occurred across multiple modalities, including MRI measures of brain structure, which occurred in the absence of significant differences in body weight, body composition, or apolipoprotein epsilon 4 (APOE4) genotype status (carrier or noncarrier). Specifically, increased levels of hippocampal atrophy were seen in individuals whose glucose worsened longitudinally compared to those whose glucose improved (Honea et al. 2022). Together, these data demonstrate the importance of targeting systemic glucose metabolism as a potential way to modulate brain health outcomes in cognitively healthy older adults at risk for AD.

Insulin signaling can alter Aβ trafficking and release, tau phosphorylation, and cell survival, processes that increase risk for neurodegeneration (de la Monte 2012; Morris and Burns 2012). Increased circulating levels of insulin or systemic insulin resistance have been shown to alter the blood–brain barrier by downregulating endothelial insulin receptors and decreasing permeability of the blood–brain barrier to insulin (Rhea and Banks 2019). Morris et al. (2016) have also shown that insulin sensitivity is impaired in patients with AD. Using the hyperinsulinemic‐euglycemic clamp, they showed that patients with AD had decreased insulin sensitivity compared to cognitively healthy older adults. Given that skeletal muscle is the site of 80%–85% of glucose disposal (DeFronzo and Tripathy 2009), impaired skeletal muscle metabolism could greatly impact AD risk. Low aerobic capacity is a risk factor for AD, and skeletal muscle mitochondrial content and function drive whole‐body aerobic capacity (Lanza and Nair 2009; Tanaka and Seals 2008). Skeletal muscle mitochondrial dysfunction has been examined as a primary cause of whole‐body insulin resistance (Fisher‐Wellman and Neufer 2012; Lee et al. 2009; Yuzefovych et al. 2012), which contributes to increased AD risk (Arvanitakis et al. 2004; Cheng et al. 2011; Janson et al. 2004; Leibson et al. 1997; Luchsinger et al. 2007; Ott et al. 1999; Peila et al. 2002; Profenno et al. 2010; Stewart and Liolitsa 1999; van der Heide et al. 2006; Xu et al. 2009; Yaffe et al. 2004).

Extensive research demonstrates that mitochondria from participants with AD are different from mitochondria from participants without cognitive dysfunction (Swerdlow 2018). These differences are observed systemically, as individuals with mild cognitive impairment (MCI) or AD show decreased cytochrome oxidase activity in blood platelets and lower mitochondrial respiratory rates in cell lines generated from mitochondrial DNA from patients with AD compared with those generated from cognitively healthy older adults (Silva et al. 2013). The production of reactive oxygen species (ROS), or mitochondria‐produced H_2_O_2_ emissions, results in increased Aβ production that can lead to mitochondrial deficits (Silva et al. 2013; Parker Jr et al. 1990). Mice transgenic for the human APOE4 gene, the primary genetic risk factor for AD, have decreased mitochondrial respiratory capacity and reduced electron transport complex content in neurons (Chen et al. 1999). In human skeletal muscle, a relationship between cellular stress in skeletal muscle and cognitive status in APOE4 carriers has been demonstrated (Johnson et al. 2023). These additional results suggest that genetic risk may mediate early‐life effects on skeletal muscle mitochondria and energy expenditure and impact AD risk (Johnson et al. 2025).

Another aspect of mitochondrial function implicated in AD involves quality control processes, or the regulation of mitochondrial biogenesis and mitophagy (the degradation of mitochondria via autophagy) (Ni et al. 2015). The process of targeting mitochondria to lysosomes to be degraded or recycled helps maintain a healthy mitochondrial pool and thus maintains neuronal function and survival (Fang et al. 2016, 2014). It is a plausible theory that a defect in mitophagy results in the accumulation of dysfunctional mitochondria, further promoting AD pathology and memory loss. Although no studies to date have examined the impact of heat therapy on mitophagy function in humans, future work will hopefully address this potential mechanism of action.

#### 6.5.3 Evidence for Targeting Vascular Function to Modulate AD Risk

Cardiovascular disease contributes to increased risk of AD and vascular dementia, a form of dementia associated with cerebrovascular disease (Kennelly et al. 2009; Ying et al. 2016). Evidence supports a link between AD and reductions in cerebral blood flow and alterations to the blood–brain barrier (Akinyemi et al. 2013; de la Torre 2004; Sweeney et al. 2018). The blood–brain barrier is integral for Aβ clearance, the regulation of macrophage infiltration, and inflammation in general (Zlokovic 2011), and HSPs have emerged as key mediators in maintaining blood–brain barrier integrity (Jiang et al. 2020). Although prior research has focused on HSP function in cerebral ischemia–reperfusion injury, the potential for HSPs to reduce blood–brain barrier permeability by minimizing oxidative stress and suppressing inflammation offers promising therapeutic strategies for AD treatment as well. Hypertension, prevalent in two‐thirds of adults over the age of 65, has also been tied to the development of cognitive decline, AD, and vascular dementia. For example, higher systolic blood pressure is associated with smaller regional and total brain volume as well as decreased brain volume over time (Firbank et al. 2007; Gianaros et al. 2006; Glodzik et al. 2012; Leritz et al. 2011; Nagai et al. 2008). The brains from individuals with chronic hypertension demonstrate increased Aβ, atrophy, and neurofibrillary tangles, as well as evidence of decreased brain glucose metabolism (Ashby et al. 2016; Petrovitch et al. 2000). Vascular remodeling and increased arterial stiffness can lead to increased arterial pulse pressure and resultant endothelial dysfunction. Furthermore, as a result of hypertension, endothelial dysfunction occurs with chronic decreases in cerebral blood flow. Importantly, recent findings in healthy older adults demonstrate a relationship between cerebrovascular health and AD neuropathology, emphasizing the potential for vascular dysfunction in the development of neurodegenerative disease (Liu et al. 2019; Sisante et al. 2019). All of these factors emphasize the important role of studying vascular function in AD progression and prevention.

#### 6.5.4 Potential Benefits of Heat Therapy for AD Prevention

As discussed in an earlier section on heat therapy and metabolism, numerous studies demonstrate heat therapy benefits for blood glucose regulation. Despite a lack of data in AD risk cohorts, a recent study demonstrated the safety and effectiveness of heat therapy in aged individuals with peripheral arterial disease. In that 12‐week study, heat therapy via hot water immersion and supervised exercise improved walking distance and resting blood pressure (Akerman et al. 2019). This study demonstrates that heat therapy improves functional ability and cardiovascular outcomes in aged individuals (mean age of heat group 76 ± 8 years). Importantly, adherence to heat therapy was excellent, and the heat was well‐tolerated in this population. Chronic heat therapy has been shown to improve cardiovascular outcomes in a number of populations. Eight weeks of heat therapy improved endothelial function, arterial stiffness, wall thickness, and blood pressure in healthy, young individuals (Brunt, Howard, et al. 2016), and improved cardiovascular outcomes in individuals with type 2 diabetes (James et al. 2024), and PCOS (Ely, Francisco, et al. 2019).

Blankenship and colleagues conducted the first study to examine the effect of heat therapy on AD risk. The authors conducted a pilot study of 4 weeks of heat therapy (3 sessions/week with an increase in core temperature of 1°C throughout a 45‐min heat session) in older individuals at risk for AD (Blankenship et al. 2025). A total of 18 participants (9 males, 9 females; mean age: 71.1 ± 3.9 years) demonstrating metabolic risk (i.e., a diagnosis of prediabetes or T2D, or at least two of the following criteria: hypertension, dyslipidemia, BMI ≥ 30) completed the intervention. Participant adherence for the study was 96% (8 missed sessions out of 216 total sessions), with one study‐related mild adverse event (mild dizziness/nausea). Overall, the research participants responded to a post‐intervention survey saying they enjoyed participating in the study and it was not a burden on their schedules. Secondary outcomes of the intervention demonstrated significant changes in mean arterial pressure, diastolic blood pressure, and cerebral blood flow (*p* < 0.05), with a trend toward improved body mass index (*p* = 0.06). Although no changes were observed in fasting glucose or HbA1c, additional studies are underway with a longer intervention (10 weeks) and a thermoneutral control group.

Richey et al. (2025) investigated the impact of 8 weeks of home‐based lower leg heat therapy on plasma biomarkers of Alzheimer's disease and related dementias in postmenopausal women with hypertension and no cognitive impairment. In the participants who underwent 8 weeks of heat therapy, plasma phosphorylated Tau 181 (p‐Tau181) was reduced. These findings may indicate that although hypertension increases the risk of Alzheimer's disease and related dementias, heat therapy may serve as protection for neurocognitive decline (Richey et al. 2025).

**FIGURE 11:**
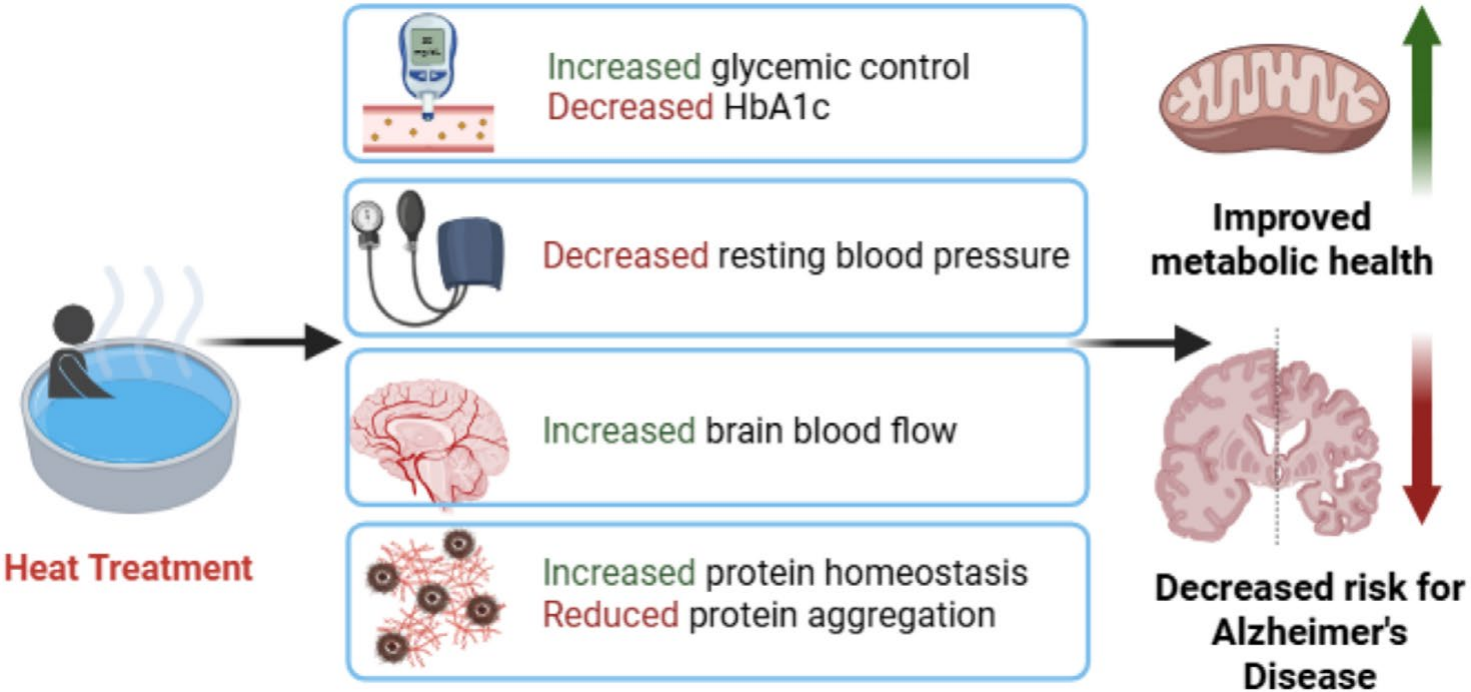
Overview of the potential impact of heat treatment on brain health. Repeated heat treatment, primarily by hot water immersion, results in several systemic effects with implications for brain health including increased glycemic control and decreased resting blood pressure. Pilot studies reveal, for the first time, alterations in brain blood flow with chronic heat treatment, and preclinical studies demonstrate increased protein homeostasis with implications for total protein aggregation. Together these physiological adaptations could improve metabolic health and decrease the risk for Alzheimer's Disease. HbA1c, hemoglobin A1c, or glycated hemoglobin. *Created with Biorender.*

Like heat therapy, sauna bathing has also been shown to have beneficial effects on cardiovascular disease (CVD) and dementia (Heinonen & Laukkanen, 2018). We have previously described the prospective population‐based study in which sauna bathing was associated with a significantly lower risk of fatal CVD‐related events and all‐cause mortality (Laukkanen et al. 2015). A follow‐up study on that same cohort further showed that sauna bathing lowered the risk of neurodegenerative disease, dementia, and AD (Laukkanen et al. 2017). The findings indicate that sauna bathing four to seven times a week results in a risk reduction of 66% for dementia and a 65% risk reduction of AD when compared with only one sauna session per week. Similar to hot water immersion, sauna bathing has also been shown to produce systemic blood pressure‐lowering effects, increase cardiac output via an increase in heart rate, and decrease peripheral vascular resistance. Further studies are warranted to establish the potential mechanisms linking sauna bathing and memory diseases but support the idea that interventions that elevate body temperature may improve peripheral metabolic function and brain health, possibly by activating the HSP response. Figure 11 shows a summary of the beneficial effects of heat therapy on AD and dementia risk.

And finally, although studies directly examining the effects of heat therapy on mitochondrial function in patients at risk of AD have not been conducted, existing studies in humans are promising. The use of diathermy in healthy, young adults has demonstrated improved skeletal muscle mitochondrial function when used for several hours a day for 6 days (Hafen et al. 2018) up to 6 weeks (Marchant et al. 2022). These findings support the extensive evidence from preclinical studies demonstrating an effect of heat therapy on mitochondrial function (Liu and Brooks 2012; Henstridge et al. 2014; Drew et al. 2014; Archer et al. 2018; Von Schulze et al. 2021). Given the growing emphasis on investigating mitochondrial health in aging and neurodegenerative disease, this is an exciting future direction for studies on heat therapy. Overall, research suggests that the many biological functions of HSPs to modify membrane structure, proteostasis, inflammation, cell signaling, and mitochondrial function, among others, could benefit patients with AD and other neurological disorders.

### 6.6 Heat Therapy as an Alternative Treatment for Mental Health and Sleep Quality

#### 6.6.1 Changes in Mental Health Following Heat Therapy

We have previously discussed several investigations that were aimed at using heat therapy as a nonpharmacological intervention to improve physiological health. Mental health (e.g., depression, anxiety, etc.) has a significant impact on cardiovascular function, autonomic function, muscular function, metabolic function, cognition, disease risk, and disease (Auerbach et al. 2018; Dewani et al. 2023; Phimphasone‐Brady et al. 2024; ter Meulen et al. 2021; Walker et al. 2015). Therefore, it is important to determine whether heat therapy can be an alternative or adjuvant treatment for various mental health disorders.

There are some reports that acute heat exposure improves various indices of mental health. Bauer et al. (1995) reported that in the immediate day following a fever, patients with major depressive disorder had an improvement in mood, but unfortunately, their mood worsened in the subsequent days. These results may be because acute improvements due to increased body core temperature are transient, or the lack of a fever the next day could directly impact their mood (Bauer et al. 1995). Janssen et al. (2016) investigated the impact of an acute heat exposure session on depression severity. Participants were randomly assigned to either a sham group or a whole‐body heat exposure group and underwent assessments of various indices of depression and mental health using the Hamilton Depression Rating Scale, Inventory of Depressive Symptomatology self‐report, Sheehan Disability Scale, and Quality of Life Enjoyment Satisfaction Scale (Janssen et al. 2016). They also reported the length of their current depressive episode and the number of past episodes before and after acute heat exposure. The whole‐body heat exposure group underwent mild‐intensity hyperthermia where the heating was focused at the level of the chest through infrared lights, and at the level of the legs through infrared heating coils. Participants were heated until their core temperature reached 38.5°C, which took between 81 and 140 min. Following one bout of acute heat exposure, measurements were taken across 6 weeks at various weekly and biweekly intervals. Janssen et al. reported that depression scores were lower in the 6 weeks following acute heat exposure. Furthermore, the reduction in symptom severity and depressive episode remission rivals that of antidepressant trials which involve daily pharmacological intervention (Janssen et al. 2016). Given the promising nature of these data, others have followed similar investigations in a variety of populations with various health conditions and diseases.

Laukkanen and colleagues prospectively analyzed a cohort of men aged 42–61 years to determine the impact that Finnish sauna bathing had on psychotic disorders (Laukkanen, Laukkanen, and Kunutsor 2018). Those who performed sauna bathing more frequently during the week had a lower risk of developing psychotic disorders than those who performed fewer sauna bathing sessions. These results remained even after excluding men who were taking antidepressants and adjusting for age, body mass index, total cholesterol, socioeconomic status, and physical activity (Laukkanen, Laukkanen, and Kunutsor 2018). Waon therapy has also been used as a means of improving mental health (Soejima et al. 2015; Masuda et al. 2005). Soejima and colleagues (Soejima et al. 2015) tested the feasibility and safety of Waon therapy in patients with chronic fatigue syndrome. As part of this study, they administered the Profile of Mood States or POMS questionnaire before and after 4 weeks of 5 days per week Waon therapy. They reported that the POMS score for anxiety and depression was decreased following heat therapy (Soejima et al. 2015).

Similarly, Masuda et al. (2005) also used Waon therapy in a group of mildly depressed patients. Heat therapy reduced somatic and mental complaints but did not change the self‐rating depression scale results (Masuda et al. 2005). Mason and colleagues investigated the effect of either 4 or 8 heat therapy sessions in combination with a maximum of 8 cognitive behavioral therapy sessions (in a week) on individuals with major depressive disorder (Mason et al. 2024). Heat therapy sessions were completed in an infrared sauna with a temperature that reached 52.7°C, and participants stayed in the sauna until body core temperature reached 38.5°C, which ranged from 110 to 140 min between participants. This was then followed by wrapping the participant in warm towels for 15 ± 8 min. Before and after the intervention, depression scores, mood, and cognitive responses were measured. Participants who completed the treatment had reductions in depression symptoms. Additionally, at the end of the intervention, almost all of the participants no longer met the criteria for major depressive disorder (Mason et al. 2024) These data suggest there is great promise in utilizing whole‐body heat therapy as an alternative or adjuvant treatment for those with mental disorders.

Taken together, this limited research indicates that heat therapy generally has a beneficial effect on major depressive disorders and depression symptoms in a variety of populations. We again reiterate that modality, frequency, duration, and temperature are crucial aspects that should be considered and further investigated before a “prescription” is given to adults with mental health disorders or concerns.

#### 6.6.2 Sleep Quality Improvements Following Heat Therapy

Among the lay community, heat therapy has long been associated with beneficial effects on sleep quality, to the extent that “improving sleep” is identified by 80% of sauna bathers as one of their motivations for participating in the practice (Hussain et al. 2019). Multiple large surveys report that sauna users have more satisfactory sleep patterns compared to nonsauna bathers (Engstrom et al. 2024) or have improved sleep quality for several nights following sauna use (Hussain et al. 2019). While much of the data on sleep and sauna bathing is from subjective surveys, there is one classic report from a prospective study in five participants showing that acute sauna use increased the amount of deep (slow‐wave) sleep and reduced the time spent awake during the night (Putkonen and Eloma 1976). More rigorous studies have been conducted on the effects of foot and partial or whole‐body warm baths taken before sleep. Systematic reviews indicate positive effects in most of these studies, including increased slow‐wave sleep and decreased sleep latency (Liao 2002; Nasiri et al. 2024). As these effects are not observed with thermal‐neutral baths (Horne and Reid 1985), this is not a matter of time spent relaxing before sleep but is believed to be an effect on thermoregulatory pathways that link to sleep (Buguet 2007). With so little data directly tracking sleep (i.e., using polysomnography or actigraphy) in relation to heat therapy, and a growing knowledge base around the chronobiology of exercise (Brito et al. 2022), it would seem that a similar exploration of the chronobiology of heat therapy is due.

As mental health and sleep are linked (Baranwal et al. 2023), and the association between AD and sleep disturbance is well‐established (Tranah et al. 2011; Mander 2013), it would seem that this is an area that should be further investigated as the work of heat therapy moves forward. A potential avenue for research could include investigations into heat therapy and sleep quality, and the subsequent changes in autonomic function, cognitive function, and mental health. Regardless of the focus of the investigation, more research is necessary to understand whether heat therapy can be used to improve these aspects of health and well‐being.

## 7 Heat Therapy in Disease Prevention and Management

Heat therapy in young and older healthy adult populations has provided a foundational understanding of the physiological responses to heat therapy. The clinical relevance of heat therapy has also been investigated in some important patient populations. These studies provide more evidence of the clinical utility of heat therapy as an alternative or adjuvant treatment option. In this section, we have outlined several disease conditions and the impact of a heat‐adapted phenotype on clinically meaningful outcomes. The relevant studies have utilized multiple heating modalities and variations on the FTDM principle. Where appropriate, we have outlined these studies in the text, and they are also included in Tables 1, 2, 3.

### 7.1 Impact of Heat Therapy on Metabolic Function in Type 2 Diabetes

Type 2 diabetes continues to be an advancing problem in modern society. An aging population, increasingly sedentary lifestyles, and abundant fast food contribute to this expanding risk. Type 2 diabetes is characterized by metabolic dysfunction, primarily attributed to a loss of insulin sensitivity in peripheral tissues, resulting in poor glucose control. Disease pathology is also closely associated with inflammation, cellular oxidative stress, and mitochondrial dysfunction. Pharmacological approaches, as well as lifestyle modifications like diet and exercise, can be effective in preventing and even reversing type 2 diabetes in its early stages. Adherence to exercise training and diet has long been problematic for many populations; therefore, alternative approaches are in high demand. Although human studies are still somewhat limited, heat therapy in the form of hot water immersion or sauna is effective at targeting the cardiometabolic risk factors of Type 2 diabetes.

An initial study in the field was a small intervention study that investigated the impact of hot tub immersion on eight participants (37.5% female, 43–68 years). Participants underwent six sessions of heat therapy per week for 3 weeks. Heat therapy was administered via hot water immersion for 30 min per session, resulting in a 0.8°C rise in body core temperature. Study participants were all characterized as having type 2 diabetes. After 3 weeks, significant reductions in fasting glucose and hemoglobin A1C (HbA1c, an indicator of long‐term glucose regulation) were observed (Hooper, 1999). Studies by Oláh et al. (2011) and Koçak et al. (2020) employed a similar approach using balneotherapy for 3 weeks. In these studies, glucose levels were decreased after treatment in participants with obesity (BMI > 25 kg/m^2^, Oláh et al. 2011) and type 2 diabetes (Koçak et al. 2020). In fact, in as little as 2 weeks of hot water immersion, Hoekstra et al. (2018) observed decreased fasting glucose in overweight men, while 2 weeks of daily sauna treatment resulted in lowered fasting glucose in healthy obese individuals (Biro et al. 2003). In a series of 8–10 hot water immersion sessions over 14 days, fasting insulin and insulin sensitivity were improved in individuals with type 2 diabetes, though fasting glucose was unchanged (James et al. 2023).

Longer durations of heat therapy also show reductions in blood glucose. Hesketh et al. (2019) measured a decrease in glucose area under the curve (AUC) following an oral glucose tolerance test in 10 healthy, sedentary participants exposed to daily room air at 40°C, with 40% humidity, compared to an exercise‐only group exercising at moderate intensity (40–50 min of cycling at 65% VO_2peak_). Both intervention groups were matched for time, with participants either cycling or exposed to heat for 40–50 min three times per week. As in most of these studies, the comparison group was a nonintervention (i.e., no heat therapy treatment) control rather than a thermoneutral treated group. Nonetheless, the lack of thermoneutral controls in these studies is one limitation of the work to date characterizing the effects of chronic heat on glucose regulation.

Despite the accumulating evidence that heat therapy improves glucose regulation (Hoekstra et al. 2018; Hooper, 1999b; Oláh et al. 2011; Koçak et al. 2020), some studies show no effect of repeated heat bouts on glucose regulation. For example, in a randomized clinical trial of 128 participants with varying degrees of stenosis, Qiu et al. (2014) observed no changes in fasting glucose or 2 h‐post glucose ingestion following 4 weeks of hot water immersion. One possible reason for the lack of effect could be the older population (average age 62–65 years). Also, body core temperature was not assessed; therefore, it may not have been significantly raised by the intervention (water temperature maintained at 39°C). Variability in the timing and length of heat interventions, lack of true control, or monitoring of body core temperature, as well as major differences in participant disease profile (i.e., healthy, Type 2 diabetes, obese, cardiovascular disease, and average age) all result in less than perfect comparisons between studies.

Interestingly, Blankenship and colleagues reported that older individuals with metabolic risk who underwent 4 weeks of heat therapy (three times per week, 45 min per session) exhibited no significant changes in fasting glucose or glucose area under the curve following an OGTT (Blankenship et al. 2025).

**FIGURE 12:**
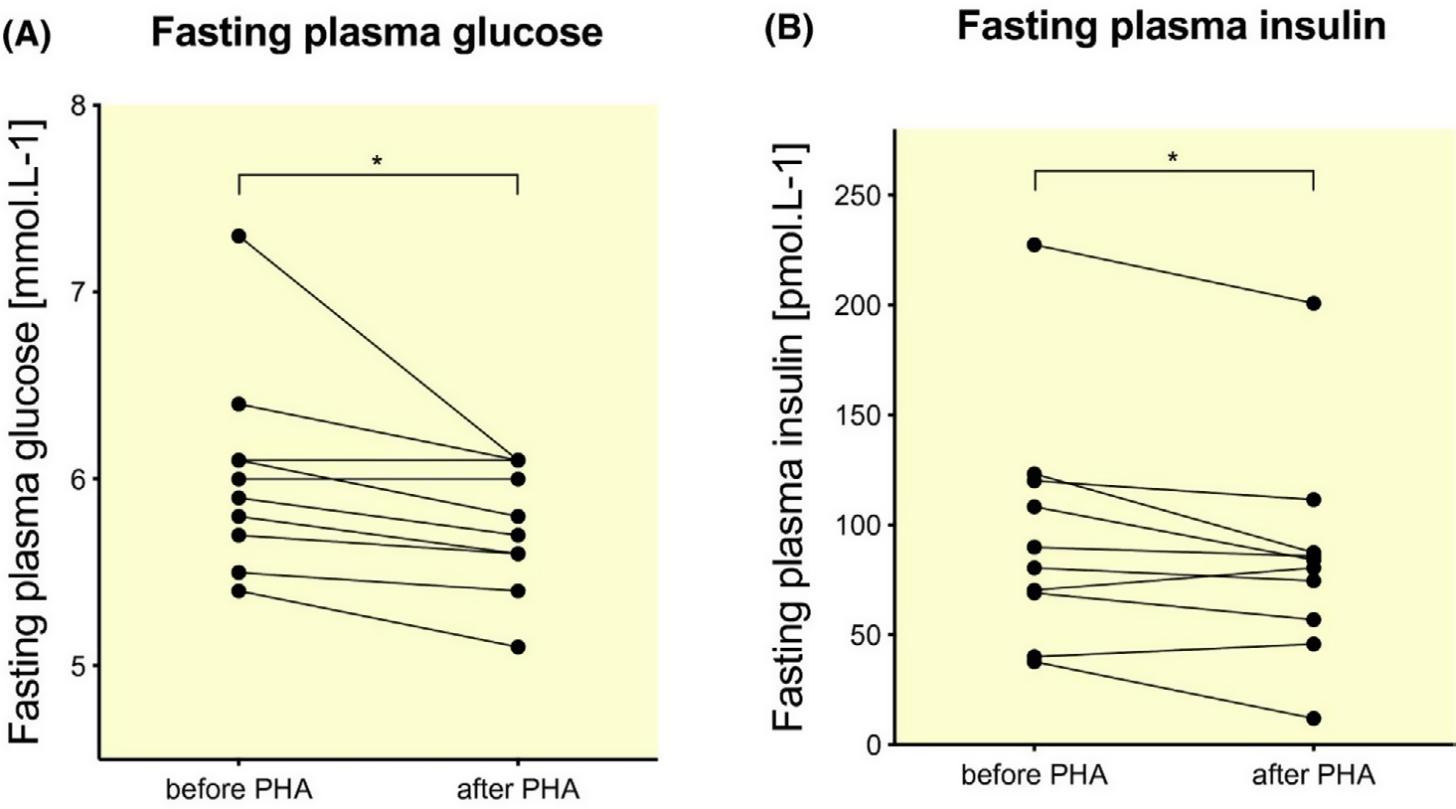
Pallubinsky et al. reported the impact of heat therapy or, as termed in their work, passive heat acclimation (PHA), on fasting plasma glucose (A) and fasting plasma insulin (B) concentrations in overweight humans who are nondiabetic. Data are presented for each individual participant before (pre) and after (post) PHA (*N* = 11). * denotes *p* < 0.05 for pre‐PHA versus post‐PHA. Figure used with permission. Previously published in *Acta Physiologica* (Figure 2; Pallubinsky et al. 2020).

Another study by Pallubinsky et al. (2020; Figure 12) demonstrated that 10 days of repeated heat sessions in overweight, nondiabetic participants resulted in lowered fasting glucose and insulin concentrations following hyperinsulinemic‐euglycemic clamp. Although the length of the study was only 10 days, the heat intervention was 4–6 h/day at a lower heat threshold of 34.4°C. This highlights the need for additional studies to determine the optimal timing, duration, and temperature of heat therapy for metabolic health.

Overall, heat therapy seems to elicit beneficial adaptations in those with Type 2 diabetes, further reinforced by a meta‐analysis published by Sebők et al. (2021). Based on these data, we can speculate that whole‐body heat exposure at temperatures of 39°C–40°C for at least 8 weeks will elicit the greatest benefit in this population.

### 7.2 Impact of Heat Therapy on Hypertension

**FIGURE 13:**
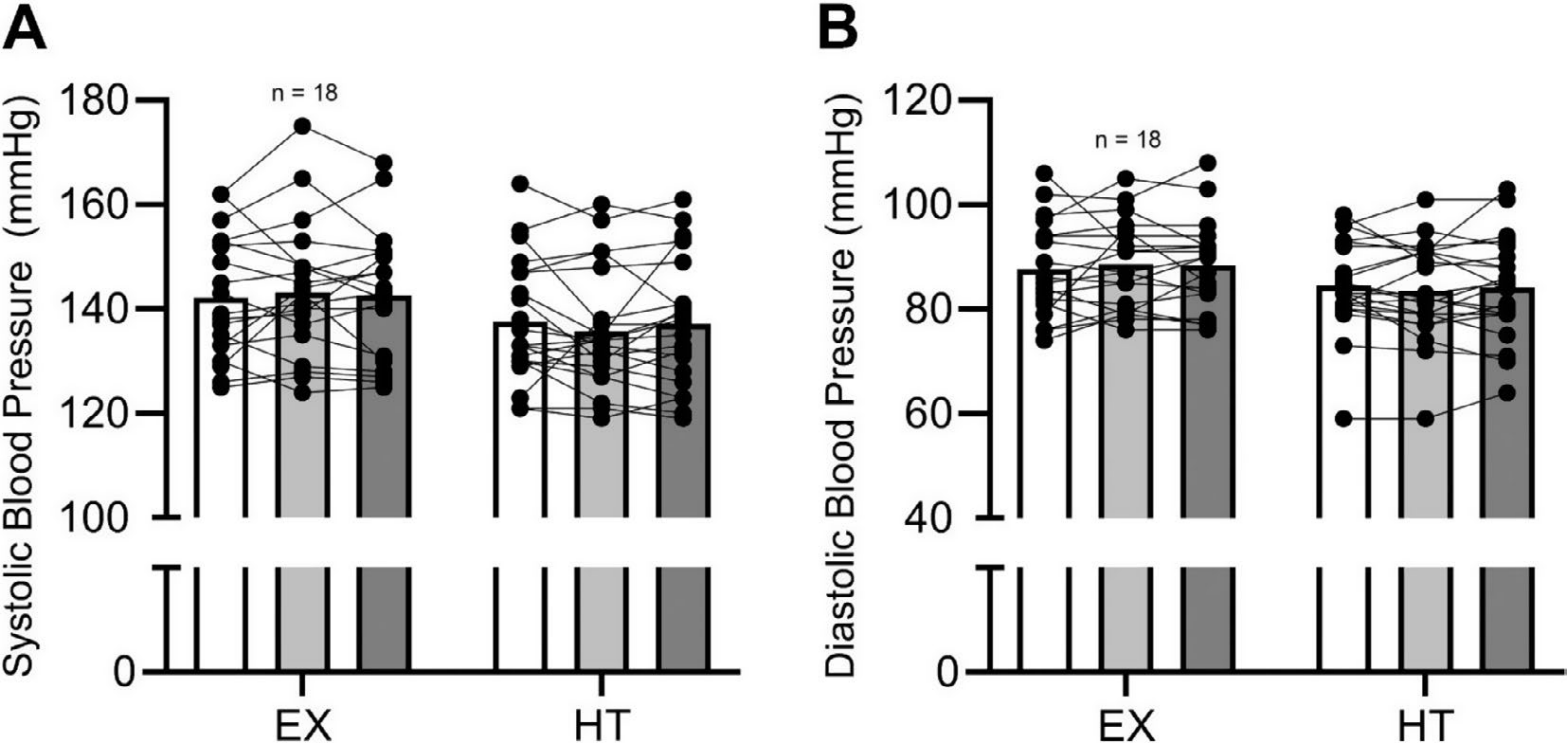
Kaiser et al. reported 24‐h systolic (A) and diastolic blood pressure (B) for the aerobic exercise (EX) and heat therapy (HT) groups before (PRE; white), during (MID; light gray), and after (POST; dark gray) the interventions. Data are presented as group means and individual responses. Aerobic exercise *n* = 19, heat therapy *n* = 21, except as noted in the figure. Figure used with permission. Previously published in the *Journal of Applied Physiology* (Figure 2; Kaiser et al. 2025).

Hypertension is a primary, modifiable risk factor for cardiovascular disease (Chobanian et al. 2003; Flack and Adekola 2020). Although pharmacological treatments are prevalent, the first‐line treatment for hypertension is lifestyle modification, such as exercise training (Whelton et al. 2017). Both exercise training and pharmacological treatments can be, but are not always, effective or adhered to by individuals with hypertension (Whelton et al. 2017; Dickinson et al. 2006). Thus, heat therapy provides an alternative or adjuvant treatment to improve blood pressure in this population. Along these lines, whole‐body heat therapy in the form of Finnish sauna reduced the risk of hypertension development in men who did not have hypertension at baseline testing (Zaccardi et al. 2017). These data, while compelling, are beyond the ability of investigators to replicate in an experimental setting given the considerable duration (mean follow‐up 24.7 years). To build upon these data and investigate whether whole‐body heat therapy or exercise improves hypertension for individuals who currently have hypertension, members of our group (Kaiser et al. 2025) tested the hypothesis that whole‐body heat therapy would reduce blood pressure to a greater extent than exercise in unmedicated men and women with Stage 1 and Stage 2 hypertension. In the randomized clinical trial, Kaiser et al. measured 24‐h blood pressure and arterial stiffness (via pulse wave velocity), before and after 30 sessions of either aerobic exercise or whole‐body hot water immersion. They reported that neither intervention improved 24‐h blood pressure or arterial stiffness in this population (see Figure 13; Kaiser et al. 2025).

Although whole‐body heat therapy elicits the greatest adaptations, as we have previously discussed, some individuals may find it uncomfortable, and in some populations, it may be contraindicated. Therefore, other members of our group investigated whether a home‐based heat therapy could reduce blood pressure and improve cutaneous vascular function, autonomic function, and cardiovascular function in postmenopausal women with hypertension (Richey, Hemingway, et al. 2024; Akins et al. 2024). They hypothesized that following 8 weeks of heat therapy, postmenopausal women with hypertension would have a reduction in 24‐h blood pressure and improvements in cutaneous vascular function. To test their hypothesis, postmenopausal women with hypertension underwent 24‐h blood pressure monitoring and assessments of cutaneous vascular function and neural‐cardiovascular responses to orthostasis before and after 8 weeks of home‐based lower leg heat therapy. Participants were randomized into either a sham or heat therapy group following pretesting. Cutaneous microvascular function via cutaneous microdialysis was measured during local heating, and muscle sympathetic activity and cardiac output were measured during orthostasis. Based on their findings, home‐based heat therapy in the form of lower leg water immersion did not improve 24‐h blood pressure, cutaneous vascular function, or neural‐cardiovascular responses during orthostasis in postmenopausal women with hypertension (Richey, Hemingway, et al. 2024; Akins et al. 2024).

The results of these studies suggest that heat therapy may not improve physiological indices of cardiovascular health for individuals with hypertension. However, this seems to only apply to those with primary hypertension, as heat therapy does seem to improve blood pressure in other populations with cardiovascular disease or cardiovascular disease risk (see PCOS section below). As we delve into the potential reasons why heat therapy did not elicit any adaptations, let us first consider the FTDM (frequency, temperature, duration, modality) principle in the context of experimental design for both groups. First, Kaiser and colleagues utilized whole‐body heat therapy where participants were immersed to mid sternum, while Richey and colleagues and Akins and colleagues had their participants immerse only their lower legs. Each group used water temperatures of 40°C and 42°C (Kaiser et al. and Richey et al., respectively) for the same duration of 45 min (Richey, Hemingway, et al. 2024; Kaiser et al. 2025). One potential reason this therapy may not have been effective is that the duration might not have been long enough to elicit adaptations. Previous reviews of heat therapy have suggested that cumulative heating load may influence the effectiveness of the intervention (Rodrigues et al. 2025). Pursuant to the FTDM principle, greater frequency or duration of heating may be required to reach the requisite heating load needed to improve blood pressure in adults with primary hypertension. Previous studies of heat therapy, per se, that have elicited reductions in blood pressure have utilized greater heating durations, frequencies, or both, irrespective of modality (Brunt, Howard, et al. 2016; Ely, Clayton, McCurdy, et al. 2019; Ruiz‐Pick et al. 2025). Hypertension has a highly diverse etiology, and it is likely that there is an aspect of blood pressure regulation that remains to be understood in terms of primary hypertension. It is possible that this aspect remains impervious to the effects of heat therapy, particularly during commonly employed intervention lengths (8–12 weeks). Although there is a meta‐analysis that evaluated the effects of heat therapy on blood pressure and vascular function, this did not include studies specifically investigating hypertension (Pizzey et al. 2021); thus, we cannot extend their conclusions to populations with hypertension. Furthermore, it is still unclear what the FTDM recommendation would be for adults with hypertension. Additional investigations of heat therapy for lowering blood pressure in adults with primary hypertension that leverage different permutations of the FTDM principle will help inform researchers and clinicians as to how heat therapy can be most effectively utilized in this population.

### 7.3 Coronary Risk Factors, Coronary Artery Disease, and Chronic Heart Failure

Heat therapy research in adults with heart disease (i.e., coronary risk factors, coronary artery disease, and chronic heart failure) has primarily used the modality of Waon therapy (Kihara et al. 2009; Kuwahata et al. 2011; Miyata et al. 2008; Miyata and Tei 2010; Shinsato et al. 2010; Tei et al. 2007; Fujita et al. 2011; Ohori et al. 2012). The improvements in cardiovascular health are impressive, although not consistent across other heating modalities. In this section, we will describe the results of the various research studies that have been conducted in populations with heart disease and their results.

Kuwahata et al. (2011) investigated adaptations in autonomic function for patients with chronic heart failure that occur following Waon therapy. Patients were asked to complete 4 weeks of 1 session per day (5 days a week) of Waon therapy and were instructed to continue taking their prescribed medications. They reported that following heat therapy, high‐frequency heart rate variability was increased and low‐frequency heart rate variability was decreased, suggesting that in their sample of patients, heat therapy increased parasympathetic activity and reduced sympathetic activity at the heart (Kuwahata et al. 2011).

In work published by Masuda and colleagues, 2 weeks of Waon therapy reduced systolic blood pressure in patients who had at least one coronary risk factor (Masuda et al. 2004). This is similar to results reported by Imamura et al. (2001), Kuwahata et al. (2011) and Kihara et al. (2002) reported reductions in systolic blood pressure in patients with coronary risk factors and chronic heart failure following Waon therapy. Imamura and colleagues also reported that their patients had reductions in diastolic blood pressure by ~5 mmHg (Imamura et al. 2001).

Additionally, several studies have investigated whether flow‐mediated dilation (i.e., endothelial function) would improve following Waon therapy in patients with heart disease (Sobajima et al. 2015; Ohori et al. 2012; Kihara et al. 2002). Ohori et al. (2012) reported that following 3 weeks of Waon therapy, patients with chronic heart failure had ~2% improvements in flow‐mediated dilation. In this same investigation, 6‐min walk scores and exercise tolerance also improved (Ohori et al. 2012). Similarly, Kihara et al. (2002) also reported improvements in flow‐mediated dilation following 2 weeks of Waon therapy in patients with chronic heart failure. Interestingly, they noted that their patients' improvements were purely endothelium‐mediated, as there was no change in the endothelial‐independent vasodilation (assessed via nitroglycerin administration) (Kihara et al. 2002). Finally, Sobajima et al. reported that 3 weeks of Waon therapy also improved flow‐mediated dilation in patients with chronic heart failure (Sobajima et al. 2015). Taken together, these data indicate that in patients with chronic heart failure, Waon therapy improves endothelial‐dependent dilation via flow‐mediated dilation.

Although these improvements in vascular function and blood pressure are impressive, it is important to note a few experimental considerations. First, the starting blood pressures for all the groups mentioned would be classified as prehypertensive or normal blood pressure according to AHA/ACC guidelines (Flack and Adekola 2020; Whelton et al. 2017). Thus, these reductions in blood pressure are in line with the reductions in healthy older adults reported by Ruiz‐Pick et al. 2025 and may not translate to patient populations who have higher blood pressure or more aggressive forms of heart disease in other parts of the world (i.e., the United States).

Second, the improvements in health following Waon therapy have occurred over a relatively short period, that is, 5 days to 2 weeks (Kihara et al. 2009; Kuwahata et al. 2011; Miyata et al. 2008; Shinsato et al. 2010; Tei et al. 2007; Fujita et al. 2011; Ohori et al. 2012). Therefore, it is unknown how long these adaptations last or whether the same durations and frequencies used in these studies will translate to a worldwide population with these same risk factors/diseases. It is important that others across the world conduct more in‐depth trials with greater ecological validity to determine if the beneficial adaptations of Waon therapy will apply to other patients.

In contrast to the Waon therapy trials, Debray et al. (2023) did not see any improvements following heat therapy in their patients with coronary artery disease. They utilized Finnish sauna as their heating modality and asked participants to complete 8 weeks of heat therapy. They had hypothesized that following 8 weeks of sauna bathing, their patients with coronary artery disease would have improvements in blood pressure and macro‐ and microvascular function. Contrary to their hypothesis, they reported no change in blood pressure, arterial stiffness via carotid‐femoral pulse wave velocity, endothelial function via flow‐mediated dilation, or cutaneous microvascular responses to local heating (Debray et al. 2023). Interestingly, they did report reductions in reactive hyperemia in their sauna group and improvements in reactive hyperemia in their control group. However, as the authors state, these are small changes that may not be physiologically significant (Debray et al. 2023).

In summary, these data could indicate one of two things: (1) that patients with coronary artery disease need a stronger stimulus (i.e., increased frequency or duration) to improve arterial stiffness, or (2) that heat therapy via Finnish sauna is not enough to reverse the decrements in vascular function. Thus, we again see the equivocal nature of heat therapy. Therefore, we maintain that determining the best modality for heat therapy as well as the best frequency, temperature, and duration (FTDM) to drive changes in patients with heart disease should be a priority among researchers looking for alternative treatment options.

#### 7.3.1 Impact of Heat Therapy on Cardiac Function and Health in Adults With Heart Disease

Acute effects of heat exposure and their cardiac adjustments have been well‐documented in healthy adults (Brothers et al. 2009; Gagnon et al. 2016, 2017; Crandall and Wilson 2015; Wilson and Crandall 2011; Gayda, Paillard, et al. 2012). Interestingly, less investigated are cardiac changes in response to heat therapy. Those studies that have investigated these effects have used Waon therapy to assess improvements in patients with coronary heart disease and chronic heart failure. They have reported beneficial adaptations following heat therapy in these groups. Kuwahata and colleagues report that following 4 weeks of Waon therapy, patients with chronic heart failure had improvements in left ventricular ejection fraction, cardiac output, and left ventricular end diastolic volume (Kuwahata et al. 2011). These patients also had reductions in brain natriuretic peptide (BNP; a blood‐based biomarker of heart disease), noradrenaline, and right ventricular–Tei index (Kuwahata et al. 2011). Kihara and colleagues demonstrated similar findings following 2 weeks of Waon therapy in patients with chronic heart failure following 2 weeks of Waon therapy (Kuwahata et al. 2011). These patients improved their NYHA functional classification (6 people were reclassified to a lower classification), reduced their cardiothoracic ratio, and substantially reduced plasma BNP levels (Kihara et al. 2002). The same improvements occurred in patients studied by Miyata and colleagues, with additional improvements in ejection fraction, left ventricular diastolic dimension, and left atrial dimension (Miyata et al. 2008). Others, such as Ohori and colleagues, reported reclassification to a lower NYHA class in 7 of their patients and reductions in plasma BNP and norepinephrine, but no improvements in cardiac function as measured via ejection fraction or ventricular dimensions (Ohori et al. 2012). The reason for this discrepancy could be due to the differences in length of the interventions or participant variability.

These studies and others have made it clear that Waon therapy improves cardiac function and cardiac health in patients with chronic heart failure (Oyama et al. 2013; Sobajima et al. 2015; Ye et al. 2020). The next step for this research should be to determine the mechanisms driving these changes in cardiac function and if other heat therapy modalities offer the same beneficial adaptations as Waon therapy.

As can be appreciated in this section, the impact of heat therapy on cardiac function is equivocal. Some groups and conditions report a greater magnitude of change that appears to be modality and dose‐dependent. A greater consensus as to the appropriate FTDM principle should be the priority of researchers interested in improving health among those with cardiac disease. It would be beneficial to investigate first whether certain conditions and world populations respond differently to differing modalities. Heat therapy should not be considered a one‐time fix for conditions with decreased cardiovascular function. Rather, as Laukkanen and colleagues have reported (Laukkanen and Kunutsor 2024; Laukkanen et al. 2015), it should be done consistently throughout your life. Additionally, given the different modalities that have been used across various studies, meta‐analyses such as Ye et al. (2020) may not accurately describe the impact of heat therapy in various conditions such as heart failure. As such, further work is needed to determine the appropriate “prescription” for this population.

### 7.4 Polycystic Ovary Syndrome

Polycystic ovary syndrome is a gynecological and endocrine disorder that is accompanied by elevated cardiovascular disease risk and autonomic dysfunction. This increased risk often includes hypertension, glucose resistance, and decreased insulin sensitivity (Ely, Clayton, McCurdy, et al. 2019; Ely, Francisco, Halliwill, et al. 2019; Wild et al. 2010; Lansdown and Rees 2012; Di Domenico et al. 2013). Although there are pharmacological treatments to address hormone dysregulation and metabolic function, as of yet, there are no treatments that directly address the increased cardiovascular disease risk. Ely, Clayton, McCurdy, et al. (2019) investigated the effect of 8–10 weeks of hot water immersion (60 min per session, 3–4 times per week) in 40°C water on cardiovascular health in women with PCOS.

**FIGURE 14:**
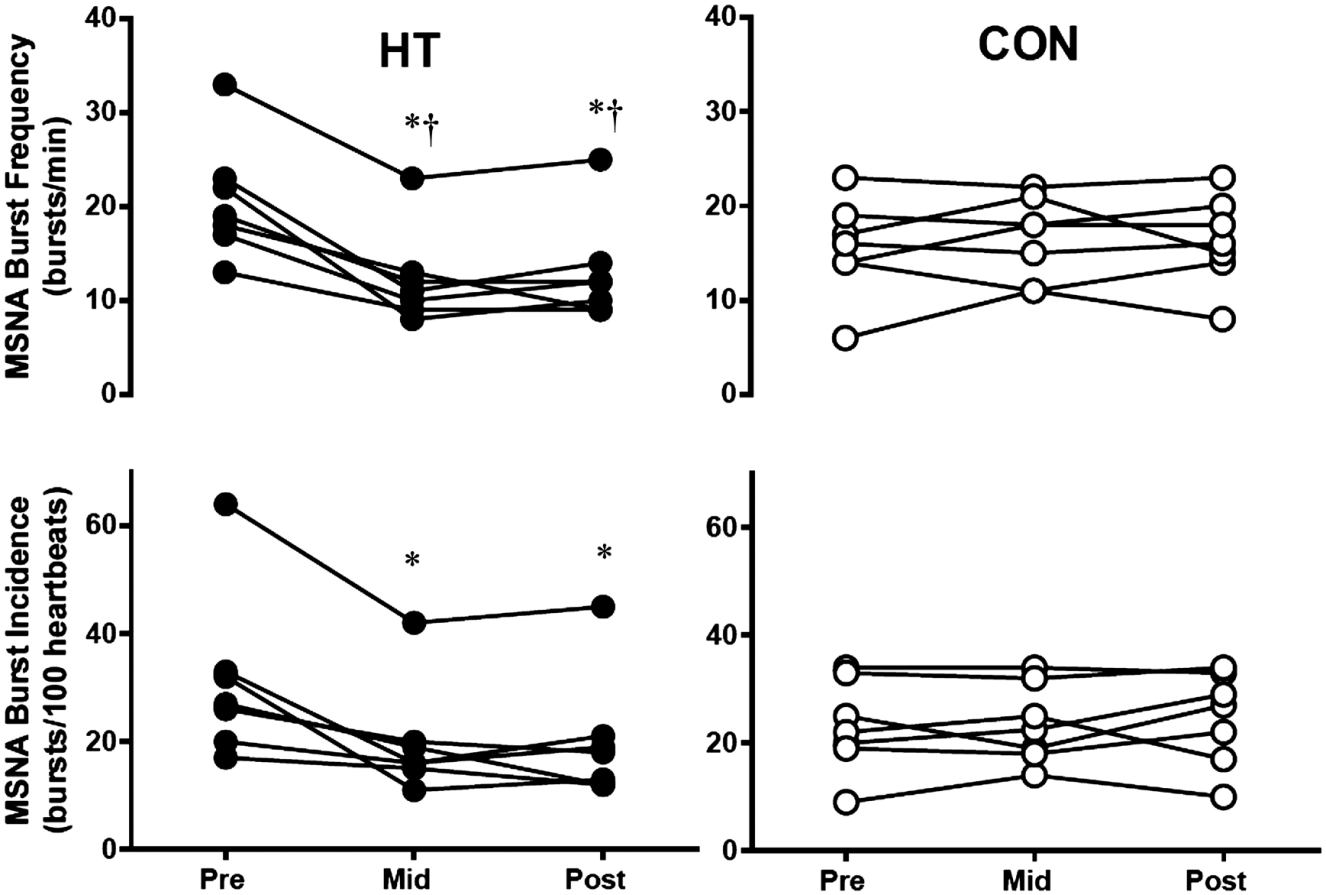
Ely et al. reported muscle sympathetic nerve activity (MSNA), measured in the peroneal nerve as burst frequency and burst incidence in heat therapy (HT) and control (CON) participants with PCOS. MSNA was measured before (Pre), during (Mid), and after (Post) the interventions. *p < 0.05 difference from Pre within group. ^†^ *p* < 0.05 from CON at matched time point. Figure used with permission. Previously published in the *American Journal of Physiology‐Regulatory, Integrative, and Comparative Physiology* (Figure 2; Ely, Clayton, McCurdy, et al. 2019).

Women in the heat therapy group had reductions in systolic, diastolic, and mean arterial blood pressure following heat therapy. Impressively, the reductions were as much as 9–10 mmHg from their starting pressures, which were prehypertensive (systolic pressure of ~124 mmHg Ely, Clayton, McCurdy, et al. 2019). They also reported that following 4 weeks of chronic hot water immersion, women in the heat therapy group had a ~40% reduction in MSNA burst incidence compared to preintervention values (see Figure 14). This reduction is comparable to 16 weeks of moderate intensity exercise training or electroacupuncture in the same population (Stener‐Victorin et al. 2009). Thus, it is likely that this substantial reduction in burst incidence, which was maintained after the completion of heat therapy, plays a critical role in the reduction of systolic and diastolic blood pressure (Ely, Clayton, McCurdy, et al. 2019). Interestingly, the serum HSP72 response did not follow this same pattern (Ely, Francisco, et al. 2019). Given that previous work (Johnson et al. 2005; Whitham et al. 2006) has reported a positive association between sympathetic activity and greater alpha‐adrenergic activation with an increase in HSP72, future work should focus on the mechanisms underlying the elevated sympathetic activity in PCOS that are reduced with heat therapy, and the potential relationships between HSP72 and alpha‐adrenergic responses in the human vasculature.

These participants also had reductions in large artery wall thickness (femoral and carotid arteries), brachial‐ankle pulse wave velocity (a measure of arterial stiffness), and endothelial function measured via flow‐mediated dilation following ischemia–reperfusion injury (Ely, Clayton, McCurdy, et al. 2019). Finally, Ely, Francisco, et al. (2019) reported that fasting glucose was also reduced following the heat therapy intervention.

These data are compelling and suggest that heat therapy can improve various indices of cardiovascular and metabolic health in women with PCOS. Future work should determine whether the improvements in vascular function were accompanied by improvements in structure through vascular remodeling, as this was not directly measured in this study. These data suggest that whole‐body heat exposure for 60 min per session for 8–10 weeks is an appropriate FTDM to trigger beneficial adaptations. Furthermore, they provide evidence for the potential of this heat therapy modality to improve health in other populations with similar conditions.

### 7.5 Peripheral Artery Disease

Peripheral artery disease is characterized by chronic limb ischemia, decrements in functional and exercise capacity, and intermittent claudication due to atherosclerotic plaque in the arteries of the legs. Finding helpful therapeutic treatments to improve the quality of life in patients with peripheral artery disease is paramount (Shamaki et al. 2022). Interestingly, the beneficial improvements due to heat therapy in this population have differed based on heating modality and the FTDM principle. For instance, in this group, it seems as though heat therapy alone does not improve blood pressure or calf microvascular function, regardless of the duration of heat therapy (Monroe et al. 2020, 2022). In work conducted by Monroe et al., they tested the hypothesis that various cardiovascular indices and functional capacity would be improved following 6 weeks (Monroe et al. 2020) and 8 weeks (Monroe et al. 2022) of lower leg heat therapy, using water‐perfused pants. They reported that in both the 6 and 8 week interventions, there was no change in blood pressure or calf reactive hyperemia despite two different assigned heating frequencies (see Table 2). In contrast to the lack of improvement reported by Monroe et al., Akerman and colleagues (Akerman et al. 2019) demonstrated that whole‐body water immersion heat therapy in combination with calisthenics (30 min of heat therapy immediately followed by 30 min of calisthenics) substantially reduced blood pressure and improved pain‐free walking distance in patients with peripheral artery disease.

**FIGURE 15:**
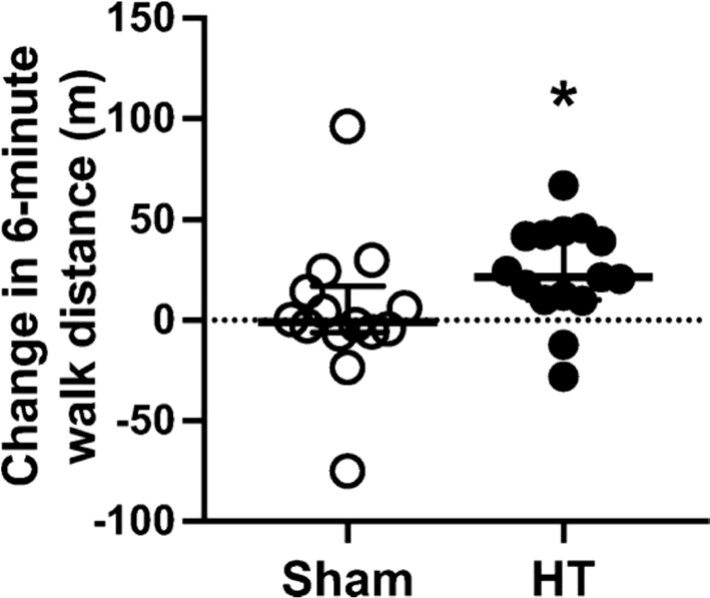
Monroe et al. reported the change in 6‐min walk distance pre–post heat therapy for a sham (*n* = 14; open circles) and heat therapy (HT, *n* = 15; closed circles) condition. Median and interquartile range of changes in 6‐min walk distance from baseline to the 8‐week follow‐up. *p < 0.05 Sham vs. HT. Figure used with permission. Previously published in the *Journal of Applied Physiology* (Figure 3; Monroe et al. 2022).

Waon therapy has also been shown to elicit beneficial improvements in this population. Tei et al. (2007) reported that following 10 weeks of Waon therapy, their patients with peripheral artery disease had improved functional capacity and ankle‐brachial pressure index, a measure of arterial stiffness (Tei et al. 2007). Interestingly, Monroe et al. reported that following the 8 weeks of heat therapy, participants improved their 6‐min walk distance but had no change in ankle‐brachial index (Figure 15; Monroe et al. 2022). Taken together, these data suggest that not all heat therapy has the same beneficial impact on patients with the same condition. Therefore, more research is needed to elucidate the best heating modality and FTDM principle for improving vascular stiffness and functional capacity in patients with peripheral artery disease. This is in agreement with Harwood et al. (2021). In their meta‐analysis, it appears that more studies than not show improvements in walking distance, but the data regarding improvements in arterial stiffness are varied between studies. One potential contributor to the improvement in walking distance could be vascular remodeling. If heat therapy is influencing changes in the vessel's structure, this would directly impact blood flow during walking and contribute to the associated fatigue. Furthermore, future studies should target whether structural adaptations occur with heat therapy in patients with peripheral artery disease to determine whether assessments that have been used previously have failed to capture these changes.

## 8 Limitations and Considerations

Heat therapy encompasses many different modalities and dosages. Due to the degree of variation and the different ways of implementation, the results from heat therapy research are equivocal. By way of example, there is substantial evidence that regular traditional or Finnish sauna use is associated with improved health outcomes and morbidity. In contrast, there is little strength of evidence at this time that regular FIR sauna use improves health outcomes that match the commercial hype, with the exception of early exciting work on improving depression. The strength of evidence for realized health gains with heat therapy is strongest when there is an increase in temperature (e.g., core, organ, skin). Unfortunately, not all studies report this variable. For example, Waon therapy is one modality that has repeatedly been shown to improve cardiovascular health in clinical populations, but body core temperature is rarely, if ever, reported. Thus, understanding the mechanisms that underlie the improvements in cardiovascular health in these studies can be difficult, as is ensuring the practice is achieving the targets aligning with the original studies showing benefit. Those interested in heat therapy should pay attention to the temperatures reported to best understand the impact of the intervention.

Participant population should also be critically evaluated with heat therapy research. As we have discussed, some clinical groups have improvements in various aspects of health (e.g., blood pressure, vascular function) while others do not. Geographic and cultural influences, in addition to health status, can play a significant role in whether heat therapy can improve symptomology. These factors should be critically evaluated when interpreting and generalizing results.

## 9 Future Directions and Conclusion

In many ways, the current state of evidence supporting the use of heat therapy is reminiscent of the early days of “exercise as medicine.” Both heat therapy and physical activity have deep cultural roots linking them to health and wellness, disease prevention, and healing. As such, the serious scientific investigation of the health benefits of each did not occur until long afterward. As with many health interventions, early publications often present more positive outcomes or larger effects than later investigations. While this may be partly due to publication bias, it can also be attributed to greater experimental control, smaller and more selective cohorts of participants, and perhaps self‐selection of participants with higher patient activation in the initial stages of inquiry. Early positive results are often countered by later evidence that is less clear, or by negative results, as investigations shift toward the inclusion of larger, more heterogeneous cohorts and/or protocols that are more ecologically valid but have less stringent control over conditions.

It is these investigative shifts that we hope will inform future heat therapy research. As we have described, heat therapy has been used for hundreds if not thousands of years in various cultures and populations worldwide. We, as scientists, need to shift the focus of our research to deliberately crafting more ecologically valid experimental designs. Although the inclusion of individuals taking medications, with a wide range of body mass indexes, or with comorbidities may muddy the interpretation, it will expand the utility and understanding of heat therapy, thereby making the prescription of heat therapy a legitimate option.

These future directions, though, need to be carefully thought out, as one confound in this process is the multitude of combinations of stressors that can be presented as “exercise” or “heat therapy.” Using drug dosing as an analogy, it is not reasonable to expect that all doses of exercise or all doses of heat therapy will generate the same results. For these many reasons, the pendulum may swing against favoring an intervention, only to gain support again later when hindsight is developed or large‐scale clinical trials with appropriate and robust controls are reported. At one time, it was not clear that exercise was an effective means to manage hypertension. Yet, now, it would be considered unethical not to recommend that a patient with high blood pressure participate in regular exercise as a critical lifestyle intervention. It is too early to declare that heat therapy is an equally efficacious lifestyle intervention, as we may still be in the first wave of positive outcomes. It may take some time before we reach a final consensus on its role in health and disease prevention. However, from a different perspective than the evidence‐based medicine formulation, we can ask how well heat therapy stands up to the Bradford Hill criteria for causation (Hill 1965). These nine criteria were proposed by Sir Austin Bradford to assess the likelihood of a causal relationship between an exposure and an outcome. The nine criteria are: (1) strength of association; (2) consistency; (3) specificity; (4) temporality; (5) dose–response; (6) plausibility; (7) coherence; (8) experiment; and (9) analogy. Across the literature on adaptation to heat and heat therapy, *experiments* show *dose–response* relationships and *temporality* with heat exposure that are *consistent* when observed across labs and datasets and are *strong effects* when looking at *specific* outcome variables or *specific* populations. Furthermore, there is, more often than not, *coherence* between smaller, tightly controlled studies and epidemiological surveys. We have identified *plausible mechanisms* such as shear stress, and heat‐sensitive gene expression and find heat therapy to be *analogous* to exercise in many aspects. That's Hill's criteria, served up hot.

## Author Contributions

R.E.R., R.D.H., B.W.K., P.C.G., J.R.H., and C.T.M. wrote the manuscript. R.E.R., R.D.H., B.W.K., P.C.G., J.R.H., and C.T.M. edited, revised, and approved the final version of the manuscript.

## Funding

Funding for investigators was provided by the National Institutes of Health (HL144128, C.T.M. and J.R.H.; AG081304, AG072973, P.C.G.) and the American Heart Association (19TPA34890033, C.T.M.).

## Conflicts of Interest

The authors declare no conflicts of interest.

## Data Availability

Data sharing not applicable to this article as no datasets were generated or analyzed during the current study.

## References

[cph470089-bib-0001] Abel, A. N. , L. K. Lloyd , J. S. Williams , and B. K. Miller . 2012. “Physiological Characteristics of Long‐Term Bikram Yoga Practitioners.” Journal of Exercise Physiology Online 15: 32–39.

[cph470089-bib-0002] Acquarone, D. , A. Bertero , M. Brancaccio , and M. Sorge . 2024. “Chaperone Proteins: The Rising Players in Muscle Atrophy.” Journal of Cachexia, Sarcopenia and Muscle 16: e13659. 39707668 10.1002/jcsm.13659 PMC11747685

[cph470089-bib-0003] Adlard, P. A. , V. M. Perreau , V. Pop , and C. W. Cotman . 2005. “Voluntary Exercise Decreases Amyloid Load in a Transgenic Model of Alzheimer's Disease.” Journal of Neuroscience 25: 4217–4221. 15858047 10.1523/JNEUROSCI.0496-05.2005 PMC6725122

[cph470089-bib-0004] Ahlskog, J. E. , Y. E. Geda , N. R. Graff‐Radford , and R. C. Petersen . 2011. “Physical Exercise as a Preventive or Disease‐Modifying Treatment of Dementia and Brain Aging.” Mayo Clinic Proceedings 86: 876–884. 21878600 10.4065/mcp.2011.0252 PMC3258000

[cph470089-bib-0005] Akerman, A. P. , K. N. Thomas , A. M. van Rij , E. D. Body , M. Alfadhel , and J. D. Cotter . 2019. “Heat Therapy vs. Supervised Exercise Therapy for Peripheral Arterial Disease: A 12‐Wk Randomized, Controlled Trial.” American Journal of Physiology. Heart and Circulatory Physiology 316: H1495–H1506. 31002283 10.1152/ajpheart.00151.2019

[cph470089-bib-0006] Akhtar, M. W. , S. Sanz‐Blasco , N. Dolatabadi , et al. 2016. “Elevated Glucose and Oligomeric beta‐Amyloid Disrupt Synapses via a Common Pathway of Aberrant Protein S‐Nitrosylation.” Nature Communications 7: 10242. 10.1038/ncomms10242 PMC4729876 26743041

[cph470089-bib-0007] Akins, J. , R. Takeda , T. Washio , et al. 2024. “Neural‐Cardiovascular Responses to Orthostasis Following 8‐Weeks of Home‐Based Lower Leg Heating in Older Women With Hypertension.” Physiology 39: 601.

[cph470089-bib-0008] Akinyemi, R. O. , E. B. Mukaetova‐Ladinska , J. Attems , M. Ihara , and R. N. Kalaria . 2013. “Vascular Risk Factors and Neurodegeneration in Ageing Related Dementias: Alzheimer's Disease and Vascular Dementia.” Current Alzheimer Research 10: 642–653. 23627757 10.2174/15672050113109990037

[cph470089-bib-0009] Alhajj, M. , N. G. Nelson , and L. B. McKenzie . 2009. “Hot Tub, Whirlpool, and Spa‐Related Injuries in the U.S., 1990–2007.” American Journal of Preventive Medicine 37: 531–536. 19944920 10.1016/j.amepre.2009.08.024

[cph470089-bib-0010] Araujo, J. , M. Zhang , and F. Yin . 2012. “Heme Oxygenase‐1, Oxidation, Inflammation, and Atherosclerosis.” Frontiers in Pharmacology 3:119. 22833723 10.3389/fphar.2012.00119 PMC3400084

[cph470089-bib-0011] Archer, A. E. , R. S. Rogers , A. T. Von Schulze , et al. 2018. “Heat Shock Protein 72 Regulates Hepatic Lipid Accumulation.” American Journal of Physiology. Regulatory, Integrative and Comparative Physiology 315: R696–R707. 29924632 10.1152/ajpregu.00073.2018 PMC6230886

[cph470089-bib-0012] Arvanitakis, Z. , R. S. Wilson , J. L. Bienias , D. A. Evans , and D. A. Bennett . 2004. “Diabetes Mellitus and Risk of Alzheimer Disease and Decline in Cognitive Function.” Archives of Neurology 61: 661–666. 15148141 10.1001/archneur.61.5.661

[cph470089-bib-0013] Ashby, E. L. , J. S. Miners , P. G. Kehoe , and S. Love . 2016. “Effects of Hypertension and Anti‐Hypertensive Treatment on Amyloid‐beta (Abeta) Plaque Load and Abeta‐Synthesizing and Abeta‐Degrading Enzymes in Frontal Cortex.” Journal of Alzheimer's Disease 50: 1191–1203. 10.3233/JAD-150831 26836178

[cph470089-bib-0447] Atencio, J. K. , E. L. Reed , K. Wiedenfeld Needham , et al. 2025. “Comparison of Thermoregulatory, Cardiovascular, and Immune Responses to Different Passive Heat Therapy Modalities.” American Journal of Physiology. Regulatory, Integrative and Comparative Physiology 329, no. 1: R20–R35. 10.1152/ajpregu.00012.2025. 40332494 PMC13178818

[cph470089-bib-0015] Auerbach, R. P. , P. Mortier , R. Bruffaerts , et al. 2018. “WHO World Mental Health Surveys International College Student Project: Prevalence and Distribution of Mental Disorders.” Journal of Abnormal Psychology 127: 623–638. 30211576 10.1037/abn0000362 PMC6193834

[cph470089-bib-0016] Averna, M. , R. Stifanese , R. De Tullio , et al. 2008. “Functional Role of HSP90 Complexes With Endothelial Nitric‐Oxide Synthase (eNOS) and Calpain on Nitric Oxide Generation in Endothelial Cells.” Journal of Biological Chemistry 283: 29069–29076. 18682401 10.1074/jbc.M803638200 PMC2662009

[cph470089-bib-0441] Bailey, T. , N. Cable , G. Miller , V. Sprung , D. Low , and H. Jones . 2016. “Repeated Warm Water Immersion Induces Similar Cerebrovascular Adaptations to 8 Weeks of Moderate‐Intensity Exercise Training in Females.” International Journal of Sports Medicine 37, no. 10: 757–765. 10.1055/s-0042-106899. 27286178

[cph470089-bib-0017] Baranwal, N. , P. K. Yu , and N. S. Siegel . 2023. “Sleep Physiology, Pathophysiology, and Sleep Hygiene.” Progress in Cardiovascular Diseases 77: 59–69. 36841492 10.1016/j.pcad.2023.02.005

[cph470089-bib-0018] Bastard, J. P. , M. Maachi , C. Lagathu , et al. 2006. “Recent Advances in the Relationship Between Obesity, Inflammation, and Insulin Resistance.” European Cytokine Network 17: 4–12. 16613757

[cph470089-bib-0019] Bauer, J. , F. Hohagen , E. Gimmel , et al. 1995. “Induction of Cytokine Synthesis and Fever Suppresses REM Sleep and Improves Mood in Patients With Major Depression.” Biological Psychiatry 38: 611–621. 8573663 10.1016/0006-3223(95)00374-x

[cph470089-bib-0020] Beever, R. 2009. “Far‐Infrared Saunas for Treatment of Cardiovascular Risk Factors: Summary of Published Evidence.” Canadian Family Physician 55: 691–696. 19602651 PMC2718593

[cph470089-bib-0021] Behzadi, P. , N. Ravanelli , H. Gravel , et al. 2022. “Acute Effect of Passive Heat Exposure on Markers of Cardiometabolic Function in Adults With Type 2 Diabetes Mellitus.” Journal of Applied Physiology 132: 1154–1166. 35323077 10.1152/japplphysiol.00800.2021

[cph470089-bib-0022] Belhadj Slimen, I. , T. Najar , A. Ghram , H. Dabbebi , M. Ben Mrad , and M. Abdrabbah . 2014. “Reactive Oxygen Species, Heat Stress and Oxidative‐Induced Mitochondrial Damage. A Review.” International Journal of Hyperthermia 30: 513–523. 25354680 10.3109/02656736.2014.971446

[cph470089-bib-0023] Bellien, J. , J. Favre , M. Iacob , et al. 2010. “Arterial Stiffness Is Regulated by Nitric Oxide and Endothelium‐Derived Hyperpolarizing Factor During Changes in Blood Flow in Humans.” Hypertension 55: 674–680. 20083732 10.1161/HYPERTENSIONAHA.109.142190

[cph470089-bib-0024] Billecke, S. S. , A. T. Bender , K. C. Kanelakis , et al. 2002. “hsp90 Is Required for Heme Binding and Activation of Apo‐Neuronal Nitric‐Oxide Synthase: Geldanamycin‐Mediated Oxidant Generation Is Unrelated to Any Action of hsp90.” Journal of Biological Chemistry 277: 20504–20509. 11923316 10.1074/jbc.M201940200

[cph470089-bib-0025] Billman, G. E. , H. V. Huikuri , J. Sacha , and K. Trimmel . 2015. “An Introduction to Heart Rate Variability: Methodological Considerations and Clinical Applications.” Frontiers in Physiology 6: 55. 25762937 10.3389/fphys.2015.00055 PMC4340167

[cph470089-bib-0026] Biro, S. , A. Masuda , T. Kihara , and C. Tei . 2003. “Clinical Implications of Thermal Therapy in Lifestyle‐Related Diseases.” Experimental Biology and Medicine 228: 1245–1249. 14610268 10.1177/153537020322801023

[cph470089-bib-0027] Black, M. A. , N. T. Cable , D. H. Thijssen , and D. J. Green . 2009. “Impact of Age, Sex, and Exercise on Brachial Artery Flow‐Mediated Dilatation.” American Journal of Physiology. Heart and Circulatory Physiology 297: H1109–H1116. 19633208 10.1152/ajpheart.00226.2009 PMC2755978

[cph470089-bib-0028] Blankenship, A. E. , R. Kemna , P. J. Kueck , et al. 2025. “Improving Glycemic Control via Heat Therapy in Older Adults at Risk for Alzheimer's Disease (FIGHT‐AD): A Pilot Study.” Journal of Applied Physiology (Bethesda, MD: 1985) 138: 720–730. 39829076 10.1152/japplphysiol.00396.2024 PMC12439486

[cph470089-bib-0029] Bobkova, N. V. , D. G. Garbuz , I. Nesterova , et al. 2014. “Therapeutic Effect of Exogenous hsp70 in Mouse Models of Alzheimer's Disease.” Journal of Alzheimer's Disease 38: 425–435. 10.3233/JAD-130779 23985416

[cph470089-bib-0030] Boutouyrie, P. , P. Chowienczyk , J. D. Humphrey , and G. F. Mitchell . 2021. “Arterial Stiffness and Cardiovascular Risk in Hypertension.” Circulation Research 128: 864–886. 33793325 10.1161/CIRCRESAHA.121.318061

[cph470089-bib-0031] Brengelmann, G. L. , M. McKeag , and L. B. Rowell . 1977. “Temperature Control System for Water‐Perfused Suits.” Journal of Applied Physiology 42: 656–660. 863829 10.1152/jappl.1977.42.4.656

[cph470089-bib-0032] Brito, L. C. , T. C. Marin , L. Azevêdo , J. M. Rosa‐Silva , S. A. Shea , and S. S. Thosar . 2022. “Chronobiology of Exercise: Evaluating the Best Time to Exercise for Greater Cardiovascular and Metabolic Benefits.” Compr Physiol 12: 3621–3639. 35766829 10.1002/cphy.c210036 PMC10214902

[cph470089-bib-0033] Brothers, R. M. , P. S. Bhella , S. Shibata , J. E. Wingo , B. D. Levine , and C. G. Crandall . 2009. “Cardiac Systolic and Diastolic Function During Whole Body Heat Stress.” American Journal of Physiology. Heart and Circulatory Physiology 296: H1150–H1156. 19218504 10.1152/ajpheart.01069.2008 PMC2670696

[cph470089-bib-0034] Brouet, A. , P. Sonveaux , C. Dessy , J.‐L. Balligand , and O. Feron . 2001. “Hsp90 Ensures the Transition From the Early Ca2+−Dependent to the Late Phosphorylation‐Dependent Activation of the Endothelial Nitric‐Oxide Synthase in Vascular Endothelial Growth Factor‐Exposed Endothelial Cells.” Journal of Biological Chemistry 276: 32663–32669. 11425855 10.1074/jbc.M101371200

[cph470089-bib-0035] Bruce, C. R. , A. L. Carey , J. A. Hawley , and M. A. Febbraio . 2003. “Intramuscular Heat Shock Protein 72 and Heme Oxygenase‐1 mRNA Are Reduced in Patients With Type 2 Diabetes: Evidence That Insulin Resistance Is Associated With a Disturbed Antioxidant Defense Mechanism.” Diabetes 52: 2338–2345. 12941774 10.2337/diabetes.52.9.2338

[cph470089-bib-0036] Brunt, V. E. , T. M. Eymann , M. A. Francisco , M. J. Howard , and C. T. Minson . 2016. “Passive Heat Therapy Improves Cutaneous Microvascular Function in Sedentary Humans via Improved Nitric Oxide‐Dependent Dilation.” Journal of Applied Physiology 121: 716–723. 27418688 10.1152/japplphysiol.00424.2016 PMC6195670

[cph470089-bib-0037] Brunt, V. E. , N. Fujii , and C. T. Minson . 2015. “Endothelial‐Derived Hyperpolarization Contributes to Acetylcholine‐Mediated Vasodilation in Human Skin in a Dose‐Dependent Manner.” Journal of Applied Physiology 119: 1015–1022. 26384409 10.1152/japplphysiol.00201.2015 PMC4628993

[cph470089-bib-0038] Brunt, V. E. , M. J. Howard , M. A. Francisco , B. R. Ely , and C. T. Minson . 2016. “Passive Heat Therapy Improves Endothelial Function, Arterial Stiffness and Blood Pressure in Sedentary Humans.” Journal of Physiology 594: 5329–5342. 27270841 10.1113/JP272453 PMC5023696

[cph470089-bib-0039] Brunt, V. E. , and C. T. Minson . 2021. “Heat Therapy: Mechanistic Underpinnings and Applications to Cardiovascular Health.” Journal of Applied Physiology 130: 1684–1704. 33792402 10.1152/japplphysiol.00141.2020 PMC8285605

[cph470089-bib-0040] Brunt, V. E. , H. L. Rosenberg , A. E. Bazzoni , et al. 2019. “Passive Heat Therapy Lowers Systolic Blood Pressure and Improves Vascular Endothelial Function in Healthy Older Adults.” FASEB Journal 33: 829.822.

[cph470089-bib-0041] Brunt, V. E. , K. M. Weidenfeld‐Needham , L. N. Comrada , M. A. Francisco , T. M. Eymann , and C. T. Minson . 2019. “Serum From Young, Sedentary Adults Who Underwent Passive Heat Therapy Improves Endothelial Cell Angiogenesis via Improved Nitric Oxide Bioavailability.” Temperature 6: 169–178. 10.1080/23328940.2019.1614851 PMC6601412 31286027

[cph470089-bib-0042] Brunt, V. E. , K. Wiedenfeld‐Needham , L. N. Comrada , and C. T. Minson . 2018. “Passive Heat Therapy Protects Against Endothelial Cell Hypoxia‐Reoxygenation via Effects of Elevations in Temperature and Circulating Factors.” Journal of Physiology 596: 4831–4845. 30118148 10.1113/JP276559 PMC6187037

[cph470089-bib-0043] Buguet, A. 2007. “Sleep Under Extreme Environments: Effects of Heat and Cold Exposure, Altitude, Hyperbaric Pressure and Microgravity in Space.” Journal of the Neurological Sciences 262: 145–152. 17706676 10.1016/j.jns.2007.06.040

[cph470089-bib-0044] Calderwood, S. K. , A. Murshid , and T. Prince . 2009. “The Shock of Aging: Molecular Chaperones and the Heat Shock Response in Longevity and Aging—A Mini‐Review.” Gerontology 55: 550–558. 19546513 10.1159/000225957 PMC2754743

[cph470089-bib-0045] Caldwell, A. R. , F. B. Robinson , M. A. Tucker , et al. 2017. “Effect of Passive Heat Stress and Exercise in the Heat on Arterial Stiffness.” European Journal of Applied Physiology 117: 1679–1687. 28612122 10.1007/s00421-017-3658-1

[cph470089-bib-0046] Carey, A. L. , G. R. Steinberg , S. L. Macaulay , et al. 2006. “Interleukin‐6 Increases Insulin‐Stimulated Glucose Disposal in Humans and Glucose Uptake and Fatty Acid Oxidation in Vitro via AMP‐Activated Protein Kinase.” Diabetes 55: 2688–2697. 17003332 10.2337/db05-1404

[cph470089-bib-0047] Carman, A. , S. Kishinevsky , J. Koren III , W. Lou , and G. Chiosis . 2013. “Chaperone‐Dependent Neurodegeneration: A Molecular Perspective on Therapeutic Intervention.” Journal of Alzheimers Disease & Parkinsonism: 2013. (Suppl 10):007. 10.4172/2161-0460.S10-007 PMC4172285 25258700

[cph470089-bib-0048] Carter, H. H. , A. L. Spence , C. L. Atkinson , et al. 2014. “Distinct Effects of Blood Flow and Temperature on Cutaneous Microvascular Adaptation.” Medicine & Science in Sports & Exercise 46: 2113–2121. 25338190 10.1249/MSS.0000000000000349

[cph470089-bib-0049] Chadwick, A. L. , C. Shi , M. McMillan , J. Miller , J. Hu , and P. C. Geiger . 2025. “The Impact of a Heat Therapy Intervention on Pain and Fibromyalgia Symptoms in Patients With Fibromyalgia: A Pilot Study.” Frontiers in Pain Research (Lausanne) 6: 1526491. 10.3389/fpain.2025.1526491 PMC11966051 40182803

[cph470089-bib-0050] Chambers, C. D. 2006. “Risks of Hyperthermia Associated With Hot Tub or spa Use by Pregnant Women.” Birth Defects Research Part A: Clinical and Molecular Teratology 76: 569–573. 16998815 10.1002/bdra.20303

[cph470089-bib-0051] Chen, H. W. , S. C. Chen , J. L. Tsai , and R. C. Yang . 1999. “Previous Hyperthermic Treatment Increases Mitochondria Oxidative Enzyme Activity and Exercise Capacity in Rats.” Kaohsiung Journal of Medical Sciences 15: 572–580. 10603704

[cph470089-bib-0052] Cheng, D. , J. Noble , M. X. Tang , N. Schupf , R. Mayeux , and J. A. Luchsinger . 2011. “Type 2 Diabetes and Late‐Onset Alzheimer's Disease.” Dementia and Geriatric Cognitive Disorders 31: 424–430. 21757907 10.1159/000324134 PMC3142096

[cph470089-bib-0053] Cheng, J. L. , and M. J. MacDonald . 2019. “Effect of Heat Stress on Vascular Outcomes in Humans.” Journal of Applied Physiology (1985) 126: 771–781. 10.1152/japplphysiol.00682.2018 PMC6459390 30676869

[cph470089-bib-0054] Cheng, J. L. , C. A. Pizzola , K. C. Mattook , et al. 2024. “Effects of Lower Limb Heat Therapy, Exercise Training, or a Combined Intervention on Vascular Function: A Randomized Controlled Trial.” Medicine and Science in Sports and Exercise 57:94–105. 39283227 10.1249/MSS.0000000000003550

[cph470089-bib-0055] Cheng, J. L. , J. S. Williams , S. P. Hoekstra , and M. J. MacDonald . 2021. “Improvements in Vascular Function in Response to Acute Lower Limb Heating in Young Healthy Males and Females.” Journal of Applied Physiology (1985) 131: 277–289. 10.1152/japplphysiol.00630.2020 34013754

[cph470089-bib-0056] Chirinos, J. A. , P. Segers , T. Hughes , and R. Townsend . 2019. “Large‐Artery Stiffness in Health and Disease: JACC State‐of‐the‐Art Review.” Journal of the American College of Cardiology 74: 1237–1263. 31466622 10.1016/j.jacc.2019.07.012 PMC6719727

[cph470089-bib-0057] Chobanian, A. V. , G. L. Bakris , H. R. Black , et al. 2003. “Seventh Report of the Joint National Committee on Prevention, Detection, Evaluation, and Treatment of High Blood Pressure.” Hypertension 42: 1206–1252. 14656957 10.1161/01.HYP.0000107251.49515.c2

[cph470089-bib-0058] Choi, P. J. , V. E. Brunt , N. Fujii , and C. T. Minson . 2014. “New Approach to Measure Cutaneous Microvascular Function: An Improved Test of NO‐Mediated Vasodilation by Thermal Hyperemia.” Journal of Applied Physiology 117: 277–283. 24903917 10.1152/japplphysiol.01397.2013 PMC4122693

[cph470089-bib-0059] Choudhury, B. 2007. “Bikram Yoga.”

[cph470089-bib-0060] Chung, J. , A. K. Nguyen , D. C. Henstridge , et al. 2008. “HSP72 Protects Against Obesity‐Induced Insulin Resistance.” Proceedings of the National Academy of Sciences of the United States of America 105: 1739–1744. 18223156 10.1073/pnas.0705799105 PMC2234214

[cph470089-bib-0061] Ciechanover, A. , and Y. T. Kwon . 2017. “Protein Quality Control by Molecular Chaperones in Neurodegeneration.” Frontiers in Neuroscience 11: 185. 28428740 10.3389/fnins.2017.00185 PMC5382173

[cph470089-bib-0062] Clayton, Z. S. , D. H. Craighead , S. Darvish , et al. 2022. “Promoting Healthy Cardiovascular Aging: Emerging Topics.” Journal of Cardiovascular Aging 2: 43. 36337728 10.20517/jca.2022.27 PMC9632540

[cph470089-bib-0063] Clijsen, R. , R. Stoop , E. Hohenauer , et al. 2022. “Local Heat Applications as a Treatment of Physical and Functional Parameters in Acute and Chronic Musculoskeletal Disorders or Pain.” Archives of Physical Medicine and Rehabilitation 103: 505–522. 34283996 10.1016/j.apmr.2021.06.015

[cph470089-bib-0064] Coombs, G. B. , O. F. Barak , A. A. Phillips , et al. 2019. “Acute Heat Stress Reduces Biomarkers of Endothelial Activation but Not Macro‐ or Microvascular Dysfunction in Cervical Spinal Cord Injury.” American Journal of Physiology. Heart and Circulatory Physiology 316: H722–H733. 30575438 10.1152/ajpheart.00693.2018 PMC6459313

[cph470089-bib-0065] Crandall, C. G. 2008. “Heat Stress and Baroreflex Regulation of Blood Pressure.” Medicine and Science in Sports and Exercise 40: 2063–2070. 18981943 10.1249/MSS.0b013e318180bc98 PMC2819365

[cph470089-bib-0066] Crandall, C. G. , J. Cui , and T. E. Wilson . 2003. “Effects of Heat Stress on Baroreflex Function in Humans.” Acta Physiologica Scandinavica 177: 321–328. 12609002 10.1046/j.1365-201X.2003.01076.x

[cph470089-bib-0067] Crandall, C. G. , and T. E. Wilson . 2015. “Human Cardiovascular Responses to Passive Heat Stress.” Compr Physiol 5: 17–43. 25589263 10.1002/cphy.c140015 PMC4950975

[cph470089-bib-0068] Cui, J. , Z. Gao , U. A. Leuenberger , et al. 2022. “Repeated Warm Water Baths Decrease Sympathetic Activity in Humans.” Journal of Applied Physiology 133: 234–245. 35736952 10.1152/japplphysiol.00684.2021 PMC9291418

[cph470089-bib-0069] Cui, J. , T. E. Wilson , and C. G. Crandall . 2002a. “Baroreflex Modulation of Sympathetic Nerve Activity to Muscle in Heat‐Stressed Humans.” American Journal of Physiology. Regulatory, Integrative and Comparative Physiology 282: R252–R258. 11742845 10.1152/ajpregu.00337.2001

[cph470089-bib-0070] Cui, J. , T. E. Wilson , and C. G. Crandall . 2002b. “Phenylephrine‐Induced Elevations in Arterial Blood Pressure Are Attenuated in Heat‐Stressed Humans.” American Journal of Physiology. Regulatory, Integrative and Comparative Physiology 283: R1221–R1226. 12376416 10.1152/ajpregu.00195.2002

[cph470089-bib-0071] Currie, R. W. , M. Karmazyn , M. Kloc , and K. Mailer . 1988. “Heat‐Shock Response Is Associated With Enhanced Postischemic Ventricular Recovery.” Circulation Research 63: 543–549. 3409486 10.1161/01.res.63.3.543

[cph470089-bib-0072] Currie, R. W. , and R. M. Tanguay . 1991. “Analysis of RNA for Transcripts for Catalase and SP71 in Rat Hearts After in Vivo Hyperthermia.” Biochemistry and Cell Biology 69: 375–382. 1910735 10.1139/o91-057

[cph470089-bib-0073] Dandona, P. , A. Aljada , and A. Bandyopadhyay . 2004. “Inflammation: The Link Between Insulin Resistance, Obesity and Diabetes.” Trends in Immunology 25: 4–7. 14698276 10.1016/j.it.2003.10.013

[cph470089-bib-0074] de la Monte, S. M. 2012. “Therapeutic Targets of Brain Insulin Resistance in Sporadic Alzheimer's Disease.” Frontiers in Bioscience (Elite Edition) 4: 1582–1605. 22201977 10.2741/482 PMC4550311

[cph470089-bib-0075] de la Torre, J. C. 2004. “Is Alzheimer's Disease a Neurodegenerative or a Vascular Disorder? Data, Dogma, and Dialectics.” Lancet Neurology 3: 184–190. 14980533 10.1016/S1474-4422(04)00683-0

[cph470089-bib-0076] de Matos, M. A. , V. O. Ottone , T. C. Duarte , et al. 2014. “Exercise Reduces Cellular Stress Related to Skeletal Muscle Insulin Resistance.” Cell Stress & Chaperones 19: 263–270. 23975543 10.1007/s12192-013-0453-8 PMC3933613

[cph470089-bib-0077] Debray, A. , H. Gravel , L. Garceau , et al. 2023. “Finnish Sauna Bathing and Vascular Health of Adults With Coronary Artery Disease: A Randomized Controlled Trial.” Journal of Applied Physiology (Bethesda, MD: 1985) 135: 795–804. 37650138 10.1152/japplphysiol.00322.2023

[cph470089-bib-0078] DeFronzo, R. A. , and D. Tripathy . 2009. “Skeletal Muscle Insulin Resistance Is the Primary Defect in Type 2 Diabetes.” Diabetes Care 32, no. Suppl. 2: S157–S163. 19875544 10.2337/dc09-S302 PMC2811436

[cph470089-bib-0079] Desouza, C. A. , L. F. Shapiro , C. M. Clevenger , et al. 2000. “Regular Aerobic Exercise Prevents and Restores Age‐Related Declines in Endothelium‐Dependent Vasodilation in Healthy Men.” Circulation 102: 1351–1357. 10993851 10.1161/01.cir.102.12.1351

[cph470089-bib-0080] Detry, J. M. , G. L. Brengelmann , L. B. Rowell , and C. Wyss . 1972. “Skin and Muscle Components of Forearm Blood Flow in Directly Heated Resting Man.” Journal of Applied Physiology 32: 506–511. 5026500 10.1152/jappl.1972.32.4.506

[cph470089-bib-0081] Dewani, D. , P. Karwade , and K. S. Mahajan . 2023. “The Invisible Struggle: The Psychosocial Aspects of Polycystic Ovary Syndrome.” Cureus 15: e51321. 38288169 10.7759/cureus.51321 PMC10823298

[cph470089-bib-0082] Di Domenico, K. , D. Wiltgen , F. J. Nickel , J. A. Magalhaes , R. S. Moraes , and P. M. Spritzer . 2013. “Cardiac Autonomic Modulation in Polycystic Ovary Syndrome: Does the Phenotype Matter?” Fertility and Sterility 99: 286–292. 23025880 10.1016/j.fertnstert.2012.08.049

[cph470089-bib-0083] Di Naso, F. C. , R. R. Porto , H. S. Fillmann , et al. 2015. “Obesity Depresses the Anti‐Inflammatory HSP70 Pathway, Contributing to NAFLD Progression.” Obesity 23: 120–129. 25292174 10.1002/oby.20919

[cph470089-bib-0084] Dickinson, H. O. , J. M. Mason , D. J. Nicolson , et al. 2006. “Lifestyle Interventions to Reduce Raised Blood Pressure: A Systematic Review of Randomized Controlled Trials.” Journal of Hypertension 24: 215–233. 16508562 10.1097/01.hjh.0000199800.72563.26

[cph470089-bib-0085] Dong, K. H. , D. H. Kyung , and U. M. Ja . 2010. “Changes of Autonomic Nervous Function After Foot Bathing in Normal Adults.” Journal of the Korean Academy of Rehabilitation Medicine 34: 74–78.

[cph470089-bib-0086] Donnelly, T. J. , R. E. Sievers , F. L. Vissern , W. J. Welch , and C. L. Wolfe . 1992. “Heat Shock Protein Induction in Rat Hearts. A Role for Improved Myocardial Salvage After Ischemia and Reperfusion?” Circulation 85: 769–778. 1735169 10.1161/01.cir.85.2.769

[cph470089-bib-0087] Dorsey, C. M. , S. E. Lukas , M. H. Teicher , et al. 1996. “Effects of Passive Body Heating on the Sleep of Older Female Insomniacs.” Journal of Geriatric Psychiatry and Neurology 9: 83–90. 8736588 10.1177/089198879600900203

[cph470089-bib-0088] Draper, D. O. , S. T. Harris , S. Schulthies , E. Durrant , K. L. Knight , and M. Ricard . 1998. “Hot‐Pack and 1‐MHz Ultrasound Treatments Have an Additive Effect on Muscle Temperature Increase.” Journal of Athletic Training 33: 21–24. 16558479 PMC1320370

[cph470089-bib-0089] Draper, D. O. , A. R. Hawkes , A. W. Johnson , M. T. Diede , and J. H. Rigby . 2013. “Muscle Heating With Megapulse II Shortwave Diathermy and ReBound Diathermy.” Journal of Athletic Training 48: 477–482. 23725462 10.4085/1062-6050-48.3.01 PMC3718350

[cph470089-bib-0090] Draper, D. O. , K. Knight , T. Fujiwara , and J. C. Castel . 1999. “Temperature Change in Human Muscle During and After Pulsed Short‐Wave Diathermy.” Journal of Orthopaedic and Sports Physical Therapy 29: 13–22. 10100117 10.2519/jospt.1999.29.1.13

[cph470089-bib-0091] Drew, B. G. , V. Ribas , J. A. Le , et al. 2014. “HSP72 Is a Mitochondrial Stress Sensor Critical for Parkin Action, Oxidative Metabolism, and Insulin Sensitivity in Skeletal Muscle.” Diabetes 63: 1488–1505. 24379352 10.2337/db13-0665 PMC3994950

[cph470089-bib-0092] Ekstrand, J. , M. Hägglund , and M. Waldén . 2011. “Epidemiology of Muscle Injuries in Professional Football (Soccer).” American Journal of Sports Medicine 39: 1226–1232. 21335353 10.1177/0363546510395879

[cph470089-bib-0093] Ely, B. R. , Z. S. Clayton , C. E. McCurdy , et al. 2019. “Heat Therapy Improves Glucose Tolerance and Adipose Tissue Insulin Signaling in Polycystic Ovary Syndrome.” American Journal of Physiology. Endocrinology and Metabolism 317: E172–E182. 31136202 10.1152/ajpendo.00549.2018 PMC7199222

[cph470089-bib-0094] Ely, B. R. , M. A. Francisco , J. R. Halliwill , et al. 2019. “Heat Therapy Reduces Sympathetic Activity and Improves Cardiovascular Risk Profile in Women Who Are Obese With Polycystic Ovary Syndrome.” American Journal of Physiology. Regulatory, Integrative and Comparative Physiology 317: R630–R640. 31483156 10.1152/ajpregu.00078.2019 PMC8424543

[cph470089-bib-0095] Engelland, R. E. , H. W. Hemingway , O. G. Tomasco , A. H. Olivencia‐Yurvati , and S. A. Romero . 2020. “Neural Control of Blood Pressure Is Altered Following Isolated Leg Heating in Aged Humans.” American Journal of Physiology. Heart and Circulatory Physiology 318: H976–H984. 32142377 10.1152/ajpheart.00019.2020 PMC7191488

[cph470089-bib-0096] Engstrom, A. , H. Hagglund , E. Lee , M. Wennberg , S. Soderberg , and M. Andersson . 2024. “Sauna Bathing in Northern Sweden: Results From the MONICA Study 2022.” International Journal of Circumpolar Health 83: 2419698. 39446139 10.1080/22423982.2024.2419698 PMC11524357

[cph470089-bib-0097] Fang, E. F. , H. Kassahun , D. L. Croteau , et al. 2016. “NAD(+) Replenishment Improves Lifespan and Healthspan in Ataxia Telangiectasia Models via Mitophagy and DNA Repair.” Cell Metabolism 24: 566–581. 27732836 10.1016/j.cmet.2016.09.004 PMC5777858

[cph470089-bib-0098] Fang, E. F. , M. Scheibye‐Knudsen , L. E. Brace , et al. 2014. “Defective Mitophagy in XPA via PARP‐1 Hyperactivation and NAD(+)/SIRT1 Reduction.” Cell 157: 882–896. 24813611 10.1016/j.cell.2014.03.026 PMC4625837

[cph470089-bib-0099] Faulkner, S. H. , S. Jackson , G. Fatania , and C. A. Leicht . 2017. “The Effect of Passive Heating on Heat Shock Protein 70 and Interleukin‐6: A Possible Treatment Tool for Metabolic Diseases?” Temperature (Austin) 4: 292–304. 28944271 10.1080/23328940.2017.1288688 PMC5605168

[cph470089-bib-0100] Feder, M. E. , and G. E. Hofmann . 1999. “Heat‐Shock Proteins, Molecular Chaperones, And The Stress Response: Evolutionary and Ecological Physiology.” Annual Review of Physiology 61: 243–282. 10.1146/annurev.physiol.61.1.243 10099689

[cph470089-bib-0101] Fennel, Z. J. , F. T. Amorim , M. R. Deyhle , P. S. Hafen , and C. M. Mermier . 2022. “The Heat Shock Connection: Skeletal Muscle Hypertrophy and Atrophy.” American Journal of Physiology. Regulatory, Integrative and Comparative Physiology 323: R133–R148. 35536704 10.1152/ajpregu.00048.2022

[cph470089-bib-0102] Fieger, S. M. 2011. “Adenosine Receptors in Cutaneous Thermal Hyperemia and Active Vasodilation in Humans.”

[cph470089-bib-0103] Firbank, M. J. , R. M. Wiseman , E. J. Burton , B. K. Saxby , J. T. O'Brien , and G. A. Ford . 2007. “Brain Atrophy and White Matter Hyperintensity Change in Older Adults and Relationship to Blood Pressure. Brain Atrophy, WMH Change and Blood Pressure.” Journal of Neurology 254: 713–721. 17446997 10.1007/s00415-006-0238-4

[cph470089-bib-0104] Fisher‐Wellman, K. H. , and P. D. Neufer . 2012. “Linking Mitochondrial Bioenergetics to Insulin Resistance via Redox Biology.” Trends in Endocrinology and Metabolism 23: 142–153. 22305519 10.1016/j.tem.2011.12.008 PMC3313496

[cph470089-bib-0105] Flack, J. M. , and B. Adekola . 2020. “Blood Pressure and the New ACC/AHA Hypertension Guidelines.” Trends in Cardiovascular Medicine 30: 160–164. 31521481 10.1016/j.tcm.2019.05.003

[cph470089-bib-0106] Fox, R. H. , R. Goldsmith , D. J. Kidd , and H. E. Lewis . 1963a. “Blood Flow and Other Thermoregulatory Changes With Acclimatization to Heat.” Journal of Physiology 166: 548–562. 13959045 10.1113/jphysiol.1963.sp007122 PMC1359352

[cph470089-bib-0107] Fox, R. H. , R. Goldsmith , D. J. Kidd , and H. E. Lewis . 1963b. “Acclimatization to Heat in Man by Controlled Elevation of Body Temperature.” Journal of Physiology 166: 530–547. 13959046 10.1113/jphysiol.1963.sp007121 PMC1359351

[cph470089-bib-0108] Francisco, M. A. , V. E. Brunt , K. N. Jensen , S. Lorenzo , and C. T. Minson . 2017. “Ten Days of Repeated Local Forearm Heating Does Not Affect Cutaneous Vascular Function.” Journal of Applied Physiology 123: 310–316. 28473615 10.1152/japplphysiol.00966.2016 PMC6157477

[cph470089-bib-0109] Frydman, J. 2001. “Folding of Newly Translated Proteins in Vivo: The Role of Molecular Chaperones.” Annual Review of Biochemistry 70: 603–647. 10.1146/annurev.biochem.70.1.603 11395418

[cph470089-bib-0110] Fu, Q. , and S. Ogoh . 2019. “Sex Differences in Baroreflex Function in Health and Disease.” Journal of Physiological Sciences 69: 851–859. 10.1007/s12576-019-00727-z PMC10717578 31721084

[cph470089-bib-0112] Fuchs, C. J. , M. W. Betz , H. L. Petrick , et al. 2025. “Repeated Passive Heat Treatment Increases Muscle Tissue Capillarization, but Does Not Affect Postprandial Muscle Protein Synthesis Rates in Healthy Older Adults.” Journal of Physiology 603: 167–186. 39373667 10.1113/JP286986 PMC11702915

[cph470089-bib-0113] Fuchs, C. J. , J. S. J. Smeets , J. M. Senden , et al. 2020. “Hot‐Water Immersion Does Not Increase Postprandial Muscle Protein Synthesis Rates During Recovery From Resistance‐Type Exercise in Healthy, Young Males.” Journal of Applied Physiology 128: 1012–1022. 32191599 10.1152/japplphysiol.00836.2019

[cph470089-bib-0114] Fujii, N. , T. Amano , L. Halili , et al. 2017. “Intradermal Administration of Endothelin‐1 Attenuates Endothelium‐Dependent and ‐Independent Cutaneous Vasodilation via Rho Kinase in Young Adults.” American Journal of Physiology. Regulatory, Integrative and Comparative Physiology 312: R23–R30. 27881399 10.1152/ajpregu.00368.2016 PMC5283942

[cph470089-bib-0115] Fujii, N. , V. E. Brunt , and C. T. Minson . 2014. “Tempol Improves Cutaneous Thermal Hyperemia Through Increasing Nitric Oxide Bioavailability in Young Smokers.” American Journal of Physiology. Heart and Circulatory Physiology 306: H1507–H1511. 24682395 10.1152/ajpheart.00886.2013 PMC4042197

[cph470089-bib-0116] Fujii, N. , S. Dervis , R. J. Sigal , and G. P. Kenny . 2016. “Type 1 Diabetes Modulates Cyclooxygenase‐ and Nitric Oxide‐Dependent Mechanisms Governing Sweating but Not Cutaneous Vasodilation During Exercise in the Heat.” American Journal of Physiology. Regulatory, Integrative and Comparative Physiology 311: R1076–R1084. 27733388 10.1152/ajpregu.00376.2016

[cph470089-bib-0117] Fujii, N. , L. Halili , T. Nishiyasu , and G. P. Kenny . 2018. “Voltage‐Gated Potassium Channels and NOS Contribute to a Sustained Cutaneous Vasodilation Elicited by Local Heating in an Interactive Manner in Young Adults.” Microvascular Research 117: 22–27. 29247720 10.1016/j.mvr.2017.12.001

[cph470089-bib-0118] Fujii, N. , L. Halili , M. S. Singh , R. D. Meade , and G. P. Kenny . 2015. “Intradermal Administration of ATP Augments Methacholine‐Induced Cutaneous Vasodilation but Not Sweating in Young Males and Females.” American Journal of Physiology. Regulatory, Integrative and Comparative Physiology 309: R912–R919. 26290105 10.1152/ajpregu.00261.2015 PMC4666942

[cph470089-bib-0119] Fujii, N. , R. McGinn , L. Halili , M. S. Singh , N. Kondo , and G. P. Kenny . 2015. “Cutaneous Vascular and Sweating Responses to Intradermal Administration of ATP: A Role for Nitric Oxide Synthase and Cyclooxygenase?” Journal of Physiology 593: 2515–2525. 25809194 10.1113/JP270147 PMC4461412

[cph470089-bib-0120] Fujii, N. , B. D. McNeely , and G. P. Kenny . 2017. “Nitric Oxide Synthase and Cyclooxygenase Modulate β‐Adrenergic Cutaneous Vasodilatation and Sweating in Young Men.” Journal of Physiology 595: 1173–1184. 27779753 10.1113/JP273502 PMC5309368

[cph470089-bib-0121] Fujii, N. , B. D. McNeely , T. Nishiyasu , and G. P. Kenny . 2017. “Intradermal Administration of Atrial Natriuretic Peptide Has no Effect on Sweating and Cutaneous Vasodilator Responses in Young Male Adults.” Temperature 4: 406–413. 10.1080/23328940.2017.1356433 PMC5800362 29435479

[cph470089-bib-0122] Fujii, N. , R. D. Meade , P. Akbari , et al. 2017. “No Effect of Ascorbate on Cutaneous Vasodilation and Sweating in Older Men and Those With Type 2 Diabetes Exercising in the Heat.” Physiological Reports 5: e13238. 28400505 10.14814/phy2.13238 PMC5392524

[cph470089-bib-0123] Fujii, N. , R. D. Meade , L. M. Alexander , et al. 2016. “iNOS‐Dependent Sweating and eNOS‐Dependent Cutaneous Vasodilation Are Evident in Younger Adults, but Are Diminished in Older Adults Exercising in the Heat.” Journal of Applied Physiology (Bethesda, MD: 1985) 120: 318–327. 26586908 10.1152/japplphysiol.00714.2015 PMC4740499

[cph470089-bib-0124] Fujii, N. , O. L. Pastore , G. W. McGarr , et al. 2018. “Cyclooxygenase‐1 and ‐2 Modulate Sweating but Not Cutaneous Vasodilation During Exercise in the Heat in Young Men.” Physiological Reports 6: e13844. 30175553 10.14814/phy2.13844 PMC6119687

[cph470089-bib-0125] Fujii, N. , G. Paull , R. D. Meade , et al. 2015. “Do Nitric Oxide Synthase and Cyclooxygenase Contribute to the Heat Loss Responses in Older Males Exercising in the Heat?” Journal of Physiology 593: 3169–3180. 25820454 10.1113/JP270330 PMC4532535

[cph470089-bib-0126] Fujita, S. , Y. Ikeda , M. Miyata , et al. 2011. “Effect of Waon Therapy on Oxidative Stress in Chronic Heart Failure.” Circulation Journal 75: 348–356. 21173495 10.1253/circj.cj-10-0630

[cph470089-bib-0127] Gagnon, D. , S. A. Romero , H. Ngo , et al. 2016. “Healthy Aging Does Not Compromise the Augmentation of Cardiac Function During Heat Stress.” Journal of Applied Physiology (Bethesda, MD: 1985) 121: 885–892. 27609201 10.1152/japplphysiol.00643.2016 PMC5142306

[cph470089-bib-0128] Gagnon, D. , S. A. Romero , H. Ngo , et al. 2017. “Volume Loading Augments Cutaneous Vasodilatation and Cardiac Output of Heat Stressed Older Adults.” Journal of Physiology 595: 6489–6498. 28833129 10.1113/JP274742 PMC5638885

[cph470089-bib-0129] Ganio, M. S. , R. M. Brothers , S. Shibata , J. L. Hastings , and C. G. Crandall . 2011. “Effect of Passive Heat Stress on Arterial Stiffness.” Experimental Physiology 96: 919–926. 21685446 10.1113/expphysiol.2011.057091 PMC3162119

[cph470089-bib-0130] García‐Cardeña, G. , R. Fan , V. Shah , et al. 1998. “Dynamic Activation of Endothelial Nitric Oxide Synthase by Hsp90.” Nature 392: 821–824. 9580552 10.1038/33934

[cph470089-bib-0131] Gayda, M. , L. Bosquet , F. Paillard , et al. 2012. “Effects of Sauna Alone Versus Postexercise Sauna Baths on Short‐Term Heart Rate Variability in Patients With Untreated Hypertension.” Journal of Cardiopulmonary Rehabilitation and Prevention 32: 147–154. 22561416 10.1097/HCR.0b013e318251ffeb

[cph470089-bib-0132] Gayda, M. , F. Paillard , P. Sosner , et al. 2012. “Effects of Sauna Alone and Postexercise Sauna Baths on Blood Pressure and Hemodynamic Variables in Patients With Untreated Hypertension.” Journal of Clinical Hypertension 14: 553–560. 22863164 10.1111/j.1751-7176.2012.00637.x PMC8108777

[cph470089-bib-0133] Gianaros, P. J. , P. J. Greer , C. M. Ryan , and J. R. Jennings . 2006. “Higher Blood Pressure Predicts Lower Regional Grey Matter Volume: Consequences on Short‐Term Information Processing.” NeuroImage 31: 754–765. 16488626 10.1016/j.neuroimage.2006.01.003 PMC2254305

[cph470089-bib-0134] Giombini, A. , G. Casciello , M. C. Di Cesare , A. Di Cesare , S. Dragoni , and D. Sorrenti . 2001. “A Controlled Study on the Effects of Hyperthermia at 434 MHz and Conventional Ultrasound Upon Muscle Injuries in Sport.” Journal of Sports Medicine and Physical Fitness 41: 521–527. 11687773

[cph470089-bib-0135] Glodzik, L. , L. Mosconi , W. Tsui , et al. 2012. “Alzheimer's Disease Markers, Hypertension, and Gray Matter Damage in Normal Elderly.” Neurobiology of Aging 33: 1215–1227. 21530003 10.1016/j.neurobiolaging.2011.02.012 PMC3179821

[cph470089-bib-0136] Goats, G. C. 1989a. “Continuous Short‐Wave (Radio‐Frequency) Diathermy.” British Journal of Sports Medicine 23: 123–127. 2691003 10.1136/bjsm.23.2.123 PMC1478624

[cph470089-bib-0137] Goats, G. C. 1989b. “Pulsed Electromagnetic (Short‐Wave) Energy Therapy.” British Journal of Sports Medicine 23: 213–216. 2629997 10.1136/bjsm.23.4.213 PMC1478698

[cph470089-bib-0138] Goto, K. , H. Oda , H. Kondo , et al. 2011. “Responses of Muscle Mass, Strength and Gene Transcripts to Long‐Term Heat Stress in Healthy Human Subjects.” European Journal of Applied Physiology 111: 17–27. 20803152 10.1007/s00421-010-1617-1

[cph470089-bib-0139] Goto, K. , R. Okuyama , H. Sugiyama , et al. 2003. “Effects of Heat Stress and Mechanical Stretch on Protein Expression in Cultured Skeletal Muscle Cells.” Pflügers Archiv 447: 247–253. 14534791 10.1007/s00424-003-1177-x

[cph470089-bib-0140] Gowda, A. , C.‐J. Yang , G. K. Asimakis , et al. 1998. “Cardioprotection by Local Heating: Improved Myocardial Salvage After Ischemia and Reperfusion.” Annals of Thoracic Surgery 65: 1241–1247. 9594845 10.1016/s0003-4975(98)00117-9

[cph470089-bib-0141] Gratton, J.‐P. , J. Fontana , D. S. O'Connor , G. García‐Cardeña , T. J. McCabe , and W. C. Sessa . 2000. “Reconstitution of an Endothelial Nitric‐Oxide Synthase (eNOS), hsp90, and Caveolin‐1 Complex In Vitro: Evidence That Hsp90 Facilitates Calmodulin Stimulated Displacement of eNOS FROM CAVEOLIN‐1.” Journal of Biological Chemistry 275: 22268–22272. 10781589 10.1074/jbc.M001644200

[cph470089-bib-0142] Green, D. J. , H. H. Carter , M. G. Fitzsimons , N. T. Cable , D. H. J. Thijssen , and L. H. Naylor . 2010. “Obligatory Role of Hyperaemia and Shear Stress in Microvascular Adaptation to Repeated Heating in Humans.” Journal of Physiology 588: 1571–1577. 20211982 10.1113/jphysiol.2010.186965 PMC2876810

[cph470089-bib-0143] Green, D. J. , H. Jones , D. Thijssen , N. T. Cable , and G. Atkinson . 2011. “Flow‐Mediated Dilation and Cardiovascular Event Prediction.” Hypertension 57: 363–369. 21263128 10.1161/HYPERTENSIONAHA.110.167015

[cph470089-bib-0144] Guo, J. , L. Li , Y. Gong , et al. 2017. “Massage Alleviates Delayed Onset Muscle Soreness After Strenuous Exercise: A Systematic Review and Meta‐Analysis.” Frontiers in Physiology 8: 747. 29021762 10.3389/fphys.2017.00747 PMC5623674

[cph470089-bib-0145] Gupte, A. A. , G. L. Bomhoff , R. H. Swerdlow , and P. C. Geiger . 2009. “Heat Treatment Improves Glucose Tolerance and Prevents Skeletal Muscle Insulin Resistance in Rats Fed a High‐Fat Diet.” Diabetes 58: 567–578. 19073766 10.2337/db08-1070 PMC2646055

[cph470089-bib-0146] Gupte, A. A. , G. L. Bomhoff , C. D. Touchberry , and P. C. Geiger . 2011. “Acute Heat Treatment Improves Insulin‐Stimulated Glucose Uptake in Aged Skeletal Muscle.” Journal of Applied Physiology (Bethesda, MD: 1985) 110: 451–457. 21148343 10.1152/japplphysiol.00849.2010 PMC3043783

[cph470089-bib-0147] Hafen, P. S. , K. Abbott , J. Bowden , R. Lopiano , C. R. Hancock , and R. D. Hyldahl . 2019. “Daily Heat Treatment Maintains Mitochondrial Function and Attenuates Atrophy in Human Skeletal Muscle Subjected to Immobilization.” Journal of Applied Physiology (Bethesda, MD: 1985) 127: 47–57. 31046520 10.1152/japplphysiol.01098.2018

[cph470089-bib-0148] Hafen, P. S. , C. N. Preece , J. R. Sorensen , C. R. Hancock , and R. D. Hyldahl . 2018. “Repeated Exposure to Heat Stress Induces Mitochondrial Adaptation in Human Skeletal Muscle.” Journal of Applied Physiology (Bethesda, MD: 1985) 125: 1447–1455. 30024339 10.1152/japplphysiol.00383.2018

[cph470089-bib-0149] Halili, L. , M. S. Singh , N. Fujii , L. M. Alexander , and G. P. Kenny . 2016. “Endothelin‐1 Modulates Methacholine‐Induced Cutaneous Vasodilatation but Not Sweating in Young Human Skin.” Journal of Physiology 594: 3439–3452. 26846374 10.1113/JP271735 PMC4908015

[cph470089-bib-0150] Hands, S. , C. Sinadinos , and A. Wyttenbach . 2008. “Polyglutamine Gene Function and Dysfunction in the Ageing Brain.” Biochimica et Biophysica Acta 1779: 507–521. 18582603 10.1016/j.bbagrm.2008.05.008

[cph470089-bib-0151] Harris, M. B. , M. A. Blackstone , H. Ju , V. J. Venema , and R. C. Venema . 2003. “Heat‐Induced Increases in Endothelial NO Synthase Expression and Activity and Endothelial NO Release.” American Journal of Physiology. Heart and Circulatory Physiology 285: H333–H340. 12663266 10.1152/ajpheart.00726.2002

[cph470089-bib-0152] Hartl, F. U. , A. Bracher , and M. Hayer‐Hartl . 2011. “Molecular Chaperones in Protein Folding and Proteostasis.” Nature 475: 324–332. 21776078 10.1038/nature10317

[cph470089-bib-0153] Harwood, A. E. , C. J. Pugh , C. J. Steward , C. Menzies , C. D. Thake , and T. Cullen . 2021. “A Systematic Review of the Role of Heat Therapy for Patients With Intermittent Claudication due to Peripheral Artery Disease.” Vascular Medicine 26: 440–447. 33587690 10.1177/1358863X20983475 PMC8358540

[cph470089-bib-0154] Hashizaki, T. , Y. Nishimura , K. Teramura , et al. 2018. “Differences in Serum IL‐6 Response After 1 Degrees C Rise in Core Body Temperature in Individuals With Spinal Cord Injury and Cervical Spinal Cord Injury During Local Heat Stress.” International Journal of Hyperthermia 35: 541–547. 30303416 10.1080/02656736.2018.1511838

[cph470089-bib-0155] Heinonen, I. , and J. A. Laukkanen . 2018. “Effects of Heat and Cold on Health, With Special Reference to Finnish Sauna Bathing.” American Journal of Physiology. Regulatory, Integrative and Comparative Physiology 314: R629–R638. 29351426 10.1152/ajpregu.00115.2017

[cph470089-bib-0156] Hemingway, H. W. , R. E. Richey , A. M. Moore , A. H. Olivencia‐Yurvati , G. P. Kline , and S. A. Romero . 2022. “Acute Heat Exposure Protects Against Endothelial Ischemia‐Reperfusion Injury in Aged Humans.” American Journal of Physiology. Regulatory, Integrative and Comparative Physiology 322: R360–R367. 35200050 10.1152/ajpregu.00336.2021 PMC8993535

[cph470089-bib-0157] Henstridge, D. C. , C. R. Bruce , B. G. Drew , et al. 2014. “Activating HSP72 in Rodent Skeletal Muscle Increases Mitochondrial Number and Oxidative Capacity and Decreases Insulin Resistance.” Diabetes 63: 1881–1894. 24430435 10.2337/db13-0967 PMC4030108

[cph470089-bib-0158] Henstridge, D. C. , J. M. Forbes , S. A. Penfold , et al. 2010. “The Relationship Between Heat Shock Protein 72 Expression in Skeletal Muscle and Insulin Sensitivity Is Dependent on Adiposity.” Metabolism 59: 1556–1561. 20199785 10.1016/j.metabol.2010.01.027

[cph470089-bib-0159] Hesketh, K. , S. O. Shepherd , J. A. Strauss , et al. 2019. “Passive Heat Therapy in Sedentary Humans Increases Skeletal Muscle Capillarization and eNOS Content but Not Mitochondrial Density or GLUT4 Content.” American Journal of Physiology. Heart and Circulatory Physiology 317: H114–H123. 31074654 10.1152/ajpheart.00816.2018

[cph470089-bib-0160] Hetz, C. , and S. Saxena . 2017. “ER Stress and the Unfolded Protein Response in Neurodegeneration.” Nature Reviews. Neurology 13: 477–491. 28731040 10.1038/nrneurol.2017.99

[cph470089-bib-0161] Hewett, Z. L. , B. S. Cheema , K. L. Pumpa , and C. A. Smith . 2015. “The Effects of Bikram Yoga on Health: Critical Review and Clinical Trial Recommendations.” Evidence‐based Complementary and Alternative Medicine 2015: 1–13. 10.1155/2015/428427 PMC4609431 26504475

[cph470089-bib-0162] Hewett, Z. L. , K. L. Pumpa , C. A. Smith , P. P. Fahey , and B. S. Cheema . 2017. “Effect of a 16‐Week Bikram Yoga Program on Heart Rate Variability and Associated Cardiovascular Disease Risk Factors in Stressed and Sedentary Adults: A Randomized Controlled Trial.” BMC Complementary and Alternative Medicine 17: 226. 28431533 10.1186/s12906-017-1740-1 PMC5399826

[cph470089-bib-0163] Hill, A. B. 1965. “The Environment and Disease: Association of Causation?” Proceedings of the Royal Society of Medicine 58: 295–300. 14283879 10.1177/003591576505800503 PMC1898525

[cph470089-bib-0164] Hoekstra, S. P. , N. C. Bishop , S. H. Faulkner , S. J. Bailey , and C. A. Leicht . 2018. “Acute and Chronic Effects of Hot Water Immersion on Inflammation and Metabolism in Sedentary, Overweight Adults.” Journal of Applied Physiology (Bethesda, MD: 1985) 125: 2008–2018. 30335579 10.1152/japplphysiol.00407.2018

[cph470089-bib-0165] Holloszy, J. O. 1967. “Biochemical Adaptations in Muscle. Effects of Exercise on Mitochondrial Oxygen Uptake and Respiratory Enzyme Activity in Skeletal Muscle.” Journal of Biological Chemistry 242: 2278–2282. 4290225

[cph470089-bib-0166] Honea, R. A. , C. S. John , Z. D. Green , et al. 2022. “Relationship of Fasting Glucose and Longitudinal Alzheimer's Disease Imaging Markers.” Alzheimers Dement 8: e12239. 10.1002/trc2.12239 PMC8804928 35128029

[cph470089-bib-0167] Hood, D. A. , J. M. Memme , A. N. Oliveira , and M. Triolo . 2019. “Maintenance of Skeletal Muscle Mitochondria in Health, Exercise, and Aging.” Annual Review of Physiology 81: 19–41. 10.1146/annurev-physiol-020518-114310 30216742

[cph470089-bib-0168] Hooper, C. , R. Killick , and S. Lovestone . 2008. “The GSK3 Hypothesis of Alzheimer's Disease.” Journal of Neurochemistry 104: 1433–1439. 18088381 10.1111/j.1471-4159.2007.05194.x PMC3073119

[cph470089-bib-0169] Hooper, P. L. 1999. “Hot‐Tub Therapy for Type 2 Diabetes Mellitus.” New England Journal of Medicine 341: 924–925. 10.1056/NEJM199909163411216 10498473

[cph470089-bib-0170] Hooper, P. L. , G. Balogh , E. Rivas , K. Kavanagh , and L. Vigh . 2014. “The Importance of the Cellular Stress Response in the Pathogenesis and Treatment of Type 2 Diabetes.” Cell Stress & Chaperones 19: 447–464. 24523032 10.1007/s12192-014-0493-8 PMC4041942

[cph470089-bib-0171] Horne, J. A. , and A. J. Reid . 1985. “Night‐Time Sleep EEG Changes Following Body Heating in a Warm Bath.” Electroencephalography and Clinical Neurophysiology 60: 154–157. 2578367 10.1016/0013-4694(85)90022-7

[cph470089-bib-0172] Hotamisligil, G. S. 2006. “Inflammation and Metabolic Disorders.” Nature 444: 860–867. 17167474 10.1038/nature05485

[cph470089-bib-0173] Houle, M. S. , and G. E. Billman . 1999. “Low‐Frequency Component of the Heart Rate Variability Spectrum: A Poor Marker of Sympathetic Activity.” American Journal of Physiology. Heart and Circulatory Physiology 276: H215–H223. 10.1152/ajpheart.1999.276.1.H215 9887035

[cph470089-bib-0174] Hsu, A. L. , C. T. Murphy , and C. Kenyon . 2003. “Regulation of Aging and Age‐Related Disease by DAF‐16 and Heat‐Shock Factor.” Science 300: 1142–1145. 12750521 10.1126/science.1083701

[cph470089-bib-0175] “Contributors W. Hot tub Wikipedia.” https://en.wikipedia.org/wiki/Hot_tub [07.31.2025, 2025].

[cph470089-bib-0176] Hu, C. , J. Yang , Z. Qi , et al. 2022. “Heat Shock Proteins: Biological Functions, Pathological Roles, and Therapeutic Opportunities.” MedComm (2020) 3: e161. 35928554 10.1002/mco2.161 PMC9345296

[cph470089-bib-0177] Hu, Q. , W. Zhu , Y. Zhu , L. Zheng , and R. L. Hughson . 2012. “Acute Effects of Warm Footbath on Arterial Stiffness in Healthy Young and Older Women.” European Journal of Applied Physiology 112: 1261–1268. 21833487 10.1007/s00421-011-2066-1

[cph470089-bib-0178] Hunter, S. D. , M. Dhindsa , E. Cunningham , T. Tarumi , M. Alkatan , and H. Tanaka . 2013. “Improvements in Glucose Tolerance With Bikram Yoga in Older Obese Adults: A Pilot Study.” Journal of Bodywork and Movement Therapies 17: 404–407. 24138995 10.1016/j.jbmt.2013.01.002

[cph470089-bib-0179] Hunter, S. D. , M. S. Dhindsa , E. Cunningham , et al. 2013. “The Effect of Bikram Yoga on Arterial Stiffness in Young and Older Adults.” Journal of Alternative and Complementary Medicine 19: 930–934. 23738677 10.1089/acm.2012.0709

[cph470089-bib-0180] Hunter, S. D. , M. S. Dhindsa , E. Cunningham , et al. 2017. “The Effect of Bikram Yoga on Endothelial Function in Young and Middle‐Aged and Older Adults.” Journal of Bodywork and Movement Therapies 21: 30–34. 28167186 10.1016/j.jbmt.2016.06.004

[cph470089-bib-0181] Hunter, S. D. , M. S. Dhindsa , E. Cunningham , et al. 2016. “Impact of Hot Yoga on Arterial Stiffness and Quality of Life in Overweight/Obese Adults.” Journal of Physical Activity and Health 13: 1360–1363. 27633625 10.1123/jpah.2016-0170

[cph470089-bib-0182] Hunter, S. D. , S. A. Kavouras , M. Rahimi , S. D. Hunter , S. A. Kavouras , and M. Rahimi . 2023. “Exploring Heated Exercise as a Means of Preventing the Deleterious Effects of High‐Sodium Intake in Black Women.” American Journal of Physiology 324: H833–H839. 37027326 10.1152/ajpheart.00699.2022

[cph470089-bib-0183] Hunter, S. D. , J. Laosiripisan , A. Elmenshawy , and H. Tanaka . 2018. “Effects of Yoga Interventions Practised in Heated and Thermoneutral Conditions on Endothelium‐Dependent Vasodilatation: The Bikram Yoga Heart Study.” Experimental Physiology 103: 391–396. 29349832 10.1113/EP086725

[cph470089-bib-0184] Hussain, J. N. , R. F. Greaves , and M. M. Cohen . 2019. “A Hot Topic for Health: Results of the Global Sauna Survey.” Complementary Therapies in Medicine 44: 223–234. 31126560 10.1016/j.ctim.2019.03.012

[cph470089-bib-0185] Hyldahl, R. D. , P. S. Hafen , W. B. Nelson , et al. 2021. “Passive Muscle Heating Attenuates the Decline in Vascular Function Caused by Limb Disuse.” Journal of Physiology 599: 4581–4596. 34487346 10.1113/JP281900

[cph470089-bib-0186] Iguchi, M. , A. E. Littmann , S. H. Chang , L. A. Wester , J. S. Knipper , and R. K. Shields . 2012. “Heat Stress and Cardiovascular, Hormonal, and Heat Shock Proteins in Humans.” Journal of Athletic Training 47: 184–190. 22488284 10.4085/1062-6050-47.2.184 PMC3418130

[cph470089-bib-0187] Ihsan, M. , L. Deldicque , J. Molphy , F. Britto , A. Cherif , and S. Racinais . 2020. “Skeletal Muscle Signaling Following Whole‐Body and Localized Heat Exposure in Humans.” Frontiers in Physiology 11: 839. 32765299 10.3389/fphys.2020.00839 PMC7381176

[cph470089-bib-0188] Imamura, M. , S. Biro , T. Kihara , et al. 2001. “Repeated Thermal Therapy Improves Impaired Vascular Endothelial Function in Patients With Coronary Risk Factors.” Journal of the American College of Cardiology 38: 1083–1088. 11583886 10.1016/s0735-1097(01)01467-x

[cph470089-bib-0189] Intlekofer, K. A. , and C. W. Cotman . 2013. “Exercise Counteracts Declining Hippocampal Function in Aging and Alzheimer's Disease.” Neurobiology of Disease 57: 47–55. 22750524 10.1016/j.nbd.2012.06.011

[cph470089-bib-0190] Jackman, J. S. , P. G. Bell , K. Van Someren , et al. 2023. “Effect of Hot Water Immersion on Acute Physiological Responses Following Resistance Exercise.” Frontiers in Physiology 14: 1213733. 37476688 10.3389/fphys.2023.1213733 PMC10354234

[cph470089-bib-0191] James, T. J. , J. Corbett , M. Cummings , et al. 2024. “The Effect of Repeated Hot Water Immersion on Vascular Function, Blood Pressure and Central Haemodynamics in Individuals With Type 2 Diabetes Mellitus.” Journal of Thermal Biology 126: 104017. 39642665 10.1016/j.jtherbio.2024.104017

[cph470089-bib-0192] James, T. J. , J. Corbett , M. Cummings , et al. 2023. “The Effect of Repeated Hot Water Immersion on Insulin Sensitivity, Heat Shock Protein 70, and Inflammation in Individuals With Type 2 Diabetes Mellitus.” American Journal of Physiology. Endocrinology and Metabolism 325: E755–E763. 37938179 10.1152/ajpendo.00222.2023

[cph470089-bib-0193] Janson, J. , T. Laedtke , J. E. Parisi , P. O'Brien , R. C. Petersen , and P. C. Butler . 2004. “Increased Risk of Type 2 Diabetes in Alzheimer Disease.” Diabetes 53: 474–481. 14747300 10.2337/diabetes.53.2.474

[cph470089-bib-0194] Janssen, C. W. , C. A. Lowry , M. R. Mehl , et al. 2016. “Whole‐Body Hyperthermia for the Treatment of Major Depressive Disorder: A Randomized Clinical Trial.” JAMA Psychiatry 73: 789–795. 27172277 10.1001/jamapsychiatry.2016.1031

[cph470089-bib-0195] Järvinen, T. A. , M. Järvinen , and H. Kalimo . 2013. “Regeneration of Injured Skeletal Muscle After the Injury.” Muscles Ligaments and Tendons Journal 3: 337–345. 24596699 PMC3940509

[cph470089-bib-0196] Jiang, Y. , R. He , Y. Shi , J. Liang , and L. Zhao . 2020. “Plasma Exosomes Protect Against Cerebral Ischemia/Reperfusion Injury via Exosomal HSP70 Mediated Suppression of ROS.” Life Sciences 256: 117987. 32569778 10.1016/j.lfs.2020.117987

[cph470089-bib-0197] Joannides, R. , A. Costentin , M. Iacob , E.‐H. Bakkali , M.‐O. Richard , and C. Thuillez . 2001. “Role of Arterial Smooth Muscle Tone and Geometry in the Regulation of Peripheral Conduit Artery Mechanics by Shear Stress.” Clinical and Experimental Pharmacology and Physiology 28: 1025–1031. 11903308 10.1046/j.1440-1681.2001.03594.x

[cph470089-bib-0198] Johnson, C. N. , C. R. Lysaker , E. C. Gast , et al. 2025. “APOE4 Exerts Partial Diet‐Dependent Effects on Energy Expenditure and Skeletal Muscle Mitochondrial Pathways in a Preclinical Model.” Function (Oxf) 6: 2025. 10.1093/function/zqaf017 PMC11980864 40133005

[cph470089-bib-0199] Johnson, C. N. , C. S. McCoin , P. J. Kueck , et al. 2023. “Relationship of Muscle Apolipoprotein E Expression With Markers of Cellular Stress, Metabolism, and Blood Biomarkers in Cognitively Healthy and Impaired Older Adults.” Journal of Alzheimer's Disease 92: 1027–1035. 10.3233/JAD-221192 PMC10116140 36847010

[cph470089-bib-0200] Johnson, J. D. , J. Campisi , C. M. Sharkey , S. L. Kennedy , M. Nickerson , and M. Fleshner . 2005. “Adrenergic Receptors Mediate Stress‐Induced Elevations in Extracellular Hsp72.” Journal of Applied Physiology 99: 1789–1795. 16037404 10.1152/japplphysiol.00390.2005

[cph470089-bib-0201] Joyner, M. J. , and D. P. Casey . 2015. “Regulation of Increased Blood Flow (Hyperemia) to Muscles During Exercise: A Hierarchy of Competing Physiological Needs.” Physiological Reviews 95: 549–601. 25834232 10.1152/physrev.00035.2013 PMC4551211

[cph470089-bib-0202] Kaiser, B. W. , L. N. Comrada , B. M. Gibson , et al. 2025. “No Effect of Either Heat Therapy or Aerobic Exercise Training on Blood Pressure in Adults With Untreated Hypertension: A Randomized Clinical Trial.” Journal of Applied Physiology 138: 1600–1614. 40407037 10.1152/japplphysiol.00959.2024 PMC12225054

[cph470089-bib-0203] Kaiser, P. , U. Seeher , A. Krasniqi , A. Keiler , R. Crazzolara , and A. Meryk . 2023. “Injuries Related to Sauna Bathing.” Injury 54: 110825. 37211472 10.1016/j.injury.2023.05.056

[cph470089-bib-0204] Kakigi, R. , H. Naito , Y. Ogura , et al. 2011. “Heat Stress Enhances mTOR Signaling After Resistance Exercise in Human Skeletal Muscle.” Journal of Physiological Sciences 61: 131–140. 10.1007/s12576-010-0130-y PMC10717825 21222186

[cph470089-bib-0205] Kalmar, B. , C. H. Lu , and L. Greensmith . 2014. “The Role of Heat Shock Proteins in Amyotrophic Lateral Sclerosis: The Therapeutic Potential of Arimoclomol.” Pharmacology & Therapeutics 141: 40–54. 23978556 10.1016/j.pharmthera.2013.08.003

[cph470089-bib-0206] Kaluhiokalani, J. P. , T. E. Wallace , M. Ahmadi , et al. 2025. “Six Weeks of Localized Passive Heat Therapy Elicits Some Exercise‐Like Improvements in Resistance Artery Function.” Journal of Physiology 603:5163–5179. 39004886 10.1113/JP286567

[cph470089-bib-0207] Karmazyn, M. , K. Mailer , and R. W. Currie . 1990. “Acquisition and Decay of Heat‐Shock‐Enhanced Postischemic Ventricular Recovery.” American Journal of Physiology. Heart and Circulatory Physiology 259: H424–H431. 10.1152/ajpheart.1990.259.2.H424 2386221

[cph470089-bib-0442] Katsuyama, H. , M. Hakoshima , H. Adachi , et al. 2022. “Habitual Hot‐Tub Bathing and Cardiovascular Risk Factors in Patients With Type 2 Diabetes Mellitus: A Cross‐Sectional Study.” Cardiology Research 13, no. 3: 144–153. 10.14740/cr1371. 35836731 PMC9239506

[cph470089-bib-0208] Katz, L. D. , M. G. Glickman , S. Rapoport , E. Ferrannini , and R. A. DeFronzo . 1983. “Splanchnic and Peripheral Disposal of Oral Glucose in Man.” Diabetes 32: 675–679. 6862113 10.2337/diab.32.7.675

[cph470089-bib-0209] Kaushik, S. , and A. M. Cuervo . 2015. “Proteostasis and Aging.” Nature Medicine 21: 1406–1415. 10.1038/nm.4001 26646497

[cph470089-bib-0210] Kaushik, S. , and A. M. Cuervo . 2018. “The Coming of Age of Chaperone‐Mediated Autophagy.” Nature Reviews. Molecular Cell Biology 19: 365–381. 29626215 10.1038/s41580-018-0001-6 PMC6399518

[cph470089-bib-0211] Kavanagh, K. , A. T. Davis , K. A. Jenkins , and D. M. Flynn . 2016. “Effects of Heated Hydrotherapy on Muscle HSP70 and Glucose Metabolism in Old and Young Vervet Monkeys.” Cell Stress & Chaperones 21: 717–725. 27188431 10.1007/s12192-016-0699-z PMC4908005

[cph470089-bib-0212] Keller, D. M. , M. Sander , B. Stallknecht , and C. G. Crandall . 2010. “α‐Adrenergic Vasoconstrictor Responsiveness Is Preserved in the Heated Human Leg.” Journal of Physiology 588: 3799–3808. 20693291 10.1113/jphysiol.2010.194506 PMC2998227

[cph470089-bib-0213] Kennelly, S. P. , B. A. Lawlor , and R. A. Kenny . 2009. “Blood Pressure and Dementia—A Comprehensive Review.” Therapeutic Advances in Neurological Disorders 2: 241–260. 21179532 10.1177/1756285609103483 PMC3002634

[cph470089-bib-0214] Kenney, W. L. 2017. “Edward F. Adolph Distinguished Lecture: Skin‐Deep Insights Into Vascular Aging.” Journal of Applied Physiology 123, no. 5: 1024–1038. 28729391 10.1152/japplphysiol.00589.2017 PMC5792098

[cph470089-bib-0215] Khurana, V. G. , K. Feterik , M. J. Springett , D. Eguchi , V. Shah , and Z. S. Katusic . 2000. “Functional Interdependence and Colocalization of Endothelial Nitric Oxide Synthase and Heat Shock Protein 90 in Cerebral Arteries.” Journal of Cerebral Blood Flow and Metabolism 20: 1563–1570. 11083231 10.1097/00004647-200011000-00006

[cph470089-bib-0216] Kihara, T. , S. Biro , M. Imamura , et al. 2002. “Repeated Sauna Treatment Improves Vascular Endothelial and Cardiac Function in Patients With Chronic Heart Failure.” Journal of the American College of Cardiology 39: 754–759. 11869837 10.1016/s0735-1097(01)01824-1

[cph470089-bib-0217] Kihara, T. , M. Miyata , T. Fukudome , et al. 2009. “Waon Therapy Improves the Prognosis of Patients With Chronic Heart Failure.” Journal of Cardiology 53: 214–218. 19304125 10.1016/j.jjcc.2008.11.005

[cph470089-bib-0218] Kim, E. , K. Sakata , and F. F. Liao . 2017. “Bidirectional Interplay of HSF1 Degradation and UPR Activation Promotes Tau Hyperphosphorylation.” PLoS Genetics 13: e1006849. 28678786 10.1371/journal.pgen.1006849 PMC5517072

[cph470089-bib-0219] Kim, K. , J. C. Monroe , T. P. Gavin , and B. T. Roseguini . 2020. “Skeletal Muscle Adaptations to Heat Therapy.” Journal of Applied Physiology 128: 1635–1642. 32352340 10.1152/japplphysiol.00061.2020 PMC7311689

[cph470089-bib-0220] Kim, K. , J. C. Monroe , T. P. Gavin , and B. T. Roseguini . 2020. “Local Heat Therapy to Accelerate Recovery After Exercise‐Induced Muscle Damage.” Exercise and Sport Sciences Reviews 48: 163–169. 32658042 10.1249/JES.0000000000000230 PMC7492448

[cph470089-bib-0221] Kim, K. , B. A. Reid , C. A. Casey , et al. 2020. “Effects of Repeated Local Heat Therapy on Skeletal Muscle Structure and Function in Humans.” Journal of Applied Physiology (Bethesda, MD: 1985) 128: 483–492. 31971474 10.1152/japplphysiol.00701.2019

[cph470089-bib-0222] Kinlay, S. , M. A. Creager , M. Fukumoto , et al. 2001. “Endothelium‐Derived Nitric Oxide Regulates Arterial Elasticity in Human Arteries in Vivo.” Hypertension 38: 1049–1053. 11711496 10.1161/hy1101.095329

[cph470089-bib-0223] Kirchner, P. , M. Bourdenx , J. Madrigal‐Matute , et al. 2019. “Proteome‐Wide Analysis of Chaperone‐Mediated Autophagy Targeting Motifs.” PLoS Biology 17: e3000301. 31150375 10.1371/journal.pbio.3000301 PMC6561683

[cph470089-bib-0224] Klaips, C. L. , G. G. Jayaraj , and F. U. Hartl . 2018. “Pathways of Cellular Proteostasis in Aging and Disease.” Journal of Cell Biology 217: 51–63. 29127110 10.1083/jcb.201709072 PMC5748993

[cph470089-bib-0225] Kline, M. P. , and R. I. Morimoto . 1997. “Repression of the Heat Shock Factor 1 Transcriptional Activation Domain Is Modulated by Constitutive Phosphorylation.” Molecular and Cellular Biology 17: 2107–2115. 9121459 10.1128/mcb.17.4.2107 PMC232058

[cph470089-bib-0226] Koçak, F. A. , E. E. Kurt , F. Milletli Sezgin , S. Şaş , F. Tuncay , and H. R. Erdem . 2020. “The Effect of Balneotherapy on Body Mass Index, Adipokine Levels, Sleep Disturbances, and Quality of Life of Women With Morbid Obesity.” International Journal of Biometeorology 64: 1463–1472. 32377931 10.1007/s00484-020-01924-x PMC7223765

[cph470089-bib-0227] Kohara, K. , Y. Tabara , M. Ochi , et al. 2018. “Habitual Hot Water Bathing Protects Cardiovascular Function in Middle‐Aged to Elderly Japanese Subjects.” Scientific Reports 8: 8687. 29930309 10.1038/s41598-018-26908-1 PMC6013438

[cph470089-bib-0228] Kokura, S. , S. Adachi , E. Manabe , et al. 2007. “Whole Body Hyperthermia Improves Obesity‐Induced Insulin Resistance in Diabetic Mice.” International Journal of Hyperthermia 23: 259–265. 17523018 10.1080/02656730601176824

[cph470089-bib-0229] Kominami, K. , K. Noda , N. Takahashi , T. Izumi , and K. Yonezawa . 2020. “Cardiovascular Reactions for Whole‐Body Thermal Therapy With a Hot Pack and Waon Therapy.” International Journal of Hyperthermia 37: 184–191. 32046537 10.1080/02656736.2020.1723719

[cph470089-bib-0230] Koshinaka, K. , E. Kawamoto , N. Abe , K. Toshinai , M. Nakazato , and K. Kawanaka . 2013. “Elevation of Muscle Temperature Stimulates Muscle Glucose Uptake in Vivo and in Vitro.” Journal of Physiological Sciences 63: 409–418. 10.1007/s12576-013-0278-3 PMC10718043 23836025

[cph470089-bib-0231] Krause, M. , M. S. Ludwig , T. G. Heck , and H. K. Takahashi . 2015. “Heat Shock Proteins and Heat Therapy for Type 2 Diabetes: Pros and Cons.” Current Opinion in Clinical Nutrition and Metabolic Care 18: 374–380. 26049635 10.1097/MCO.0000000000000183

[cph470089-bib-0232] Kregel, K. C. 2002. “Heat Shock Proteins: Modifying Factors in Physiological Stress Responses and Acquired Thermotolerance.” Journal of Applied Physiology (Bethesda, MD: 1985) 92: 2177–2186. 11960972 10.1152/japplphysiol.01267.2001

[cph470089-bib-0233] Kuhlenhoelter, A. M. , K. Kim , D. Neff , et al. 2016. “Heat Therapy Promotes the Expression of Angiogenic Regulators in Human Skeletal Muscle.” American Journal of Physiology. Regulatory, Integrative and Comparative Physiology 311: R377–R391. 27357800 10.1152/ajpregu.00134.2016 PMC5008657

[cph470089-bib-0445] Kunbootsri, N. 2013. “The Effect of Six‐Weeks of Sauna on Treatment Autonomic Nervous System, Peak Nasal Inspiratory Flow and Lung Functions of Allergic Rhinitis Thai Patients.” Asian Pacific Journal of Allergy and Immunology 31, no. 2. 10.12932/ap0262.31.2.2013. 23859414

[cph470089-bib-0234] Kunutsor, S. K. , T. Laukkanen , and J. A. Laukkanen . 2018. “Longitudinal Associations of Sauna Bathing With Inflammation and Oxidative Stress: The KIHD Prospective Cohort Study.” Annals of Medicine 50: 437–442. 29897261 10.1080/07853890.2018.1489143

[cph470089-bib-0235] Kurucz, I. , A. Morva , A. Vaag , et al. 2002. “Decreased Expression of Heat Shock Protein 72 in Skeletal Muscle of Patients With Type 2 Diabetes Correlates With Insulin Resistance.” Diabetes 51: 1102–1109. 11916932 10.2337/diabetes.51.4.1102

[cph470089-bib-0236] Kuwahata, S. , M. Miyata , S. Fujita , et al. 2011. “Improvement of Autonomic Nervous Activity by Waon Therapy in Patients With Chronic Heart Failure.” Journal of Cardiology 57: 100–106. 20884178 10.1016/j.jjcc.2010.08.005

[cph470089-bib-0237] Kwon, M. , L. Robins , M. L. McGlynn , et al. 2022. “No Mitochondrial Related Transcriptional Changes in Human Skeletal Muscle After Local Heat Application.” International Journal of Environmental Research and Public Health 19:17051. 36554930 10.3390/ijerph192417051 PMC9779680

[cph470089-bib-0238] Labbadia, J. , H. Cunliffe , A. Weiss , et al. 2011. “Altered Chromatin Architecture Underlies Progressive Impairment of the Heat Shock Response in Mouse Models of Huntington Disease.” Journal of Clinical Investigation 121: 3306–3319. 21785217 10.1172/JCI57413 PMC3148745

[cph470089-bib-0239] Labidi, M. , M. Alhammoud , K. Mtibaa , et al. 2024. “The Effects of Heat Therapy During Immobilization and Rehabilitation on Muscle Atrophy and Strength Loss at Return to Sports in Healthy Humans.” Orthopaedic Journal of Sports Medicine 12: 23259671241281727. 39444938 10.1177/23259671241281727 PMC11497528

[cph470089-bib-0240] Lakatta, E. G. , and D. Levy . 2003. “Arterial and Cardiac Aging: Major Shareholders in Cardiovascular Disease Enterprises: Part I: Aging Arteries: A “Set Up” for Vascular Disease.” Circulation 107: 139–146. 12515756 10.1161/01.cir.0000048892.83521.58

[cph470089-bib-0241] Lansdown, A. , and D. A. Rees . 2012. “The Sympathetic Nervous System in Polycystic Ovary Syndrome: A Novel Therapeutic Target?” Clinical Endocrinology 77: 791–801. 22882204 10.1111/cen.12003

[cph470089-bib-0242] Lanza, I. R. , and K. S. Nair . 2009. “Muscle Mitochondrial Changes With Aging and Exercise.” American Journal of Clinical Nutrition 89: 467S–471S. 19056588 10.3945/ajcn.2008.26717D PMC2715293

[cph470089-bib-0243] Laukkanen, J. A. , and S. K. Kunutsor . 2024. “The Multifaceted Benefits of Passive Heat Therapies for Extending the Healthspan: A Comprehensive Review With a Focus on Finnish Sauna.” Temperature 11: 27–51. 10.1080/23328940.2023.2300623 PMC10989710 38577299

[cph470089-bib-0244] Laukkanen, J. A. , and T. Laukkanen . 2018. “Sauna Bathing and Systemic Inflammation.” European Journal of Epidemiology 33: 351–353. 29209938 10.1007/s10654-017-0335-y

[cph470089-bib-0245] Laukkanen, T. , H. Khan , F. Zaccardi , and J. A. Laukkanen . 2015. “Association Between Sauna Bathing and Fatal Cardiovascular and All‐Cause Mortality Events.” JAMA Internal Medicine 175: 542–548. 25705824 10.1001/jamainternmed.2014.8187

[cph470089-bib-0246] Laukkanen, T. , S. Kunutsor , J. Kauhanen , and J. A. Laukkanen . 2017. “Sauna Bathing Is Inversely Associated With Dementia and Alzheimer's Disease in Middle‐Aged Finnish Men.” Age and Ageing 46: 245–249. 27932366 10.1093/ageing/afw212

[cph470089-bib-0247] Laukkanen, T. , S. K. Kunutsor , H. Khan , P. Willeit , F. Zaccardi , and J. A. Laukkanen . 2018. “Sauna Bathing Is Associated With Reduced Cardiovascular Mortality and Improves Risk Prediction in Men and Women: A Prospective Cohort Study.” BMC Medicine 16: 219. 30486813 10.1186/s12916-018-1198-0 PMC6262976

[cph470089-bib-0248] Laukkanen, T. , J. A. Laukkanen , and S. K. Kunutsor . 2018. “Sauna Bathing and Risk of Psychotic Disorders: A Prospective Cohort Study.” Medical Principles and Practice 27: 562–569. 30173212 10.1159/000493392 PMC6422146

[cph470089-bib-0249] Laukkanen, T. , J. Lipponen , S. K. Kunutsor , et al. 2019. “Recovery From Sauna Bathing Favorably Modulates Cardiac Autonomic Nervous System.” Complementary Therapies in Medicine 45: 190–197. 31331560 10.1016/j.ctim.2019.06.011

[cph470089-bib-0250] Leach, O. K. , K. Strong , G. W. Mack , and J. R. Gifford . 2024. “The Vascular Response to Acute Sauna Heating Is Similar in Young and Middle‐Aged Adults.” Journal of Applied Physiology 136: 573–582. 38271083 10.1152/japplphysiol.00287.2023

[cph470089-bib-0251] Lee, E. , I. A. Kolunsarka , J. Kostensalo , et al. 2022. “The Effects of Regular Sauna Bathing in Conjunction With Exercise on Cardiovascular Function: A Multi‐Arm Randomized Controlled Trial.” American Journal of Physiology. Regulatory, Integrative and Comparative Physiology 323: R289–R299. 35785965 10.1152/ajpregu.00076.2022 PMC9394774

[cph470089-bib-0252] Lee, E. , T. Laukkanen , S. K. Kunutsor , et al. 2018. “Sauna Exposure Leads to Improved Arterial Compliance: Findings From a Non‐Randomised Experimental Study.” European Journal of Preventive Cardiology 25: 130–138. 29048215 10.1177/2047487317737629

[cph470089-bib-0253] Lee, H. K. , P. Kumar , Q. Fu , K. M. Rosen , and H. W. Querfurth . 2009. “The Insulin/Akt Signaling Pathway Is Targeted by Intracellular Beta‐Amyloid.” Molecular Biology of the Cell 20: 1533–1544. 19144826 10.1091/mbc.E08-07-0777 PMC2649265

[cph470089-bib-0254] Leibson, C. L. , W. A. Rocca , V. A. Hanson , et al. 1997. “Risk of Dementia Among Persons With Diabetes Mellitus: A Population‐Based Cohort Study.” American Journal of Epidemiology 145: 301–308. 9054233 10.1093/oxfordjournals.aje.a009106

[cph470089-bib-0255] Leicht, C. A. , L. J. James , J. H. B. Briscoe , and S. P. Hoekstra . 2019. “Hot Water Immersion Acutely Increases Postprandial Glucose Concentrations.” Physiological Reports 7: e14223. 31642205 10.14814/phy2.14223 PMC6805849

[cph470089-bib-0256] Leicht, C. A. , K. Kouda , Y. Umemoto , et al. 2015. “Hot Water Immersion Induces an Acute Cytokine Response in Cervical Spinal Cord Injury.” European Journal of Applied Physiology 115: 2243–2252. 26105530 10.1007/s00421-015-3206-9

[cph470089-bib-0257] Leritz, E. C. , D. H. Salat , V. J. Williams , et al. 2011. “Thickness of the Human Cerebral Cortex Is Associated With Metrics of Cerebrovascular Health in a Normative Sample of Community Dwelling Older Adults.” NeuroImage 54: 2659–2671. 21035552 10.1016/j.neuroimage.2010.10.050 PMC3026290

[cph470089-bib-0258] Li, D. K. , T. Janevic , R. Odouli , and L. Liu . 2003. “Hot Tub Use During Pregnancy and the Risk of Miscarriage.” American Journal of Epidemiology 158: 931–937. 14607798 10.1093/aje/kwg243

[cph470089-bib-0259] Liang, M. , X. Wang , Y. Yuan , Q. Zhou , C. Tong , and W. Jiang . 2009. “Different Effect of Glutamine on Macrophage Tumor Necrosis Factor‐Alpha Release and Heat Shock Protein 72 Expression in Vitro and in Vivo.” Acta Biochimica et Biophysica Sinica Shanghai 41: 171–177. 10.1093/abbs/gmn020 19204835

[cph470089-bib-0260] Liao, W.‐C. 2002. “Effects of Passive Bodyheating on Bodytemperature and Sleep Regulation in the Elderly: A Systematic Review.” International Journal of Nursing Studies 39: 803–810. 12379298 10.1016/s0020-7489(02)00023-8

[cph470089-bib-0261] Lille, S. , C.‐Y. Su , T. Schoeller , et al. 1999. “Induction of Heat‐Shock Protein 72 in Rat Skeletal Muscle Does Not Increase Tolerance to Ischemia‐Reperfusion Injury.” Muscle & Nerve 22: 390–393. 10086900 10.1002/(sici)1097-4598(199903)22:3<390::aid-mus12>3.0.co;2-1

[cph470089-bib-0262] Liu, C. T. , and G. A. Brooks . 2012. “Mild Heat Stress Induces Mitochondrial Biogenesis in C2C12 Myotubes.” Journal of Applied Physiology 112: 354–361. 22052865 10.1152/japplphysiol.00989.2011 PMC3774254

[cph470089-bib-0263] Liu, Y. , S. J. Perdomo , J. Ward , et al. 2019. “Vascular Health Is Associated With Amyloid‐beta in Cognitively Normal Older Adults.” Journal of Alzheimer's Disease 70: 467–475. 10.3233/JAD-181268 PMC6700615 31256125

[cph470089-bib-0264] Louie, J. C. , N. Fujii , R. D. Meade , and G. P. Kenny . 2016. “The Roles of the Na+ /K+‐ATPase, NKCC, and K+ Channels in Regulating Local Sweating and Cutaneous Blood Flow During Exercise in Humans in Vivo.” Physiological Reports 4: e13024. 27881572 10.14814/phy2.13024 PMC5358008

[cph470089-bib-0265] Louie, J. C. , N. Fujii , R. D. Meade , B. D. McNeely , and G. P. Kenny . 2017. “The Roles of KCa, KATP, and KV Channels in Regulating Cutaneous Vasodilation and Sweating During Exercise in the Heat.” American Journal of Physiology. Regulatory, Integrative and Comparative Physiology 312: R821–R827. 28254750 10.1152/ajpregu.00507.2016 PMC5451568

[cph470089-bib-0266] Lubrano, E. , P. F. Mazas , J. Freiwald , et al. 2023. “An International Multidisciplinary Delphi‐Based Consensus on Heat Therapy in Musculoskeletal Pain.” Pain and therapy 12: 93–110. 35932408 10.1007/s40122-022-00419-4 PMC9845456

[cph470089-bib-0267] Luchsinger, J. A. , C. Reitz , B. Patel , M. X. Tang , J. J. Manly , and R. Mayeux . 2007. “Relation of Diabetes to Mild Cognitive Impairment.” Archives of Neurology 64: 570–575. 17420320 10.1001/archneur.64.4.570

[cph470089-bib-0268] Macauley, S. L. , M. Stanley , E. E. Caesar , et al. 2015. “Hyperglycemia Modulates Extracellular Amyloid‐beta Concentrations and Neuronal Activity In Vivo.” Journal of Clinical Investigation 125: 2463–2467. 25938784 10.1172/JCI79742 PMC4497756

[cph470089-bib-0269] Maeda, T. , K. Mimori , S. Suzuki , T. Horiuchi , and N. Makino . 2018. “Preventive and Promotive Effects of Habitual Hot Spa‐Bathing on the Elderly in Japan.” Scientific Reports 8: 133. 29317745 10.1038/s41598-017-18488-3 PMC5760572

[cph470089-bib-0270] Maloyan, A. , A. Palmon , and M. Horowitz . 1999. “Heat Acclimation Increases the Basal HSP72 Level and Alters Its Production Dynamics During Heat Stress.” American Journal of Physiology. Regulatory, Integrative and Comparative Physiology 276: R1506–R1515. 10.1152/ajpregu.1999.276.5.R1506 10233045

[cph470089-bib-0271] Mander, B. A. 2013. “Disturbed Sleep in Preclinical Cognitive Impairment: Cause and Effect?” Sleep 36: 1275–1276. 23997358 10.5665/sleep.2942 PMC3738034

[cph470089-bib-0272] Mangum, J. E. , K. W. Needham , D. C. Sieck , et al. 2022. “The Effect of Local Passive Heating on Skeletal Muscle Histamine Concentration: Implications for Exercise‐Induced Histamine Release.” Journal of Applied Physiology 132: 367–374. 34941436 10.1152/japplphysiol.00740.2021 PMC8799384

[cph470089-bib-0273] Marchant, E. D. , J. P. Kaluhiokalani , T. E. Wallace , et al. 2022. “Localized Heat Therapy Improves Mitochondrial Respiratory Capacity but Not Fatty Acid Oxidation.” International Journal of Molecular Sciences 23: 8500. 35955635 10.3390/ijms23158500 PMC9369322

[cph470089-bib-0274] Marger, C. F. , L. K. Hicklin , and D. P. Garner . 2016. “Effects of Bikram Yoga on Body Composition, Blood Pressure, and Sleep Patterns in Adult Practitioners.” Journal of Basic & Applied Sciences 12:75–80.

[cph470089-bib-0275] Mason, A. E. , A. Chowdhary , W. Hartogensis , et al. 2024. “Feasibility and Acceptability of an Integrated Mind‐Body Intervention for Depression: Whole‐Body Hyperthermia (WBH) and Cognitive Behavioral Therapy (CBT).” International Journal of Hyperthermia 41: 2351459. 38743265 10.1080/02656736.2024.2351459 PMC11216717

[cph470089-bib-0276] Masuda, A. , M. Miyata , T. Kihara , S. Minagoe , and C. Tei . 2004. “Repeated Sauna Therapy Reduces Urinary 8‐Epi‐Prostaglandin F2.ALPHA.” Japanese Heart Journal 45: 297–303. 15090706 10.1536/jhj.45.297

[cph470089-bib-0277] Masuda, A. , M. Nakazato , T. Kihara , S. Minagoe , and C. Tei . 2005. “Repeated Thermal Therapy Diminishes Appetite Loss and Subjective Complaints in Mildly Depressed Patients.” Psychosomatic Medicine 67: 643–647. 16046381 10.1097/01.psy.0000171812.67767.8f

[cph470089-bib-0278] McCord, G. R. , J. L. Cracowski , and C. T. Minson . 2006. “Prostanoids Contribute to Cutaneous Active Vasodilation in Humans.” American Journal of Physiology. Regulatory, Integrative and Comparative Physiology 291: R596–R602. 16484440 10.1152/ajpregu.00710.2005

[cph470089-bib-0279] Merz, A. A. , and S. Cheng . 2016. “Sex Differences in Cardiovascular Ageing.” Heart 102: 825–831. 26917537 10.1136/heartjnl-2015-308769 PMC5993677

[cph470089-bib-0280] Merz, K. E. , and D. C. Thurmond . 2020. “Role of Skeletal Muscle in Insulin Resistance and Glucose Uptake.” Comprehensive Physiology 10: 785–809. 32940941 10.1002/cphy.c190029 PMC8074531

[cph470089-bib-0281] Milunsky, A. , M. Ulcickas , K. J. Rothman , W. Willett , S. S. Jick , and H. Jick . 1992. “Maternal Heat Exposure and Neural Tube Defects.” JAMA 268: 882–885. 1640616

[cph470089-bib-0282] Miranda Hurtado, M. , C. Meza Valladares , A. Eblen‐Zajjur , and M. Rodriguez‐Fernandez . 2019. “Acute Cardiovascular Responses to a Session of Bikram Yoga: A Pilot Uncontrolled Trial.” Journal of Alternative and Complementary Medicine 25: 398–405. 30698456 10.1089/acm.2018.0261

[cph470089-bib-0283] Miyata, M. , T. Kihara , T. Kubozono , et al. 2008. “Beneficial Effects of Waon Therapy on Patients With Chronic Heart Failure: Results of a Prospective Multicenter Study.” Journal of Cardiology 52: 79–85. 18922381 10.1016/j.jjcc.2008.07.009

[cph470089-bib-0284] Miyata, M. , and C. Tei . 2010. “Waon Therapy for Cardiovascular Disease: Innovative therapy for the 21st century.” Circulation Journal 74: 617–621. 20154403 10.1253/circj.cj-09-0939

[cph470089-bib-0285] Moll, A. , L. M. Ramirez , M. Ninov , J. Schwarz , H. Urlaub , and M. Zweckstetter . 2022. “Hsp Multichaperone Complex Buffers Pathologically Modified Tau.” Nature Communications 13: 3668. 10.1038/s41467-022-31396-z PMC9237115 35760815

[cph470089-bib-0286] Monroe, J. C. , C. Lin , S. M. Perkins , et al. 2020. “Leg Heat Therapy Improves Perceived Physical Function but Does Not Enhance Walking Capacity or Vascular Function in Patients With Peripheral Artery Disease.” Journal of Applied Physiology 129: 1279–1289. 33002377 10.1152/japplphysiol.00277.2020 PMC7792848

[cph470089-bib-0287] Monroe, J. C. , B. J. Pae , C. Kargl , et al. 2022. “Effects of Home‐Based Leg Heat Therapy on Walking Performance in Patients With Symptomatic Peripheral Artery Disease: A Pilot Randomized Trial.” Journal of Applied Physiology 133: 546–560. 35771219 10.1152/japplphysiol.00143.2022 PMC9448284

[cph470089-bib-0288] Moon, B. , N. Duddy , L. Ragolia , and N. Begum . 2003. “Stimulation of Glycogen Synthesis by Heat Shock in L6 Skeletal‐Muscle Cells: Regulatory Role of Site‐Specific Phosphorylation of Glycogen‐Associated Protein Phosphatase 1.” Biochemical Journal 371: 857–866. 12540292 10.1042/BJ20021644 PMC1223329

[cph470089-bib-0289] Morino, K. , K. F. Petersen , and G. I. Shulman . 2006. “Molecular Mechanisms of Insulin Resistance in Humans and Their Potential Links With Mitochondrial Dysfunction.” Diabetes 55, no. Suppl. 2: S9–S15. 17130651 10.2337/db06-S002 PMC2995546

[cph470089-bib-0290] Morris, J. K. , and J. M. Burns . 2012. “Insulin: An Emerging Treatment for Alzheimer's Disease Dementia?” Current Neurology and Neuroscience Reports 12: 520–527. 22791280 10.1007/s11910-012-0297-0 PMC3540744

[cph470089-bib-0291] Morris, J. K. , R. A. Honea , E. D. Vidoni , R. H. Swerdlow , and J. M. Burns . 2014. “Is Alzheimer's Disease a Systemic Disease?” Biochimica et Biophysica Acta 1842: 1340–1349. 24747741 10.1016/j.bbadis.2014.04.012 PMC4126236

[cph470089-bib-0292] Morris, J. K. , E. D. Vidoni , H. M. Wilkins , et al. 2016. “Impaired Fasting Glucose Is Associated With Increased Regional Cerebral Amyloid.” Neurobiology of Aging 44: 138–142. 27318141 10.1016/j.neurobiolaging.2016.04.017 PMC4913037

[cph470089-bib-0293] Morton, J. P. , D. P. Maclaren , N. T. Cable , et al. 2007. “Elevated Core and Muscle Temperature to Levels Comparable to Exercise Do Not Increase Heat Shock Protein Content of Skeletal Muscle of Physically Active Men.” Acta Physiologica (Oxford, England) 190: 319–327. 17488245 10.1111/j.1748-1716.2007.01711.x

[cph470089-bib-0294] Moyen, N. E. , M. S. Ganio , J. M. Burchfield , et al. 2016. “Effect of Passive Heat Stress on Arterial Stiffness in Smokers Versus Non‐Smokers.” International Journal of Biometeorology 60: 499–506. 26266482 10.1007/s00484-015-1046-2

[cph470089-bib-0295] Nagai, M. , S. Hoshide , J. Ishikawa , K. Shimada , and K. Kario . 2008. “Ambulatory Blood Pressure as an Independent Determinant of Brain Atrophy and Cognitive Function in Elderly Hypertension.” Journal of Hypertension 26: 1636–1641. 18622243 10.1097/HJH.0b013e3283018333

[cph470089-bib-0296] Nasiri, K. , M. Shriniy , N. J. Pashaki , et al. 2024. “The Effect of Foot Bath on Sleep Quality in the Elderly: A Systematic Review.” BMC Geriatrics 24: 191. 38408926 10.1186/s12877-023-04590-x PMC10898139

[cph470089-bib-0297] Nelson, P. T. , I. Alafuzoff , E. H. Bigio , et al. 2012. “Correlation of Alzheimer Disease Neuropathologic Changes With Cognitive Status: A Review of the Literature.” Journal of Neuropathology and Experimental Neurology 71: 362–381. 22487856 10.1097/NEN.0b013e31825018f7 PMC3560290

[cph470089-bib-0298] Ni, H. M. , J. A. Williams , and W. X. Ding . 2015. “Mitochondrial Dynamics and Mitochondrial Quality Control.” Redox Biology 4: 6–13. 25479550 10.1016/j.redox.2014.11.006 PMC4309858

[cph470089-bib-0299] Nikoulina, S. E. , T. P. Ciaraldi , S. Mudaliar , L. Carter , K. Johnson , and R. R. Henry . 2002. “Inhibition of Glycogen Synthase Kinase 3 Improves Insulin Action and Glucose Metabolism in Human Skeletal Muscle.” Diabetes 51: 2190–2198. 12086949 10.2337/diabetes.51.7.2190

[cph470089-bib-0300] Obi, S. , T. Nakajima , T. Hasegawa , et al. 2019. “Heat Induces Myogenic Transcription Factors of Myoblast Cells via Transient Receptor Potential Vanilloid 1 (Trpv1).” FEBS Open Bio 9: 101–113. 10.1002/2211-5463.12550 PMC6325605 30652078

[cph470089-bib-0301] Oehler, R. , E. Pusch , M. Zellner , et al. 2001. “Cell Type‐Specific Variations in the Induction of hsp70 in Human Leukocytes by Feverlike Whole Body Hyperthermia.” Cell Stress & Chaperones 6: 306–315. 11795467 10.1379/1466-1268(2001)006<0306:ctsvit>2.0.co;2 PMC434413

[cph470089-bib-0302] Ohori, T. , T. Nozawa , H. Ihori , et al. 2012. “Effect of Repeated Sauna Treatment on Exercise Tolerance and Endothelial Function in Patients With Chronic Heart Failure.” American Journal of Cardiology 109: 100–104. 21944673 10.1016/j.amjcard.2011.08.014

[cph470089-bib-0444] Oláh, M. , Á. Koncz , J. Fehér , et al. 2011. “The Effect of Balneotherapy on Antioxidant, Inflammatory, and Metabolic Indices in Patients with Cardiovascular Risk Factors (Hypertension and Obesity)—a Randomised, Controlled, Follow‐Up Study.” Contemporary Clinical Trials 32, no. 6: 793–801. 10.1016/j.cct.2011.06.003. 21763463

[cph470089-bib-0304] Ott, A. , R. P. Stolk , F. van Harskamp , H. A. Pols , A. Hofman , and M. M. Breteler . 1999. “Diabetes Mellitus and the Risk of Dementia: The Rotterdam Study.” Neurology 53: 1937–1942. 10599761 10.1212/wnl.53.9.1937

[cph470089-bib-0305] Oyama, J. , Y. Kudo , T. Maeda , K. Node , and N. Makino . 2013. “Hyperthermia by Bathing in a Hot Spring Improves Cardiovascular Functions and Reduces the Production of Inflammatory Cytokines in Patients With Chronic Heart Failure.” Heart and Vessels 28: 173–178. 22231540 10.1007/s00380-011-0220-7

[cph470089-bib-0306] Pal, D. , S. Mukherjee , I. H. Song , and S. B. Nimse . 2021. “GSK‐3 Inhibitors: A New Class of Drugs for Alzheimer's Disease Treatment.” Current Drug Targets 22: 1725–1737. 33459229 10.2174/1389450122666210114095307

[cph470089-bib-0307] Pallubinsky, H. , E. Phielix , B. Dautzenberg , et al. 2020. “Passive Exposure to Heat Improves Glucose Metabolism in Overweight Humans.” Acta Physiologica (Oxford, England) 229: e13488. 32359193 10.1111/apha.13488 PMC7379279

[cph470089-bib-0308] Parker, W. D., Jr. , C. M. Filley , and J. K. Parks . 1990. “Cytochrome Oxidase Deficiency in Alzheimer's Disease.” Neurology 40: 1302–1303. 2166249 10.1212/wnl.40.8.1302

[cph470089-bib-0309] Parsell, D. A. , and S. Lindquist . 1993. “The Function of Heat‐Shock Proteins in Stress Tolerance: Degradation and Reactivation of Damaged Proteins.” Annual Review of Genetics 27: 437–496. 10.1146/annurev.ge.27.120193.002253 8122909

[cph470089-bib-0310] Patti, M. E. , A. J. Butte , S. Crunkhorn , et al. 2003. “Coordinated Reduction of Genes of Oxidative Metabolism in Humans With Insulin Resistance and Diabetes: Potential Role of PGC1 and NRF1.” Proceedings of the National Academy of Sciences of the United States of America 100: 8466–8471. 12832613 10.1073/pnas.1032913100 PMC166252

[cph470089-bib-0311] Pearson, J. , D. A. Low , E. Stohr , et al. 2011. “Hemodynamic Responses to Heat Stress in the Resting and Exercising Human Leg: Insight Into the Effect of Temperature on Skeletal Muscle Blood Flow.” American Journal of Physiology. Regulatory, Integrative and Comparative Physiology 300: R663–R673. 21178127 10.1152/ajpregu.00662.2010 PMC3064274

[cph470089-bib-0312] Pearson, T. A. , G. A. Mensah , R. W. Alexander , et al. 2003. “Markers of Inflammation and Cardiovascular Disease: Application to Clinical and Public Health Practice: A Statement for Healthcare Professionals From the Centers for Disease Control and Prevention and the American Heart Association.” Circulation 107: 499–511. 12551878 10.1161/01.cir.0000052939.59093.45

[cph470089-bib-0313] Peila, R. , B. L. Rodriguez , L. J. Launer , and Honolulu‐Asia Aging S . 2002. “Type 2 Diabetes, APOE Gene, and the Risk for Dementia and Related Pathologies: The Honolulu‐Asia Aging Study.” Diabetes 51: 1256–1262. 11916953 10.2337/diabetes.51.4.1256

[cph470089-bib-0314] Petersen, K. F. , S. Dufour , D. Befroy , R. Garcia , and G. I. Shulman . 2004. “Impaired Mitochondrial Activity in the Insulin‐Resistant Offspring of Patients With Type 2 Diabetes.” New England Journal of Medicine 350: 664–671. 14960743 10.1056/NEJMoa031314 PMC2995502

[cph470089-bib-0315] Petrofsky, J. , L. Berk , G. Bains , I. A. Khowailed , H. Lee , and M. Laymon . 2017. “The Efficacy of Sustained Heat Treatment on Delayed‐Onset Muscle Soreness.” Clinical Journal of Sport Medicine 27: 329–337. 27454218 10.1097/JSM.0000000000000375

[cph470089-bib-0316] Petrovitch, H. , L. R. White , G. Izmirilian , et al. 2000. “Midlife Blood Pressure and Neuritic Plaques, Neurofibrillary Tangles, and Brain Weight at Death: The HAAS. Honolulu‐Asia Aging Study.” Neurobiology of Aging 21: 57–62. 10794849 10.1016/s0197-4580(00)00106-8

[cph470089-bib-0317] Phimphasone‐Brady, P. , K. V. Ross , A. Z. Zhang , M. Sehrt , K. M. McKenney , and L. G. Lebin . 2024. “Mental Health Across the Menstrual Cycle in Polycystic Ovary Syndrome: Insights and Implications.” Current Psychiatry Reports 26: 553–562. 39214948 10.1007/s11920-024-01529-w PMC12005374

[cph470089-bib-0318] Pizzey, F. K. , E. C. Smith , S. L. Ruediger , et al. 2021. “The Effect of Heat Therapy on Blood Pressure and Peripheral Vascular Function: A Systematic Review and meta‐Analysis.” Experimental Physiology 106: 1317–1334. 33866630 10.1113/EP089424

[cph470089-bib-0319] Pradhan, A. D. , J. E. Manson , N. Rifai , J. E. Buring , and P. M. Ridker . 2001. “C‐Reactive Protein, Interleukin 6, and Risk of Developing Type 2 Diabetes Mellitus.” JAMA 286: 327–334. 11466099 10.1001/jama.286.3.327

[cph470089-bib-0320] Pritchard, J. K. A. , A. W. Ackerman , E. R. Gross , et al. 2001. “Heat Shock Protein 90 Mediates the Balance of Nitric Oxide and Superoxide Anion From Endothelial Nitric‐Oxide Synthase *.” Journal of Biological Chemistry 276: 17621–17624. 11278264 10.1074/jbc.C100084200

[cph470089-bib-0321] Profenno, L. A. , A. P. Porsteinsson , and S. V. Faraone . 2010. “Meta‐Analysis of Alzheimer's Disease Risk With Obesity, Diabetes, and Related Disorders.” Biological Psychiatry 67: 505–512. 19358976 10.1016/j.biopsych.2009.02.013

[cph470089-bib-0322] Putkonen, P. T. S. , and E. Eloma . 1976. “Sauna and Physiological Sleep: Increased Slow‐Wave Sleep After Heat Exposure.” In Sauna Studies: Papers Read at the VI International Sauna Congress in Helsinki on August 15–17, 1974, edited by H. Teir , Y. Collan , and P. Valtakari , 270–279. Finnish Sauna Society.

[cph470089-bib-0323] Pyke, K. E. , E. M. Dwyer , and M. E. Tschakovsky . 2004. “Impact of Controlling Shear Rate on Flow‐Mediated Dilation Responses in the Brachial Artery of Humans.” Journal of Applied Physiology 97: 499–508. 15064302 10.1152/japplphysiol.01245.2003

[cph470089-bib-0324] Qin, B. , S.‐J. Fu , X.‐f. Xu , et al. 2024. “Far‐Infrared Radiation and Its Therapeutic Parameters: A Superior Alternative for Future Regenerative Medicine?” Pharmacological Research 208: 107349. 39151679 10.1016/j.phrs.2024.107349

[cph470089-bib-0325] Qiu, Y. , Y. Zhu , W. Jia , S. Chen , and Q. Meng . 2014. “Spa Adjuvant Therapy Improves Diabetic Lower Extremity Arterial Disease.” Complementary Therapies in Medicine 22: 655–661. 25146070 10.1016/j.ctim.2014.05.003

[cph470089-bib-0326] Racinais, S. , M. G. Wilson , and J. D. Périard . 2017. “Passive Heat Acclimation Improves Skeletal Muscle Contractility in Humans.” American Journal of Physiology. Regulatory, Integrative and Comparative Physiology 312: R101–R107. 27903515 10.1152/ajpregu.00431.2016

[cph470089-bib-0327] Rahman, S. M. , J. M. Schroeder‐Gloeckler , R. C. Janssen , et al. 2007. “CCAAT/Enhancing Binding Protein beta Deletion in Mice Attenuates Inflammation, Endoplasmic Reticulum Stress, and Lipid Accumulation in Diet‐Induced Nonalcoholic Steatohepatitis.” Hepatology 45: 1108–1117. 17464987 10.1002/hep.21614

[cph470089-bib-0328] Reed, E. L. , C. C. Uzoekwe , J. K. Atencio , C. T. Minson , and J. R. Halliwill 2025. “Muscle Temperature Increases During a Single Far Infrared Sauna Session Without Changes in Intestinal Temperature.” Journal of Applied Physiology 138: 1628–1637. 40331900 10.1152/japplphysiol.00067.2025 PMC13104632

[cph470089-bib-0446] Roxburgh, B. H. , H. A. Campbell , J. D. Cotter , et al. 2023. “Acute and Adaptive Cardiovascular and Metabolic Effects of Passive Heat Therapy or High‐Intensity Interval Training in Patients with Severe Lower‐Limb Osteoarthritis.” Physiological Reports 11, no. 11. 10.14814/phy2.15699 PMC10257080 37300374

[cph470089-bib-0329] Rhea, E. M. , and W. A. Banks . 2019. “Role of the Blood‐Brain Barrier in Central Nervous System Insulin Resistance.” Frontiers in Neuroscience 13: 521. 31213970 10.3389/fnins.2019.00521 PMC6558081

[cph470089-bib-0330] Richey, R. , H. Hemingway , A. Moore , et al. 2024. “Effect of Home‐Based Heat Therapy on 24‐h Blood Pressure and Cutaneous Microvascular Function in Post‐Menopausal Women With Hypertension.” Physiology 39: 941.

[cph470089-bib-0331] Richey, R. , Y. Ruiz‐Pick , H. Hemingway , et al. 2025. “Home‐Based Heat Therapy Reduces Plasma pTau‐181 in Post‐Menopausal Women With Hypertension.” Physiology 40: 1172.

[cph470089-bib-0332] Richey, R. E. , H. W. Hemingway , A. M. Moore , A. H. Olivencia‐Yurvati , and S. A. Romero . 2022. “Acute Heat Exposure Improves Microvascular Function in Skeletal Muscle of Aged Adults.” American Journal of Physiology. Heart and Circulatory Physiology 322: H386–H393. 35060753 10.1152/ajpheart.00645.2021 PMC8858667

[cph470089-bib-0333] Richey, R. E. , Y. I. Ruiz , H. L. Cope , et al. 2024. “Cyclooxygenase Inhibition Does Not Blunt Thermal Hyperemia in Skeletal Muscle of Humans.” Journal of Applied Physiology 136: 151–157. 38059292 10.1152/japplphysiol.00657.2023 PMC11191756

[cph470089-bib-0334] Richter, E. A. , and M. Hargreaves . 2013. “Exercise, GLUT4, and Skeletal Muscle Glucose Uptake.” Physiological Reviews 93: 993–1017. 23899560 10.1152/physrev.00038.2012

[cph470089-bib-0335] Rippe, J. M. 2019. “Lifestyle Strategies for Risk Factor Reduction, Prevention, and Treatment of Cardiovascular Disease.” American Journal of Lifestyle Medicine 13: 204–212. 30800027 10.1177/1559827618812395 PMC6378495

[cph470089-bib-0336] Rissanen, J. A. , K. Häkkinen , J. A. Laukkanen , and A. Häkkinen . 2020. “Acute Hemodynamic Responses to Combined Exercise and Sauna.” International Journal of Sports Medicine 41: 824–831. 32599642 10.1055/a-1186-1716

[cph470089-bib-0337] Ritossa, F. 1962. “A New Puffing Pattern Induced by Temperature Shock and DNP in drosophila.” Experientia 18: 571–573.

[cph470089-bib-0338] Rodrigues, P. , G. M. Minett , and L. B. R. Orssatto . 2025. “Turning Up the Heat on Skeletal Muscle Adaptations and Neuromuscular Function: Key Considerations for Passive Heating Prescription and Best Practices.” European Journal of Applied Physiology 125: 2741–2750. 40728571 10.1007/s00421-025-05917-9 PMC12479662

[cph470089-bib-0339] Rodrigues, P. , L. B. R. Orssatto , D. Gagnon , et al. 2024. “Passive Heat Therapy: A Promising Preventive Measure for People at Risk of Adverse Health Outcomes During Heat Extremes.” Journal of Applied Physiology 136: 677–694. 38299219 10.1152/japplphysiol.00701.2023

[cph470089-bib-0340] Rodrigues, P. , L. B. R. Orssatto , G. S. Trajano , L. Wharton , and G. M. Minett . 2023. “Increases in Muscle Temperature by Hot Water Improve Muscle Contractile Function and Reduce Motor Unit Discharge Rates.” Scandinavian Journal of Medicine & Science in Sports 33: 754–765. 36610040 10.1111/sms.14312

[cph470089-bib-0341] Rodrigues, P. , G. S. Trajano , L. Wharton , L. B. R. Orssatto , and G. M. Minett . 2021. “A Passive Increase in Muscle Temperature Enhances Rapid Force Production and Neuromuscular Function in Healthy Adults.” Journal of Science and Medicine in Sport 24: 818–823. 33487572 10.1016/j.jsams.2021.01.003

[cph470089-bib-0342] Rodrigues‐Krause, J. , M. Krause , C. O'Hagan , et al. 2012. “Divergence of Intracellular and Extracellular HSP72 in Type 2 Diabetes: Does Fat Matter?” Cell Stress & Chaperones 17: 293–302. 22215518 10.1007/s12192-011-0319-x PMC3312959

[cph470089-bib-0343] Romero, S. A. , D. Gagnon , A. N. Adams , M. N. Cramer , K. Kouda , and C. G. Crandall . 2017. “Acute Limb Heating Improves Macro‐ and Microvascular Dilator Function in the Leg of Aged Humans.” American Journal of Physiology. Heart and Circulatory Physiology 312: H89–H97. 27836894 10.1152/ajpheart.00519.2016 PMC5283915

[cph470089-bib-0344] Romero, S. A. , R. E. Richey , and H. W. Hemingway . 2022. “Cardiovascular Adjustments After Acute Heat Exposure.” Exercise and Sport Sciences Reviews 50: 194–202. 36044739 10.1249/JES.0000000000000304 PMC9474635

[cph470089-bib-0345] Ruiz‐Pick, Y. I. , H. L. Cope , R. E. Richey , et al. 2025. “Home‐Based Heat Therapy Lowers Blood Pressure and Improves Endothelial Function in Older Adults.” Journal of Applied Physiology 138: 979–987. 40062687 10.1152/japplphysiol.00977.2024 PMC12053574

[cph470089-bib-0346] Saganek, L. J. , D. P. Ignasiak , B. L. Batley , R. E. Potoczak , G. Dodd , and K. P. Gallagher . 1997. “Heat Stress Increases Cardiac HSP72i but Fails to Reduce Myocardial Infarct Size in Rabbits 24 Hours Later.” Basic Research in Cardiology 92: 331–338. 9486354 10.1007/BF00788945

[cph470089-bib-0347] Saltin, B. , A. P. Gagge , and J. A. Stolwijk . 1968. “Muscle Temperature During Submaximal Exercise in Man.” Journal of Applied Physiology 25: 679–688. 5727193 10.1152/jappl.1968.25.6.679

[cph470089-bib-0348] Schapira, A. H. , C. W. Olanow , J. T. Greenamyre , and E. Bezard . 2014. “Slowing of Neurodegeneration in Parkinson's Disease and Huntington's Disease: Future Therapeutic Perspectives.” Lancet 384: 545–555. 24954676 10.1016/S0140-6736(14)61010-2

[cph470089-bib-0349] Schlader, Z. J. , Y. Okada , S. A. Best , Q. Fu , and C. G. Crandall . 2019. “Arterial Stiffness During Whole‐Body Passive Heat Stress in Healthy Older Adults.” Physiological Reports 7: e14094. 31062476 10.14814/phy2.14094 PMC6503298

[cph470089-bib-0350] Schneider, J. L. , Y. Suh , and A. M. Cuervo . 2014. “Deficient Chaperone‐Mediated Autophagy in Liver Leads to Metabolic Dysregulation.” Cell Metabolism 20: 417–432. 25043815 10.1016/j.cmet.2014.06.009 PMC4156578

[cph470089-bib-0351] Seals, D. R. 2014. “Edward F. Adolph Distinguished Lecture: The Remarkable Anti‐Aging Effects of Aerobic Exercise on Systemic Arteries.” Journal of Applied Physiology (Bethesda, MD: 1985) 117: 425–439. 24855137 10.1152/japplphysiol.00362.2014 PMC4157159

[cph470089-bib-0352] Seals, D. R. , V. E. Brunt , and M. J. Rossman . 2018. “Keynote Lecture: Strategies for Optimal Cardiovascular Aging.” American Journal of Physiology. Heart and Circulatory Physiology 315: H183–h188. 29652545 10.1152/ajpheart.00734.2017 PMC6139621

[cph470089-bib-0353] Seals, D. R. , J. N. Justice , and T. J. Larocca . 2016. “Physiological Geroscience: Targeting Function to Increase Healthspan and Achieve Optimal Longevity.” Journal of Physiology 594: 2001–2024. 25639909 10.1113/jphysiol.2014.282665 PMC4933122

[cph470089-bib-0354] Seals, D. R. , K. L. Moreau , P. E. Gates , and I. J. E. Eskurza . 2006. “Modulatory Influences on Ageing of the Vasculature in Healthy Humans.” 41: 501–507. 10.1016/j.exger.2006.01.001 16537099

[cph470089-bib-0443] Sebők, J. , Z. Édel , F. Dembrovszky , et al. 2022. “Effect of HEAT Therapy in patiEnts with Type 2 Diabetes Mellitus (HEATED): Protocol for a Randomised Controlled Trial.” BMJ Open 12, no. 7: e062122. 10.1136/bmjopen-2022-062122. PMC9277369 35820741

[cph470089-bib-0355] Sebők, J. , Z. Édel , S. Váncsa , et al. 2021. “Heat Therapy Shows Benefit in Patients With Type 2 Diabetes Mellitus: A Systematic Review and meta‐Analysis.” International Journal of Hyperthermia 38: 1650–1659. 34808071 10.1080/02656736.2021.2003445

[cph470089-bib-0356] Sekins, K. M. , D. Dundore , A. F. Emery , J. F. Lehmann , P. W. McGrath , and W. B. Nelp . 1980. “Muscle Blood Flow Changes in Response to 915 MHz Diathermy With Surface Cooling as Measured by Xe133 Clearance.” Archives of Physical Medicine and Rehabilitation 61: 105–113. 6989343

[cph470089-bib-0357] Sekins, K. M. , J. F. Lehmann , P. Esselman , et al. 1984. “Local Muscle Blood Flow and Temperature Responses to 915MHz Diathermy as Simultaneously Measured and Numerically Predicted.” Archives of Physical Medicine and Rehabilitation 65: 1–7. 6691788

[cph470089-bib-0358] Selkow, N. M. , C. Day , Z. Liu , J. M. Hart , J. Hertel , and S. A. Saliba . 2012. “Microvascular Perfusion and Intramuscular Temperature of the Calf During Cooling.” Medicine and Science in Sports and Exercise 44: 850–856. 21988932 10.1249/MSS.0b013e31823bced9 PMC3295862

[cph470089-bib-0359] Serbulea, M. , and U. Payyappallimana . 2012. “Onsen (Hot Springs) in Japan—Transforming Terrain Into Healing Landscapes.” Health & Place 18: 1366–1373. 22878276 10.1016/j.healthplace.2012.06.020

[cph470089-bib-0360] Shah, V. , R. Wiest , G. Garcia‐Cardena , G. Cadelina , R. J. Groszmann , and W. C. Sessa . 1999. “Hsp90 Regulation of Endothelial Nitric Oxide Synthase Contributes to Vascular Control in Portal Hypertension.” American Journal of Physiology. Gastrointestinal and Liver Physiology 277: G463–G468. 10.1152/ajpgi.1999.277.2.G463 10444461

[cph470089-bib-0361] Shamaki, G. R. , F. Markson , D. Soji‐Ayoade , C. C. Agwuegbo , M. O. Bamgbose , and B. M. Tamunoinemi . 2022. “Peripheral Artery Disease: A Comprehensive Updated Review.” Current Problems in Cardiology 47: 101082. 34906615 10.1016/j.cpcardiol.2021.101082

[cph470089-bib-0362] Shefi, S. , P. E. Tarapore , T. J. Walsh , M. Croughan , and P. J. Turek . 2007. “Wet Heat Exposure: A Potentially Reversible Cause of Low Semen Quality in Infertile Men.” International Braz J Urol 33: 50–57. 17335598 10.1590/s1677-55382007000100008

[cph470089-bib-0363] Sherman, M. Y. , and A. L. Goldberg . 2001. “Cellular Defenses Against Unfolded Proteins: A Cell Biologist Thinks About Neurodegenerative Diseases.” Neuron 29: 15–32. 11182078 10.1016/s0896-6273(01)00177-5

[cph470089-bib-0364] Shevtsov, M. A. , A. V. Pozdnyakov , A. L. Mikhrina , et al. 2014. “Effective Immunotherapy of Rat Glioblastoma With Prolonged Intratumoral Delivery of Exogenous Heat Shock Protein Hsp70.” International Journal of Cancer 135: 2118–2128. 24691976 10.1002/ijc.28858

[cph470089-bib-0365] Shinsato, T. , M. Miyata , T. Kubozono , et al. 2010. “Waon Therapy Mobilizes CD34+ Cells and Improves Peripheral Arterial Disease.” Journal of Cardiology 56: 361–366. 20843662 10.1016/j.jjcc.2010.08.004

[cph470089-bib-0366] Silva, D. F. , J. E. Selfridge , J. Lu , et al. 2013. “Bioenergetic Flux, Mitochondrial Mass and Mitochondrial Morphology Dynamics in AD and MCI Cybrid Cell Lines.” Human Molecular Genetics 22: 3931–3946. 23740939 10.1093/hmg/ddt247 PMC3888119

[cph470089-bib-0367] Sisante, J. V. , E. D. Vidoni , K. Kirkendoll , et al. 2019. “Blunted Cerebrovascular Response Is Associated With Elevated beta‐Amyloid.” Journal of Cerebral Blood Flow and Metabolism 39: 89–96. 28914134 10.1177/0271678X17732449 PMC6311677

[cph470089-bib-0368] Sobajima, M. , T. Nozawa , Y. Fukui , et al. 2015. “Waon Therapy Improves Quality of Life as Well as Cardiac Function and Exercise Capacity in Patients With Chronic Heart Failure.” International Heart Journal 56: 203–208. 25740582 10.1536/ihj.14-266

[cph470089-bib-0369] Soejima, Y. , T. Munemoto , A. Masuda , Y. Uwatoko , M. Miyata , and C. Tei . 2015. “Effects of Waon Therapy on Chronic Fatigue Syndrome: A Pilot Study.” Internal Medicine 54: 333–338. 25748743 10.2169/internalmedicine.54.3042

[cph470089-bib-0370] Somani, Y. B. , J. A. Pawelczyk , M. J. De Souza , P. M. Kris‐Etherton , and D. N. Proctor . 2019. “Aging Women and Their Endothelium: Probing the Relative Role of Estrogen on Vasodilator Function.” American Journal of Physiology. Heart and Circulatory Physiology 317: H395–h404. 31173499 10.1152/ajpheart.00430.2018 PMC6732482

[cph470089-bib-0371] Song, Y. , J. L. Zweier , and Y. Xia . 2001. “Heat‐Shock Protein 90 Augments Neuronal Nitric Oxide Synthase Activity by Enhancing Ca2+/Calmodulin Binding.” Biochemical Journal 355: 357–360. 11284722 10.1042/0264-6021:3550357 PMC1221746

[cph470089-bib-0372] Sreedhar, A. S. , B. V. V. Pardhasaradhi , A. Khar , and U. K. Srinivas . 2002. “A Cross Talk Between Cellular Signalling and Cellular Redox State During Heat‐Induced Apoptosis in a Rat Histiocytoma.” Free Radical Biology and Medicine 32: 221–227. 11827747 10.1016/s0891-5849(01)00796-1

[cph470089-bib-0373] Steensberg, A. , C. P. Fischer , C. Keller , K. Møller , and B. K. Pedersen . 2003. “IL‐6 Enhances Plasma IL‐1ra, IL‐10, and Cortisol in Humans.” American Journal of Physiology. Endocrinology and Metabolism 285: E433–E437. 12857678 10.1152/ajpendo.00074.2003

[cph470089-bib-0374] Stener‐Victorin, E. , E. Jedel , P. O. Janson , and Y. B. Sverrisdottir . 2009. “Low‐Frequency Electroacupuncture and Physical Exercise Decrease High Muscle Sympathetic Nerve Activity in Polycystic Ovary Syndrome.” American Journal of Physiology. Regulatory, Integrative and Comparative Physiology 297: R387–R395. 19494176 10.1152/ajpregu.00197.2009

[cph470089-bib-0375] Stewart, R. , and D. Liolitsa . 1999. “Type 2 diabetes mellitus, cognitive impairment and dementia.” Diabetic Medicine 16: 93–112. 10229302 10.1046/j.1464-5491.1999.00027.x

[cph470089-bib-0376] Su, Y. , and E. R. Block . 2000. “Role of Calpain in Hypoxic Inhibition of Nitric Oxide Synthase Activity in Pulmonary Endothelial Cells.” American Journal of Physiology. Lung Cellular and Molecular Physiology 278: L1204–L1212. 10835326 10.1152/ajplung.2000.278.6.L1204

[cph470089-bib-0377] Sun, Y. , J. R. Zhang , and S. Chen . 2017. “Suppression of Alzheimer's Disease‐Related Phenotypes by the Heat Shock Protein 70 Inducer, Geranylgeranylacetone, in APP/PS1 Transgenic Mice via the ERK/p38 MAPK Signaling Pathway.” Experimental and Therapeutic Medicine 14: 5267–5274. 29285052 10.3892/etm.2017.5253 PMC5740803

[cph470089-bib-0378] Suzuki, K. , B. Murtuza , I. A. Sammut , et al. 2002. “Heat Shock Protein 72 Enhances Manganese Superoxide Dismutase Activity During Myocardial Ischemia‐Reperfusion Injury, Associated With Mitochondrial Protection and Apoptosis Reduction.” Circulation 106: I‐270. 12354745

[cph470089-bib-0379] Sweeney, M. D. , K. Kisler , A. Montagne , A. W. Toga , and B. V. Zlokovic . 2018. “The Role of Brain Vasculature in Neurodegenerative Disorders.” Nature Neuroscience 21: 1318–1331. 30250261 10.1038/s41593-018-0234-x PMC6198802

[cph470089-bib-0380] Swerdlow, R. H. 2018. “Mitochondria and Mitochondrial Cascades in Alzheimer's Disease.” Journal of Alzheimer's Disease 62: 1403–1416. 10.3233/JAD-170585 PMC5869994 29036828

[cph470089-bib-0381] Taddei, S. , A. Virdis , P. Mattei , et al. 1995. “Aging and Endothelial Function in Normotensive Subjects and Patients With Essential Hypertension.” Circulation 91: 1981–1987. 7895356 10.1161/01.cir.91.7.1981

[cph470089-bib-0382] Tanaka, H. , and D. R. Seals . 2008. “Endurance Exercise Performance in Masters Athletes: Age‐Associated Changes and Underlying Physiological Mechanisms.” Journal of Physiology 586: 55–63. 17717011 10.1113/jphysiol.2007.141879 PMC2375571

[cph470089-bib-0383] Taylor, M. K. , D. K. Sullivan , R. H. Swerdlow , et al. 2017. “A High‐Glycemic Diet Is Associated With Cerebral Amyloid Burden in Cognitively Normal Older Adults.” American Journal of Clinical Nutrition 106: 1463–1470. 29070566 10.3945/ajcn.117.162263 PMC5698843

[cph470089-bib-0384] Tei, C. , T. Shinsato , M. Miyata , T. Kihara , and S. Hamasaki . 2007. “Waon Therapy Improves Peripheral Arterial Disease.” Journal of the American College of Cardiology 50: 2169–2171. 18036456 10.1016/j.jacc.2007.08.025

[cph470089-bib-0385] ter Meulen, W. G. , S. Draisma , A. M. van Hemert , et al. 2021. “Depressive and Anxiety Disorders in Concert–A Synthesis of Findings on Comorbidity in the NESDA Study.” Journal of Affective Disorders 284: 85–97. 33588240 10.1016/j.jad.2021.02.004

[cph470089-bib-0386] Teri, L. , S. Borson , H. A. Kiyak , and M. Yamagishi . 1989. “Behavioral Disturbance, Cognitive Dysfunction, and Functional Skill. Prevalence and Relationship in Alzheimer's Disease.” Journal of the American Geriatrics Society 37: 109–116. 2783433 10.1111/j.1532-5415.1989.tb05868.x

[cph470089-bib-0387] Thomas, K. N. , A. M. van Rij , S. J. Lucas , and J. D. Cotter . 2017. “Lower‐Limb Hot‐Water Immersion Acutely Induces Beneficial Hemodynamic and Cardiovascular Responses in Peripheral Arterial Disease and Healthy, Elderly Controls.” American Journal of Physiology. Regulatory, Integrative and Comparative Physiology 312: R281–r291. 28003211 10.1152/ajpregu.00404.2016

[cph470089-bib-0388] Thomas, K. N. , A. M. van Rij , S. J. Lucas , A. R. Gray , and J. D. Cotter . 2016. “Substantive Hemodynamic and Thermal Strain Upon Completing Lower‐Limb Hot‐Water Immersion; Comparisons With Treadmill Running.” Temperature (Austin) 3: 286–297. 27857958 10.1080/23328940.2016.1156215 PMC4964998

[cph470089-bib-0389] Thomson, D. M. 2018. “The Role of AMPK in the Regulation of Skeletal Muscle Size, Hypertrophy, and Regeneration.” International Journal of Molecular Sciences 19: 3125. 30314396 10.3390/ijms19103125 PMC6212977

[cph470089-bib-0390] Tissiéres, A. , H. K. Mitchell , and U. M. Tracy . 1974. “Protein Synthesis in Salivary Glands of *Drosophila melanogaster* : Relation to Chromosome Puffs.” Journal of Molecular Biology 84: 389–398. 4219221 10.1016/0022-2836(74)90447-1

[cph470089-bib-0391] Tracy, B. L. , and C. E. F. Hart . 2013. “Bikram Yoga Training and Physical Fitness in Healthy Young Adults.” Journal of Strength & Conditioning Research 27: 822–830. 22592178 10.1519/JSC.0b013e31825c340f

[cph470089-bib-0392] Tracy, B. L. , C. E. F. Hart , B. L. Tracy , and C. E. F. Hart . 2013. “Bikram Yoga Training and Physical Fitness in Healthy Young Adults.” Journal of Strength and Conditioning Research 27: 822–830. 22592178 10.1519/JSC.0b013e31825c340f

[cph470089-bib-0393] Tranah, G. J. , T. Blackwell , K. L. Stone , et al. 2011. “Circadian Activity Rhythms and Risk of Incident Dementia and Mild Cognitive Impairment in Older Women.” Annals of Neurology 70: 722–732. 22162057 10.1002/ana.22468 PMC3244839

[cph470089-bib-0394] Tsao, C. W. , A. W. Aday , Z. I. Almarzooq , et al. 2023. “Heart Disease and Stroke Statistics—2023 Update: A Report From the American Heart Association.” Circulation 147: e93–e621. 36695182 10.1161/CIR.0000000000001123 PMC12135016

[cph470089-bib-0395] Vaile, J. , S. Halson , N. Gill , and B. Dawson . 2008. “Effect of Hydrotherapy on the Signs and Symptoms of Delayed Onset Muscle Soreness.” European Journal of Applied Physiology 102: 447–455. 17978833 10.1007/s00421-007-0605-6

[cph470089-bib-0396] van der Heide, L. P. , G. M. Ramakers , and M. P. Smidt . 2006. “Insulin Signaling in the Central Nervous System: Learning to Survive.” Progress in Neurobiology 79: 205–221. 16916571 10.1016/j.pneurobio.2006.06.003

[cph470089-bib-0397] Vatansever, F. , and M. R. Hamblin . 2012. “Far Infrared Radiation (FIR): Its Biological Effects and Medical Applications.” Photonics & Lasers in Medicine 1: 255–266. 10.1515/plm-2012-0034 PMC3699878 23833705

[cph470089-bib-0398] Verhagen, A. P. , S. M. Bierma‐Zeinstra , M. Boers , et al. 2015. “Balneotherapy (or Spa Therapy) for Rheumatoid Arthritis.” Cochrane Database of Systematic Reviews 2015: CD000518. 25862243 10.1002/14651858.CD000518.pub2 PMC7045434

[cph470089-bib-0399] Von Schulze, A. T. , F. Deng , K. N. Z. Fuller , et al. 2021. “Heat Treatment Improves Hepatic Mitochondrial Respiratory Efficiency via Mitochondrial Remodeling.” Function 2: zqab001. 33629069 10.1093/function/zqab001 PMC7886620

[cph470089-bib-0400] Vozarova, B. , C. Weyer , K. Hanson , P. A. Tataranni , C. Bogardus , and R. E. Pratley . 2001. “Circulating Interleukin‐6 in Relation to Adiposity, Insulin Action, and Insulin Secretion.” Obesity Research 9: 414–417. 11445664 10.1038/oby.2001.54

[cph470089-bib-0401] Walker, E. R. , R. E. McGee , and B. G. Druss . 2015. “Mortality in Mental Disorders and Global Disease Burden Implications: A Systematic Review and meta‐Analysis.” JAMA Psychiatry 72: 334–341. 25671328 10.1001/jamapsychiatry.2014.2502 PMC4461039

[cph470089-bib-0402] Wang, Y. , H. Lu , S. Li , et al. 2022. “Effect of Cold and Heat Therapies on Pain Relief in Patients With Delayed Onset Muscle Soreness: A Network meta‐Analysis.” Journal of Rehabilitation Medicine 54: jrm00258. 34636405 10.2340/jrm.v53.331 PMC8862647

[cph470089-bib-0403] Webster, C. P. , E. F. Smith , P. J. Shaw , and K. J. De Vos . 2017. “Protein Homeostasis in Amyotrophic Lateral Sclerosis: Therapeutic Opportunities?” Frontiers in Molecular Neuroscience 10: 123. 28512398 10.3389/fnmol.2017.00123 PMC5411428

[cph470089-bib-0404] Welc, S. S. , N. A. Phillips , J. Oca‐Cossio , S. M. Wallet , D. L. Chen , and T. L. Clanton . 2012. “Hyperthermia Increases Interleukin‐6 in Mouse Skeletal Muscle.” American Journal of Physiology. Cell Physiology 303: C455–C466. 22673618 10.1152/ajpcell.00028.2012 PMC3422986

[cph470089-bib-0405] Whelton, P. K. , R. M. Carey , W. S. Aronow , et al. 2017. “CC/AHA/AAPA/ABC/ACPM/AGS/APhA/ASH/ASPC/NMA/PCNA Guideline for the Prevention, Detection, Evaluation, and Management of High Blood Pressure in Adults: A Report of the American College of Cardiology/American Heart Association Task Force on Clinical Practice Guidelines.” Hypertension 71: e13–e115. 29133356 10.1161/HYP.0000000000000065

[cph470089-bib-0406] Whitham, M. , G. J. Walker , and N. C. Bishop . 2006. “Effect of Caffeine Supplementation on the Extracellular Heat Shock Protein 72 Response to Exercise.” Journal of Applied Physiology 101: 1222–1227. 16794026 10.1152/japplphysiol.00409.2006

[cph470089-bib-0407] Wild, R. A. , E. Carmina , E. Diamanti‐Kandarakis , et al. 2010. “Assessment of Cardiovascular Risk and Prevention of Cardiovascular Disease in Women With the Polycystic Ovary Syndrome: A Consensus Statement by the Androgen Excess and Polycystic Ovary Syndrome (AE‐PCOS) Society.” Journal of Clinical Endocrinology & Metabolism 95: 2038–2049. 20375205 10.1210/jc.2009-2724

[cph470089-bib-0408] Willmund, F. , M. del Alamo , S. Pechmann , et al. 2013. “The Cotranslational Function of Ribosome‐Associated Hsp70 in Eukaryotic Protein Homeostasis.” Cell 152: 196–209. 23332755 10.1016/j.cell.2012.12.001 PMC3553497

[cph470089-bib-0409] Wilson, P. W. F. , M. Pencina , P. Jacques , J. Selhub , R. D'Agostino , and C. J. O'Donnell . 2008. “C‐Reactive Protein and Reclassification of Cardiovascular Risk in the Framingham Heart Study.” Circulation. Cardiovascular Quality and Outcomes 1: 92–97. 20031795 10.1161/CIRCOUTCOMES.108.831198 PMC3033831

[cph470089-bib-0410] Wilson, T. E. , and C. G. Crandall . 2011. “Effect of Thermal Stress on Cardiac Function.” Exercise and Sport Sciences Reviews 39: 12–17. 21088607 10.1097/JES.0b013e318201eed6 PMC3076691

[cph470089-bib-0411] Winklhofer, K. F. , J. Tatzelt , and C. Haass . 2008. “The Two Faces of Protein Misfolding: Gain‐ and Loss‐of‐Function in Neurodegenerative Diseases.” EMBO Journal 27: 336–349. 18216876 10.1038/sj.emboj.7601930 PMC2234348

[cph470089-bib-0412] Wong, B. J. , S. J. Williams , and C. T. Minson . 2006. “Minimal Role for H1 and H2 Histamine Receptors in Cutaneous Thermal Hyperemia to Local Heating in Humans.” Journal of Applied Physiology 100: 535–540. 16195389 10.1152/japplphysiol.00902.2005

[cph470089-bib-0413] Wu, C. 1995. “Heat Shock Transcription Factors: Structure and Regulation.” Annual Review of Cell and Developmental Biology 11: 441–469. 10.1146/annurev.cb.11.110195.002301 8689565

[cph470089-bib-0414] Xi, L. , J. Chelliah , M. A. Nayeem , J. E. Levasseur , M. L. Hess , and R. C. Kukreja . 1998. “Whole Body Heat Shock Fails to Protect Mouse Heart Against Ischemia/Reperfusion Injury: Role of 72 kDa Heat Shock Protein and Antioxidant Enzymes.” Journal of Molecular and Cellular Cardiology 30: 2213–2227. 9925359 10.1006/jmcc.1998.0781

[cph470089-bib-0415] Xu, W. , C. Qiu , M. Gatz , N. L. Pedersen , B. Johansson , and L. Fratiglioni . 2009. “Mid‐ and Late‐Life Diabetes in Relation to the Risk of Dementia: A Population‐Based Twin Study.” Diabetes 58: 71–77. 18952836 10.2337/db08-0586 PMC2606895

[cph470089-bib-0416] Yaffe, K. , T. Blackwell , A. M. Kanaya , N. Davidowitz , E. Barrett‐Connor , and K. Krueger . 2004. “Diabetes, Impaired Fasting Glucose, and Development of Cognitive Impairment in Older Women.” Neurology 63: 658–663. 15326238 10.1212/01.wnl.0000134666.64593.ba

[cph470089-bib-0417] Yamashita, N. , S. Hoshida , M. Nishida , et al. 1997. “Heat Shock‐Induced Manganese Superoxide Dismutase Enhances the Tolerance of Cardiac Myocytes to Hypoxia–Reoxygenation Injury.” Journal of Molecular and Cellular Cardiology 29: 1805–1813. 9236135 10.1006/jmcc.1997.0415

[cph470089-bib-0418] Yanes, L. L. , and J. F. Reckelhoff . 2011. “Postmenopausal Hypertension.” American Journal of Hypertension 24: 740–749. 21509049 10.1038/ajh.2011.71 PMC3820162

[cph470089-bib-0419] Yasin, H. , W. E. Mangano , P. Malhotra , A. Farooq , and H. Mohamed . 2017. “Hot Tub Lung: A Diagnostic Challenge.” Cureus 9: e1617. 29098128 10.7759/cureus.1617 PMC5659329

[cph470089-bib-0420] Ye, W. N. , M. Thipse , M. B. Mahdi , et al. 2020. “Can Heat Therapy Help Patients With Heart Failure?” Artificial Organs 44: 680–692. 32017138 10.1111/aor.13659

[cph470089-bib-0421] Yellon, D. M. , E. Pasini , A. Cargnoni , M. S. Marber , D. S. Latchman , and R. Ferrari . 1992. “The Protective Role of Heat Stress in the Ischaemic and Reperfused Rabbit Myocardium.” Journal of Molecular and Cellular Cardiology 24: 895–907. 1433316 10.1016/0022-2828(92)91102-b

[cph470089-bib-0422] Yildiz, O. 2007. “Vascular Smooth Muscle and Endothelial Functions in Aging.” Annals of the New York Academy of Sciences 1100: 353–360. 17460198 10.1196/annals.1395.038

[cph470089-bib-0423] Ying, H. , C. Jianping , Y. Jianqing , and Z. Shanquan . 2016. “Cognitive Variations Among Vascular Dementia Subtypes Caused by Small‐, Large‐, or Mixed‐Vessel Disease.” Archives of Medical Science 12: 747–753. 27478455 10.5114/aoms.2016.60962 PMC4947622

[cph470089-bib-0424] Ylikahri, R. , E. Heikkonen , and A. Soukas . 1988. “The Sauna and Alcohol.” Annals of Clinical Research 20: 287–291. 3218903

[cph470089-bib-0425] Yonezawa, K. , Y. Yamamoto , H. Yamamoto , et al. 2001. “Suppression of Tumor Necrosis Factor‐Alpha Production and Neutrophil Infiltration During Ischemia‐Reperfusion Injury of the Liver After Heat Shock Preconditioning.” Journal of Hepatology 35: 619–627. 11690708 10.1016/s0168-8278(01)00191-x

[cph470089-bib-0426] Yoshida, R. , M. Nakamura , and R. Ikegami . 2022. “The Effect of Single Bout Treatment of Heat or Cold Intervention on Delayed Onset Muscle Soreness Induced by Eccentric Contraction.” Healthcare (Basel) 10: 2556. 36554079 10.3390/healthcare10122556 PMC9778753

[cph470089-bib-0427] Yoshihara, T. , H. Naito , R. Kakigi , et al. 2013. “Heat Stress Activates the Akt/mTOR Signalling Pathway in Rat Skeletal Muscle.” Acta Physiologica (Oxford, England) 207: 416–426. 23167446 10.1111/apha.12040

[cph470089-bib-0428] Yuzefovych, L. V. , V. A. Solodushko , G. L. Wilson , and L. I. Rachek . 2012. “Protection From Palmitate‐Induced Mitochondrial DNA Damage Prevents From Mitochondrial Oxidative Stress, Mitochondrial Dysfunction, Apoptosis, and Impaired Insulin Signaling in Rat L6 Skeletal Muscle Cells.” Endocrinology 153: 92–100. 22128025 10.1210/en.2011-1442 PMC3249685

[cph470089-bib-0429] Zaccardi, F. , T. Laukkanen , P. Willeit , S. K. Kunutsor , J. Kauhanen , and J. A. Laukkanen . 2017. “Sauna Bathing and Incident Hypertension: A Prospective Cohort Study.” American Journal of Hypertension 30: 1120–1125. 28633297 10.1093/ajh/hpx102

[cph470089-bib-0430] Zeng, X. Y. , H. Wang , F. Bai , et al. 2015. “Identification of Matrine as a Promising Novel Drug for Hepatic Steatosis and Glucose Intolerance With HSP72 as an Upstream Target.” British Journal of Pharmacology 172: 4303–4318. 26040411 10.1111/bph.13209 PMC4556469

[cph470089-bib-0431] Zheng, Y. , C.‐N. Im , and J.‐S. Seo . 2006. “Inhibitory Effect of Hsp70 on Angiotensin II‐Induced Vascular Smooth Muscle Cell Hypertrophy.” Experimental & Molecular Medicine 38: 509–518. 17079867 10.1038/emm.2006.60

[cph470089-bib-0432] Zhong, M. , A. Orosz , and C. Wu . 1998. “Direct Sensing of Heat and Oxidation by Drosophila Heat Shock Transcription Factor.” Molecular Cell 2: 101–108. 9702196 10.1016/s1097-2765(00)80118-5

[cph470089-bib-0433] Zlokovic, B. V. 2011. “Neurovascular Pathways to Neurodegeneration in Alzheimer's Disease and Other Disorders.” Nature Reviews Neuroscience 12: 723–738. 22048062 10.1038/nrn3114 PMC4036520

[cph470089-bib-0434] Zou, J. , Y. Guo , T. Guettouche , D. F. Smith , and R. Voellmy . 1998. “Repression of Heat Shock Transcription Factor HSF1 Activation by HSP90 (HSP90 Complex) That Forms a Stress‐Sensitive Complex With HSF1.” Cell 94: 471–480. 9727490 10.1016/s0092-8674(00)81588-3

[cph470089-bib-0435] Zychowska, M. , P. Polrola , G. Chruscinski , J. Zielinska , and J. Goral‐Polrola . 2017. “Effects of Sauna Bathing on Stress‐Related Genes Expression in Athletes and Non‐Athletes.” Annals of Agricultural and Environmental Medicine 24: 104–107. 28378983 10.5604/12321966.1233977

[cph470089-bib-0436] Bain, A. R. , L. Nybo , and P. N. Ainslie . 2015. “Cerebral Vascular Control and Metabolism in Heat Stress.” Comprehensive Physiology: 1345–1380. 26140721 10.1002/cphy.c140066

[cph470089-bib-0437] Crandall, C. G. , and T. E. Wilson . 2015. “Human Cardiovascular Responses to Passive Heat Stress.” Comprehensive Physiology 5: 17–43. 25589263 10.1002/cphy.c140015 PMC4950975

[cph470089-bib-0438] Havenith, G. , and D. Fiala . 2015. “Thermal Indices and Thermophysiological Modeling for Heat Stress.” Comprehensive Physiology: 255–302. 10.1002/cphy.c140051 26756633

[cph470089-bib-0439] Johnson, J. M. , C. T. Minson , and D. L. Kellogg Jr. 2011. “Cutaneous Vasodilator and Vasoconstrictor Mechanisms in Temperature Regulation.” 4: 33–89. 10.1002/cphy.c130015 24692134

[cph470089-bib-0440] Taylor, N. A. S. 2014. “Human Heat Adaptation.” Comprehensive Physiology: 325–365. 24692142 10.1002/cphy.c130022

